# MicroRNA expression profile in head and neck cancer: *HOX*-cluster embedded microRNA-196a and microRNA-10b dysregulation implicated in cell proliferation

**DOI:** 10.1186/1471-2407-13-533

**Published:** 2013-11-09

**Authors:** Patricia Severino, Holger Brüggemann, Flavia Maziero Andreghetto, Carme Camps, Maria de Fatima Garrido Klingbeil, Welbert Oliveira de Pereira, Renata Machado Soares, Raquel Moyses, Victor Wünsch-Filho, Monica Beatriz Mathor, Fabio Daumas Nunes, Jiannis Ragoussis, Eloiza Helena Tajara

**Affiliations:** 1 Albert Einstein Research and Education Institute, Hospital Israelita Albert Einstein, Sao Paulo, Brazil; 2 Department of Biomedicine, Aarhus University, Aarhus, Denmark; 3 Genomics Group at the Wellcome Trust Centre for Human Genetics, University of Oxford, Oxford, UK; 4 Radiation Technology Center (CTR), Nuclear and Energetic Research Institute IPEN/CNEN, Sao Paulo, Brazil; 5 Division of Head and Neck Surgery, Department of Surgery, School of Medicine, University of Sao Paulo, Sao Paulo, Brazil; 6 Department of Epidemiology, Faculty of Public Health, University of Sao Paulo, Sao Paulo, Brazil; 7 Department of Stomatology, Faculty of Dentistry, University of Sao Paulo, Sao Paulo, Brazil; 8 Department of Molecular Biology, School of Medicine, Sao Jose do Rio Preto, Sao Paulo, Brazil

## Abstract

**Background:**

Current evidence implicates aberrant microRNA expression patterns in human malignancies; measurement of microRNA expression may have diagnostic and prognostic applications. Roles for microRNAs in head and neck squamous cell carcinomas (HNSCC) are largely unknown. HNSCC, a smoking-related cancer, is one of the most common malignancies worldwide but reliable diagnostic and prognostic markers have not been discovered so far. Some studies have evaluated the potential use of microRNA as biomarkers with clinical application in HNSCC.

**Methods:**

MicroRNA expression profile of oral squamous cell carcinoma samples was determined by means of DNA microarrays. We also performed gain-of-function assays for two differentially expressed microRNA using two squamous cell carcinoma cell lines and normal oral keratinocytes. The effect of the over-expression of these molecules was evaluated by means of global gene expression profiling and cell proliferation assessment.

**Results:**

Altered microRNA expression was detected for a total of 72 microRNAs. Among these we found well studied molecules, such as the miR-17-92 cluster, comprising potent oncogenic microRNA, and miR-34, recently found to interact with p53. *HOX*-cluster embedded miR-196a/b and miR-10b were up- and down-regulated, respectively, in tumor samples. Since validated *HOX* gene targets for these microRNAs are not consistently deregulated in HNSCC, we performed gain-of-function experiments, in an attempt to outline their possible role. Our results suggest that both molecules interfere in cell proliferation through distinct processes, possibly targeting a small set of genes involved in cell cycle progression.

**Conclusions:**

Functional data on miRNAs in HNSCC is still scarce. Our data corroborate current literature and brings new insights into the role of microRNAs in HNSCC. We also show that miR-196a and miR-10b, not previously associated with HNSCC, may play an oncogenic role in this disease through the deregulation of cell proliferation. The study of microRNA alterations in HNSCC is an essential step to the mechanistic understanding of tumor formation and could lead to the discovery of clinically relevant biomarkers.

## Background

MicroRNAs (miRNAs) are ~22 nt non-coding RNA molecules that negatively regulate gene expression by degrading or destabilizing the messenger RNA (mRNA) or by inhibiting protein translation [1]; some reports demonstrate that they may also function as positive regulators [2,3]. MiRNAs have been shown to contribute to cancer development and progression, and are differentially expressed between normal tissues and cancers [4]. Although the function of most of the miRNAs identified to date has yet to be determined, their use as potential biomarkers or therapeutic targets has been considered in several human diseases and cancers [5,6].

Head and neck squamous cell carcinoma (HNSCC) is a significant public health entity, representing the sixth leading cancer by incidence worldwide [7,8]. Genetic changes that bring about HNSCC are usually a consequence of continued exposure to carcinogens associated with tobacco. Despite advances in medical and surgical treatment, the overall 5-year survival rate for patients with HNSCC remains around 50% [8]. A recent work by Liu *et al*., 2009 [9] analyzed data compiled by the American Cancer Society and points out that new cases of HNSCC increased 25% during the past 5 years, highlighting the need for a better understanding of the molecular events leading to the development of this disease.

The number of studies addressing the contribution of miRNA deregulation in the context of HNSCC is, however, limited [10,11]. Some of these studies have evaluated the potential use of miRNAs as biomarkers with clinical application, associating the expression levels of some of these miRNAs with survival rates or metastatic potential [12-16]. Overall, results are promising, but still preliminary and lacking corroboration.

In our study we determined the miRNA expression profile of oral squamous cell carcinoma (OSCC) samples, a type of cancer that represents 90% of all HNSCC [9]. We also performed functional assays for two differentially expressed miRNAs, miR-196 and miR-10b, since neither have been previously associated with HNSCC. Despite studies linking these miRNAs to the regulation of homeobox (*HOX*) genes [17,18] our data suggest that, in the case of HNSCC, they have little impact in *HOX* gene expression. We demonstrate that miR-10b and miR-196a interfere in cell proliferation through distinct processes and in a cell-type dependent manner.

## Methods

### Samples

Fifteen patients with OSCC (tongue and floor of the mouth) were selected for the microarray experiments. In order to validate the microarray results, 35 additional patients with HNSCC (oral cavity, oropharynx and larynx) were selected. The clinical and pathological profile of patients is shown in Table 1. The average age of patients was 55.5 years (SD 9.8, range 38–82 years), and the male/female ratio was 24:1. Most patients were smokers or former smokers and had a history of chronic alcohol abuse. Tumor and corresponding cancer free surgical margins containing the corresponding epithelium were collected from patients submitted to surgical resection of primary tumor at Hospital das Clinicas, Hospital Heliopolis and Arnaldo Vieira de Carvalho Cancer Institute, in Sao Paulo, Brazil. All patients provided written informed consent, and the research protocol was approved by review boards of all institutions involved and by the National Committee of Ethics in Research (CONEP 1763/05). Samples corresponding to the oral cavity, base of the tongue and larynx were snap-frozen in liquid nitrogen immediately after surgery and stored in liquid nitrogen until RNA preparation. Frozen samples were sectioned using a cryostat, and tissue sections were stained with RNAse-free reagents. Analysis of hematoxylin and eosin-stained sections by the study pathologists confirmed >75% tumor cells in all HNSCC samples and that surgical margins were tumor-free. The diagnosis of HNSCC was confirmed, and tumors were histologically examined for perineural invasion (tumor cells in the perineural space or epineurium), tumor differentiation (well, moderated or poorly differentiated, according to the WHO guidelines), lymphatic-vascular invasion, surgical margins, and lymph node metastasis. Tumors were staged according to the TNM clinical staging system, as proposed by the International Union Against Cancer.

**Table 1:** Clinical and pathological data of patients in this study

**Patient**	**Site**	**Gender**	**Age (yr)**	**Pathologic stage**	**Histological differentiation**
1	OC-FOM	Male	72	T2N0M0	Moderate
2	OC-T	Male	55	T3N0M0	Moderate
3	OC-FOM	Male	75	T2N0M0	Moderate
4	OC-FOM	Male	46	T1N2bM0	Well
5	OC-T	Male	66	T3N0M0	Moderate
6	OC-T	Male	59	T2N2cM0	Well
7	OC-T	Male	49	T4aN2bM0	Well
8	OC-FOM	Male	53	T4aN1M0	Moderate
9	OC-T	Female	82	T1N0M0	Well
10	OC-FOM	Male	69	T4aN2cM0	Moderate
11	OC-T	Male	52	T3N2cM0	Moderate
12	OC-FOM	Male	59	T4aN2bM0	Moderate
13	OC-FOM	Male	56	T4aN3M0	Moderate
14	OC-FOM	Male	51	T2N0M0	Moderate
15	OC-FOM	Male	54	T4aN2bM0	Well
16	OC-T	Male	55	T2N0M0	Moderate
17	OC-T	Male	43	T3N0M0	Moderate
18	OP-BOT	Male	47	T4aN0M0	Well
19	OC-FOM	Male	64	T4aN2cM0	Moderate
20	OC-T	Male	57	T4aN2bM0	Moderate
21	OC-FOM	Male	67	T3N0M0	Well
22	L	Male	64	T3N0M0	Moderate
23	OC-FOM	Male	44	T2N2bM0	Well
24	OC-FOM	Male	63	T3N2cM0	Moderate
25	OC-FOM	Male	40	T4aN0M0	Moderate
26	OC-FOM	Male	45	T4aN2cM0	Well
27	OC-FOM	Male	51	T4aN1M0	Moderate
28	OC-FOM	Male	56	T4aN0M0	Well
29	L	Male	68	T2N0M0	Well
30	OC-FOM	Male	63	T3N0M0	Moderate
31	OC-FOM	Male	46	T4aN3M0	Well
32	OC-FOM	Male	68	T4aN1M0	Well
33	OC-FOM	Male	40	T4aN2cM0	Moderate
34	OC-FOM	Male	61	T2N2bM0	Moderate
35	OP-BOT	Male	41	T3N0M0	Moderate
36	OC-FOM	Male	62	T3N0M0	Moderate
37	OP-BOT	Male	55	T2N3M0	Moderate
38	OP-BOT	Male	51	T3N3M0	Moderate
39	OC-FOM	Female	46	T2N0M0	Well
40	OP-BOT	Male	48	T2N2cM0	Moderate

*OC-T*, oral cavity – tongue; *OC-FOM*, oral cavity - floor of mouth; *OP-BOT*, oropharynx – Base of tongue; *L*, larynx. Patients 1–15 were used for microRNA gene expression profiling by means of microarray analysis.

### RNA isolation

Total RNA was prepared from tissue using mirVana miRNA Isolation Kit (Ambion, Austin, TX) in compliance with the manufacturer’s protocol. RNA integrity and concentration were assessed using the RNA 6000 Nano Assay kit with Agilent 2100 Bioanalyzer according to the manufacturer’s instructions (Agilent Technologies, Palo Alto, CA).

### miRNA microarray expression profiling

MiRNA expression profiling was performed using the Illumina miRNA arrays version 1.0. Sample preparation and hybridization followed the manufacturer’s instructions. Briefly, 200 ng of total RNA was first polyadenylated and converted to biotinylated cDNA, which was attached to a solid phase and hybridised with a pool of miRNA-specific oligonucleotides (MSO). Each single MSO is used to assay one miRNA on the panel. Universal PCR amplification was then performed, creating fluorescently labeled products identifiable by their unique MSO sequence. These products were hybridized on the Illumina miRNA array, with the address sequence from each MSO enabling the hybridization of specific miRNA products to specific locations on the BeadArray substrate. Hybridization signals were detected and quantified using Illumina scanner and BeadStudio version 3.2.7. Average signals were quantile normalized and then filtered according their detection p-value: only miRNAs for which the detection p-value was consistently equal or lower than 0.05 across at least one group of samples (marginal and/or tumor samples) were considered expressed and further analyzed. All data is MIAME compliant and the raw data has been deposited in a MIAME compliant database (Gene Expression Omnibus database) under accession Number GSE31277. Differentially expressed miRNAs were determined by using the Rank Product, non-parametric statistical method based on ranks of fold-changes [19]. We used the RankProd package on R for this analysis; the percentage of false positives was calculated and p-values were accordingly corrected for multiple comparisons.

### Relative quantification of miRNA levels using real time-PCR

To validate the microarray expression data, miRNAs were subjected to quantitative Real Time-PCR using the TaqMan miRNA assay system (Applied Biosystems, Foster City, CA). Briefly, about 100 ng of total RNA was subjected to a reverse transcription reaction using miRNA-specific looped primers according to the manufacturer’s protocol to obtain the cDNA. Subsequent PCRs used miRNA specific forward and reverse primers along with appropriate cDNA product and TaqMan universal mix. PCR was carried out in AB7500 (Applied Biosystems, Foster City, CA) in a 20 ul volume reaction following thermal cycling parameters suggested by the manufacturer: 50°C for 2 min, 95°C for 10 min and 45 cycles of 95°C for 15 s and 60°C for 1 min.

The expression data was normalized to the RNU48 expression. RNU48 was chosen as a suitable endogenous control gene following analysis of gene expression stability of three candidate genes across our samples. For a detailed description of this step refer to the next Methods’ section. Expression levels were determined using the comparative ∆Ct method [20].

For miRNAs individually studied in independent sets of samples by quantitative real-time PCR, the nonparametric test Wilcoxon Signed Rank Test was used to detect the statistically significant differences between paired normal tissue (N) and tumor (T) samples obtained from the same individual. This test was performed using SPSS for Windows® Software. The same software was used to calculate the mean and standard deviation of all variables.

### Identification of suitable endogenous control gene for microRNA gene expression analysis by real-time PCR

The expression of three snoRNAs (RNU6B, RNU44 and RNU48) was measured by quantitative real-time PCR with TaqMan miRNA assays (Applied Biosystems, Foster City, CA), as previously described for all samples assayed by miRNA microarrays. This data was analyzed using the SLqPCR package in R [21] to determine the expression stability of these snoRNAs across samples. The stability factor M was calculated for each snoRNA (M (for RNU48) = 0.69; M (for RNU44) = 0.78; M (for RNU6B) = 0.75). Since high expression stability is associated to low M values, RNU48 appeared to be the snoRNA with most stable expression across the set of samples analyzed, hence was chosen as control for normalisation.

### Prediction of miRNA targets and their functional analysis

Potential miRNA targets were identified using Ingenuity Pathway Analysis (IPA Ingenuity Systems, http://www.ingenuity.com). Only experimentally validated targets were selected, using miRecords (http://mirecords.biolead.org/), Tarbase (http://microrna.gr/tarbase) or TargetScan (http://www.targetscan.org/). For fuctional annotation of potential targets we used KEGG pathways term enrichment analysis using the computational tool Database for Annotation, Visualization and Integrated Discovery (DAVID) v6.7 (http://david.abcc.ncifcrf.gov/home.jsp).

### HNSCC cell line and keratinocyte cell culture

The HNSCC cell lines SCC25 and SCC9, derived from a SCC of the tongue, and FaDu, derived from a SCC of the hypopharynx were used in this study. They were obtained from American Type Culture Collection (SCC25 catalog number CRL-1628, SCC9 catalog number CRL-1629, and FaDu catalog number HTB-43). The cell lines were grown in a Dulbecco’s Modified Eagle’s medium/Nutrient Mixture F-12 Ham (DMEM/F12) supplemented with 10% fetal bovine serum in a humidified atmosphere of 5% CO^2^ and 95% air at 37°C. Oral keratinocytes were obtained from primary cultures of the buccal mucosa, from voluntary donor patients undergoing surgery performed in out-patient clinics in the Dentistry School of USP. The patients were informed and signed the required Informed Consent. This study was approved by the Research Ethics Committee of the *Instituto de Pesquisas Energéticas e Nucleares* (IPEN/CNEN-SP) [Institute of Energy and Nuclear Research] (approval number 087/CEP-IPEN/SP). Keratinocytes were plated on a support layer, called feeder-layer, composed of murine fibroblasts of the type 3T3-Swiss albino (ATCC, catalog number CCL-92), which were irradiated (60 Gy), and maintained in an incubator at 37°C, in a humidified atmosphere containing 5% CO_2_ and grown as previously described [22].

### Transfection of cultured cells for up-regulation of miRNAs

The siPORT *NeoFx* reagent (Ambion) was used for transfection following the manufacturer’s protocol. For up-regulation, the Ambion Pre-miR™ miRNA Precursor Molecule (hsa-miR-10b and hsa-miR-196a) was used, with Ambion’s Pre-miR negative control #1. Successful up-regulation was achieved with 50 nM of final Pre-miR miRNA Precursor concentration.

### Immunofluorescence assay for proliferation analysis

Normal keratinocytes transfected with the miRNA precursor and the negative control were cultured in Lab-Tek Chamber Slides (Nalge Nunc International, Rochester, NY, USA) for the immunofluorescence assay. Cells were fixed with methanol, blocked with 3% bovine serum in PBS, and incubated for 1 h with antihuman Ki67 (Monoclonal Mouse, clone MIB-1, DAKO, Denmark A/5), diluted 1:400. Cells were washed with PBS and incubated at room temperature for 45 minutes with secondary antibody conjugated with fluorescein (1:50) (Novocastra Laboratories, UK), in a dark chamber. Following washing, chambers containing the cells were mounted with VECTASHIELD Mounting Medium with DAPI (Vector Laboratories, Ind. Buelingame, CA 94010). Results were analyzed by fluorescence microscopy (Zeiss Axiophot II, Carl Zeiss, Oberköchen, Germany). The percentage of cells displaying Ki67 labeling was determined by counting the number of positive Ki67 stained cells as a proportion of the total number of cells counted. Cells were counted manually in the whole chamber area.

### Proliferation assay by flow cytometry

Cell lines SCC9, SCC25 and FaDu were stained with Cell Trace Violet (Molecular Probes®), according to the manufacturer protocol. Briefly, the cells were incubated with 5 μM Cell Trace Violet for 20 minutes at 37°C, washed twice with fresh and warmed medium and cultured under regular conditions. The cells were run on BD LSR Fortessa flow cytometer with 405 nm laser at day zero and after 72 hours of cell culture for cell proliferation rate assessment. Proliferation rate was determined by fluorescence decay. Analysis was performed using Flow Jo software (Tree Star™). For cell proliferation rates after transfection, cell lines SCC25 and FaDu were stained 24 h after transfection (at the time of medium exchange). Proliferation rates were compared between scramble (negative control) and cells overexpressing miR-10b.

### mRNA microarray expression profiling and analysis

Following the transfection assays, the global gene expression analysis was conducted using the Agilent Human Whole Genome Oligonucleotide Microarray (44K; Agilent Technologies) following the manufacturer’s protocols. Oligonucleotide microarrays were scanned using the GenePix 4000B Microarray Scanner (Molecular Devices) and features were automatically extracted and analyzed for quality control using Agilent Feature Extraction Software. Raw data was deposited in a MIAME compliant database (Gene Expression Omnibus database) under the accession Number GSE31277. Partek Genomics Suite 6.6 (Partek Incorporated) was used for normalization of gene expression levels and for fold-change in gene expression calculation. To gain insights into the potential mechanisms affected by the overexpression of the miR-10b and miR-196a in cells, deregulated genes were mapped to regulatory networks using Ingenuity Pathway Analysis (IPA Ingenuity Systems, http://www.ingenuity.com).

### Western blotting

Western blotting was performed using a specific antibody against annexin 1 (1:1000 dilution) (Zymed Laboratories - 713400), and *β*-Actin (1:12.000 dilution) (Cell Signaling Technology, Danvers, MA, USA). Briefly, 72 hours after transfection cells were lysed in RIPA buffer (150 mM NaCl, 10 mM Tris/HCl, pH 7.4, 0.5% Triton X-100 and protease and phosphatase inhibitors (Biogene). Protein concentration was estimated using the BCA Protein Assay Kit (BioAgency, London, UK). 20 ug of protein lysate was separated in 15% SDS gel (GE Healthcare, Piscataway, NJ, USA) and subsequently transferred to nitrocellulose membrane of 0,45 μm (GE Healthcare, Piscataway, NJ, USA). The membranes were blocked using 3% non-fat dry milk, and incubated with primary antibodies overnight at 4°C. The membranes were washed in 1x TBS eith 0.1% Tween-20 (TBS/T), incubated for 1 h with anti-rabbit secondary antibodies conjugated to horseradish peroxidase (Abcam - ab102779) and visualized with a chemiluminescence reagent (ECL) system (Amersham Biosciences, Arlington Heights, IL).

## Results and discussion

### MiRNA deregulation in OSCC samples: implication in tumor progression

HNSCC can involve multiple anatomical sites, each with individual molecular characteristics, and highly affected by the drinking and smoking habits of patients [13,23,24]. In an attempt to limit data variability due to HNSCC subsites and environmental factors, we assessed miRNA expression levels in 15 OSCC samples limited to tongue and floor of the mouth, from patients possessing similar demographic and clinico-pathological characteristics (Table 1, detailed in Methods). Samples were paired with tumor-free surgical margins. The expression profiles of tumor samples revealed significant differential expression for 72 miRNAs compared to their corresponding tumor-free margins (Table 2). Several studies have analysed the miRNA expression profile of OSCC cell lines and tumor samples, with little overlap among results [25,26]. This inconsistency in results justifies additional studies.

**Table 2:** Deregulated miRNAs between 15 OSCC and 15 tumor-free surgical margins

**miRNA**	**Fold-change**	**P value**	**miRNA**	**Fold-change**	**P value**
**hsa-miR-196a**	7.94	0	**hsa-miR-1**	−6.67	0
**hsa-miR-33**	5.51	0	**hsa-miR-30a-3p**	−3.34	0
**hsa-miR-19a**	3.29	0	**hsa-miR-139**	−3.13	0
**hsa-miR-33b**	2.65	0	**hsa-miR-133a**	−3.79	0
**hsa-miR-142-5p**	2.81	0	**hsa-miR-486**	−3.02	0
**hsa-miR-503**	2.53	0	**hsa-miR-135a**	−3.47	0
**hsa-miR-31**	2.40	0	**hsa-miR-204**	−2.68	0
**hsa-miR-7**	2.33	0	**hsa-miR-206**	−3.42	0
**hsa-miR-19b**	2.66	0	**hsa-miR-411**	−2.67	0
**hsa-miR-135b**	2.29	0	**hsa-miR-499**	−2.64	0
**hsa-miR-632**	2.15	0	**hsa-miR-10b**	−2.39	0
**hsa-miR-504**	1.99	0	**hsa-miR-99a**	−2.35	0
**hsa-miR-187**	2.10	0	**hsa-miR-299-5p**	−2.46	0
**hsa-miR-339**	2.12	1.00E-04	**hsa-miR-379**	−2.42	0
**hsa-miR-302d**	1.93	1.00E-04	**hsa-miR-100**	−2.17	0
**hsa-miR-34b**	2.06	1.00E-04	**hsa-miR-30a-5p**	−2.13	0
**hsa-miR-34c**	2.02	2.00E-04	**hsa-miR-95**	−2.16	1.00E-04
**hsa-miR-455**	2.06	3.00E-04	**hsa-miR-378**	−2.08	1.00E-04
**hsa-miR-9**	1.99	3.00E-04	**hsa-miR-218**	−1.88	1.00E-04
**hsa-miR-296**	1.93	3.00E-04	**hsa-miR-368**	−2.00	2.00E-04
**hsa-miR-301**	2.02	3.00E-04	**hsa-miR-363**	−1.83	2.00E-04
**hsa-miR-130b**	1.97	3.00E-04	**hsa-miR-128a**	−1.90	4.00E-04
**hsa-miR-196b**	1.93	4.00E-04	**hsa-miR-655**	−1.94	6.00E-04
**hsa-miR-200a**	1.96	4.00E-04	**hsa-miR-376a**	−1.83	1.00E-03
**hsa-miR-210**	1.87	7.00E-04	**hsa-miR-628**	−1.87	1.00E-03
**hsa-miR-17-3p**	1.87	9.00E-04	**hsa-miR-487b**	−1.95	1.00E-03
hsa-miR-302b*	1.76	9.00E-04	**hsa-miR-410**	−1.81	1.00E-03
**hsa-miR-224**	1.59	9.00E-04	**hsa-miR-140**	−1.79	2.00E-03
**hsa-miR-183**	1.78	9.00E-04	**hsa-miR-801**	−1.34	2.00E-03
**hsa-miR-138**	1.79	1.00E-03	hsa-miR-376a*	−1.80	2.00E-03
**hsa-miR-188**	1.63	2.00E-03	**hsa-miR-154**	−1.76	3.00E-03
**hsa-miR-92b**	1.66	2.00E-03	**hsa-miR-432**	−1.80	3.00E-03
**hsa-miR-182**	1.66	2.00E-03	**hsa-miR-328**	−1.64	3.00E-03
**hsa-miR-144**	1.42	3.00E-03			
**hsa-miR-146b**	1.42	4.00E-03			
hsa-miR-182*	1.61	4.00E-03			
**hsa-miR-149**	1.53	4.00E-03			
**hsa-miR-141**	1.67	4.00E-03			
**hsa-miR-610**	1.34	5.00E-03			

Negative Fold-Change indicates over-expression in margins. p-values indicate the significance level for each gene and have been multiple-test-corrected using Rank Products as described in Methods. *: identifies the star strand of a miRNA.

In order to access biological processes possibly targeted by deregulated miRNAs we performed a functional analysis of validated targets through KEGG term enrichment analysis using the computational tool DAVID. Thirty-eight of the 72 deregulated miRNAs possessed mRNA targets that have been experimentally observed; in total 609 genes are potentially regulated (Additional file 1). These genes were mapped to KEGG pathways and were shown to be broadly involved in cancer development (Additional file 2).

Specifically, members of the miR-17-92 cluster were deregulated in our dataset: miR-19a and miR-19b were strongly up-regulated, in addition to moderate up-regulation of miR-17-3p/miR-17-5p and miR-92b. These results are in line with the observation that the miR-17-92 cluster is up-regulated in many cancer types, including lung cancer and lymphoma [27,28]. Accordingly, miR-17-92 cluster members have been shown to take part in feedback loops determining the role of c-MYC as tumor suppressor and/or oncogene [29,30]. Specifically, c-MYC apparently possesses a tumorigenic role in HNSCC, constituting a current candidate for anticancer strategies [31]. Recently, the miR-17-92 cluster has been also shown to regulate multiple components of the TGF-β pathway in neuroblastoma [32]. Other cancer-related miRNAs up-regulated in our OSCC samples are members of the miR-34 family: miR-34b and miR-34c. To our knowledge this is the first report of their altered expression profile in HNSCC, although the deregulation of miR-34a has been recently addressed in HNSCC [33]. These results are interesting in light of the finding that miR-34 is a direct target of p53, functioning downstream of the p53 pathway as a tumor suppressor [34,35]. Similar to other types of cancer, inactivation of p53 is an extremely common event in head and neck cancers, with mutant p53 status found in nearly 50% of the cases and commonly associated with poor prognosis [36]. However, the role of miR-34b/c in the context of p53 regulation has not been addressed in HNSCC.

In agreement with most miRNA profiles in HNSCC samples and tumor cell lines, miR-133a was also down-regulated in our cancer set as compared to tumor-free samples. Its tumor suppressor activity, for instance by controlling the target genes actin-related protein 2/3 complex subunit 5 (ARPC5) and moesin (MSN), has been already demonstrated in squamous cell carcinoma of the tongue [37-39]. Since this seems to be a robust characteristic in HNSCC, its function should be further investigated as well as its possible use as a biomarker for early cancer detection.

### Deregulation of homeobox cluster-encoded miRNAs miR-196a/b and miR-10b

**Figure 1:**
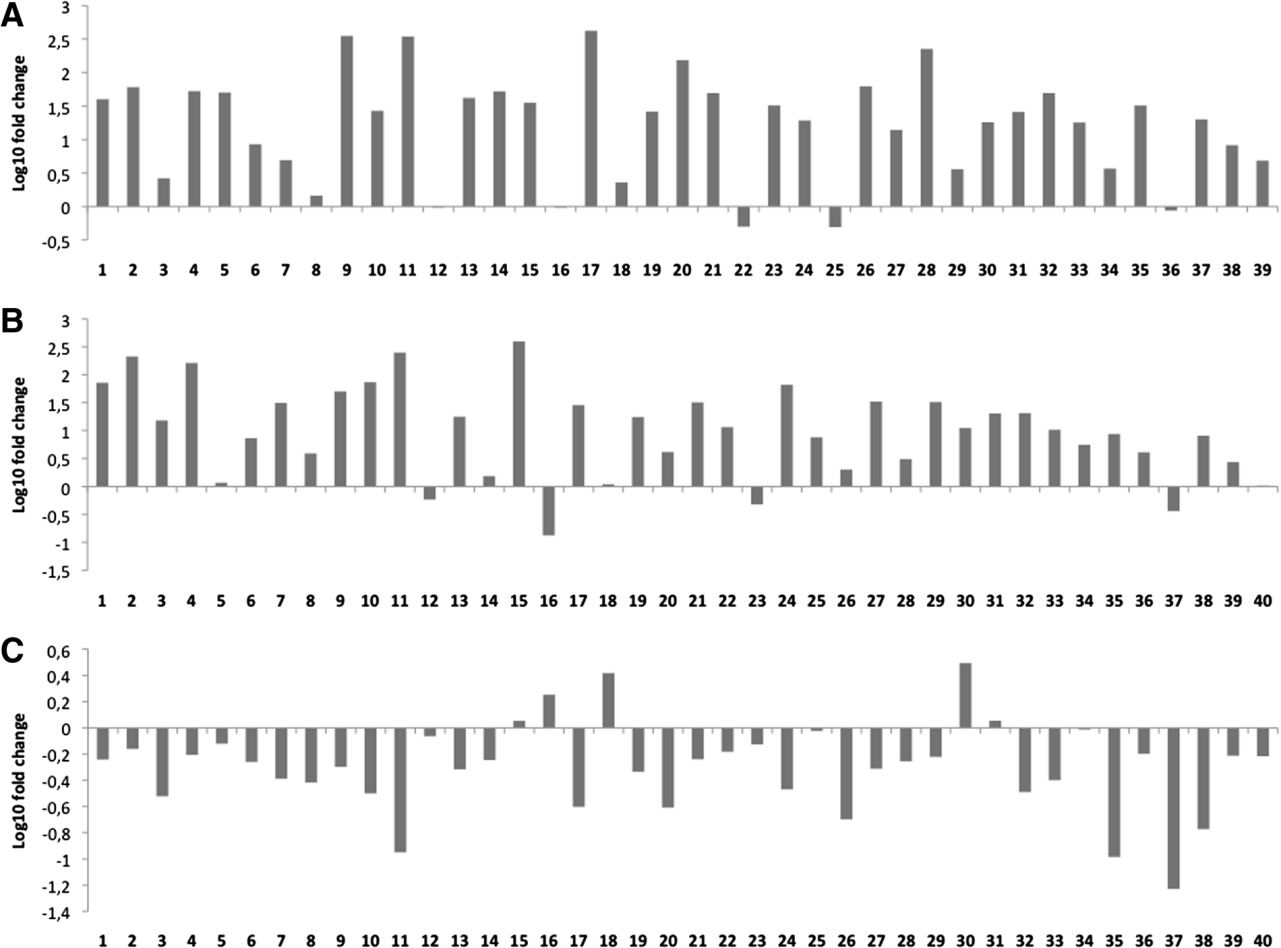
**MiR-196 and MiR-10b are deregulated in HNSCC as measured by relative qRT-PCR. A**: MiR-196a, miR-10b expression in thirty-nine paired tissue samples (fold-change between cancer and adjacent normal mucosa) of primary HNSCC. **B**: MiR-196b expression in forty paired tissue samples (fold-change between cancer and adjacent normal mucosa) of primary HNSCC. **C**: MiR-10b expression in forty paired tissue samples (fold-change between cancer and adjacent normal mucosa) of primary HNSCC. Wilcoxon Signed Rank Test was used to evaluate the difference in gene expression levels between cancer and normal tissue and a statistically significant difference was found between cancer and cancer-free tissue for the expression levels of the three tested miRNAs (p < 0.05).

MiR-196a/b was over-expressed and miR-10b was down-regulated in the OSCC samples compared with tumor-free surgical margins (Table 2). Both miRNAs are dysregulated in a variety of cancers [40,41], but have not been previously associated with OSCC. We validated our microarray results in an additional subset of OSCC samples as well as in samples belonging to other HNSCC subsites (Figure 1, and Table 1 for sample characteristics). Both miRNAs clearly presented differential expression between tumor and tumor-free samples, suggesting a role in HNSCC.

MiR-196a/b and miR-10b are embedded within homeobox (*HOX*) clusters of developmental regulators [42]*.* Schimanski and collaborators [43] demonstrated that *HOX* genes are targeted by miR-196, and *HOX* transcripts were also experimentally validated as miR-10 targets [44,45]. Given that molecular events involved in carcinogenesis interfere in the regulation of cell identity, it has been proposed that HOX proteins could be oncogenic regulators [46]. HOX genes have not been implicated in the development of HNSCC, as judged from reviewing the available literature, including HNSCC gene expression profiles. This suggests that the homeobox-cluster embedded miRNA could have a different role in HNSCC. Thus, we performed gain-of-function experiments aiming to outline a possible role for these molecules.

### Gain-of-function of miR-10b and miR-196a lead to impaired cell proliferation

Precursor molecules of miR-10b were transfected into SCC25 and SCC9 (tongue squamous cell carcinoma-derived cell lines) and FaDu (a cell line derived from hypopharyngeal squamous cell carcinoma), while miR-196a precursor molecules were transfected into human keratinocytes derived from normal oral epithelium. We chose SCC cell lines and oral keratinocytes as models for the investigation of miRNA function in a HNSCC genetic background, emulating cancer and tumor-free cellular systems, respectively.

**Figure 2:**
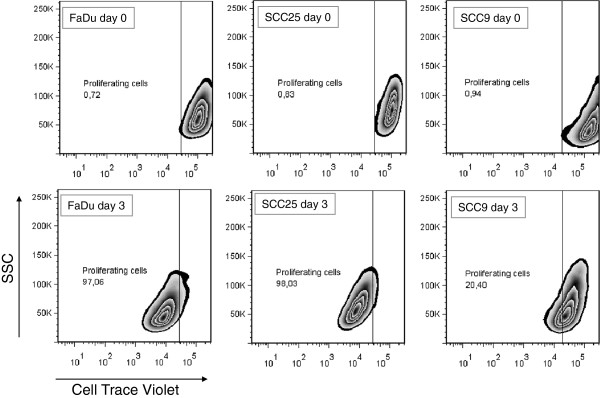
**Proliferation rate of SCC9, SCC25 and FaDu cell lines as determined by flow cytometry.** Proliferation rate was determined by fluorescence decay from measures at day 0 to day 3 (72 h). Numerical results are presented in Table 3.

Two SCC cell lines were initially chosen for the gain-of-function experiments due to differences in their proliferation rates, as reported in Figure 2 and Table 3, a characteristic that could affect results.

**Table 3:** Assessment of the number of cell divisions after 72h of cell culture

	**Initial**	**Final MFI**	**Predicted # cell divisions**
FaDu	109,219.96	6,826.24	3-4
SCC25	102,353.5	6,397.08	3-4
SCC9	152,640.3	19,080.0	2-3

Prediction of the number of cell divisions according to formula *final MFI = initial MFI/2n. MFI*, mean fluorescence index; *n*, number of cell divisions. final MFI = initial MFI/2^n^.

As expected, in untreated SCC cell lines, miR-196 was up-regulated and miR-10b was downregulated when expression levels were compared to untreated keratinocytes (data not shown).

**Figure 3:**
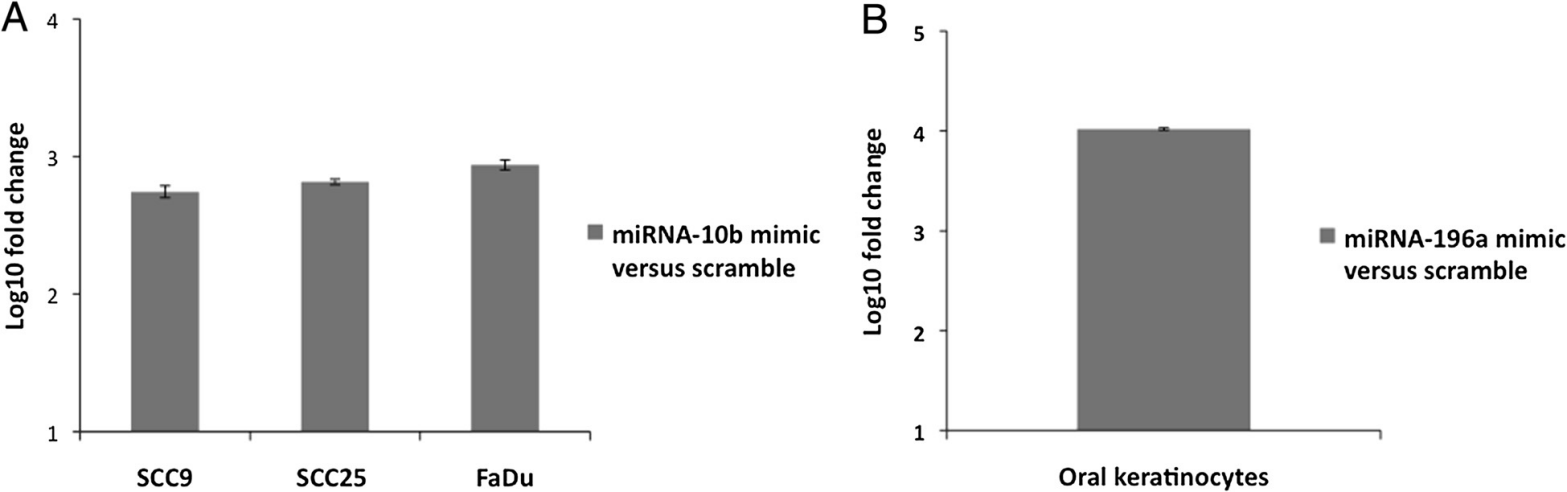
**Expression of miR-10b in the cell lines SCC25, SCC9 and FaDu, and of miR-196a in normal keratinocytes following transfection with the specific miRNA precursor molecules. A**: Expression of miR-10b in the cell lines SCC25, SCC9 and FaDu following transfection with the specific miRNA precursor molecule; **B**: Expression of of miR-196a in normal keratinocytes following transfection with the specific miRNA precursor molecule. Scramble represents cells transfected with a random sequence of precursor miRNA molecules validated by the manufacturer to not produce identifiable effects on known miRNA function. Fold change compares expression levels in transfected and scramble.

After transfection, we assessed the over-expresssion of the respective mature miRNAs in each cell line (Figure 3). Despite the successful overexpression of miR-10b in SCC9, these cells were very sensitive to the transfection, with intense effects in cell proliferation seen also for the negative control. Thus this cell line was not used in subsequent experiments.

**Figure 4:**
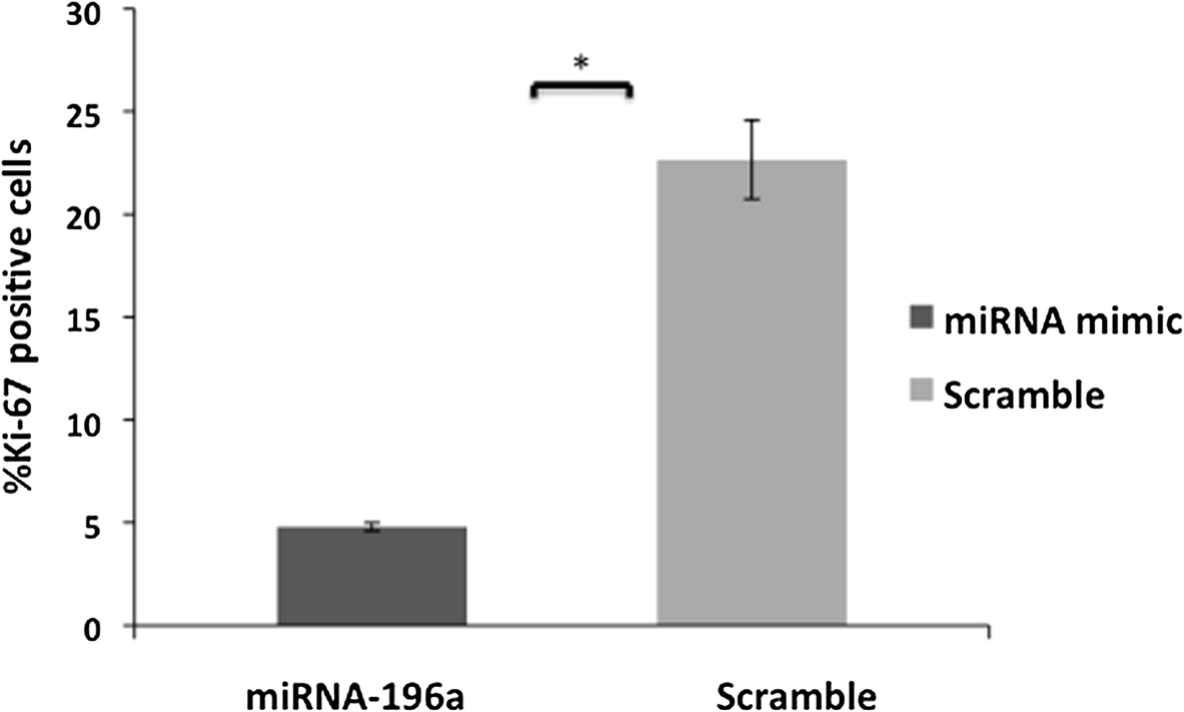
**Ki67 proliferation marker was detected by immunocytochemistry in keratinocytes.** A significantly lower number of Ki67-positive cells were observed upon over-expression of the miR-196a (*p < 0.05). The bars represent standard deviation and t*-*test was used for statistical analysis.

Since tumor cells evade programmed cell death and sustain proliferative status [47], we tested whether miR-10b and/or miR-196a could play a role in this scenario. Assessment of Ki-67 antigen expression, a cell proliferation marker, revealed that keratinocytes over-expressing miR-196a were mostly quiescent, as defined by a lack of Ki-67 antigen expression. When compared to transfection controls, cell proliferation was reduced approximately 5 fold in keratinocytes over-expressing miR-196a (Figure 4).

Despite inhibiting cell proliferation, specific occurrences at the cell surface level, such as surface blebbing, considered as a marker for apoptosis, were absent at 72 h after transfection (data not shown). Changes in nuclear morphology indicating late apoptosis were also absent.

**Figure 5:**
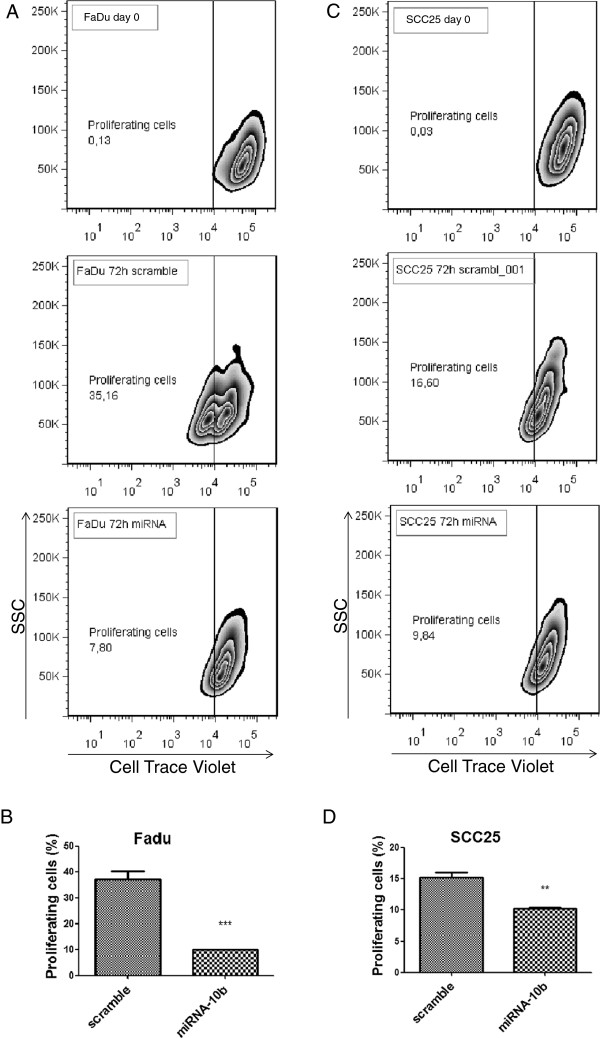
**miRNA-10b over expression decreases proliferation rate of head and neck cancer cells.** Fadu and SCC25 cell lines were transfected with miR-10b precursor and negative control (scramble) and the proliferation rate was measured for 72 hours. **A** and **C**: Zebra plot showing the fluorescence decay after 72 hours post-transfection with miRNA in Fadu and SCC25 cell lines, respectively. **B** and **D**: Graph representing the percentage of proliferating cells after 72 hours post-transfection with miRNA. The bars represent standard deviation and t*-*test was used for statistical analysis. **:p < 0.01. **:p < 0.001.

Inhibition of cell proliferation upon over-expression of miR-10b in SCC25 and FaDu was assessed by flow cytometry (Figure 5). This is consistent with the miRNA expression data: miR-10b was detected in low levels in HNSCC in this study, a downregulation that would thwart the inhibitory effect of miR-10b on cell proliferation. The effect of the overexpression of miR-10b was clearly more intense in FaDu as compared to SCC25.

Given the fact that miR-196a was found to be overexpressed in HNSCC samples, the inhibitory effect of miR-196a overexpression on proliferation of normal keratinocyes cannot easily put into context. One hypothesis could be that miR-196a overexpression in HNSCC could also be the consequence of uncontrolled proliferation, as a means to counteract it, rather than the cause of its perturbation.

Efforts to understand the global effects of miR-196a, which might be cell-type dependent, are essential considering that it has been recently addressed as a potential therapeutic target [41].

### Gene expression profiles upon overexpression of miR-10b and miR-196a do not show regulation of known targets

The identification of miRNA target genes is critical in order to understand their roles. However, this task is challenging. MiRNAs are usually imperfectly complementary to the 3′UTR region of their mRNA targets in mammalian cells, with target effect hardly detected at the gene expression level. Additionally, the cellular environment is key in determining miRNA functions, which will vary depending on the cell type.

While keeping these drawbacks in mind, global gene expression profiling using DNA microarrays was performed in this study to identify deregulated cellular processes upon the overexpression of miR-10b and miR-196a in each cell model. Broad consequences of the overexpression should be detected by global gene expression profiling, even when the deregulation of specific target genes might not be detected by this kind of experiment.

Experimentally validated mRNA targets were searched in Tarbase and miRecord databases. None of the miR-10b targets *HOXA1*, *HOXD10* and *KLF4*[40,48,49] were affected at the mRNA level by the overexpression of miR-10b in SCC25 or FaDu (Additional files 3 and 4, respectively). The same was true for the miR-196a gene targets *ANXA1, HOXA7, HOXB8, HOXC8, HOXD8, KRT5* and *S100A9*[42,50,51] (Additional file 5). These results suggest that, at least at the mRNA levels these genes are not targeted by miR-196a in the cells used here.

**Figure 6:**
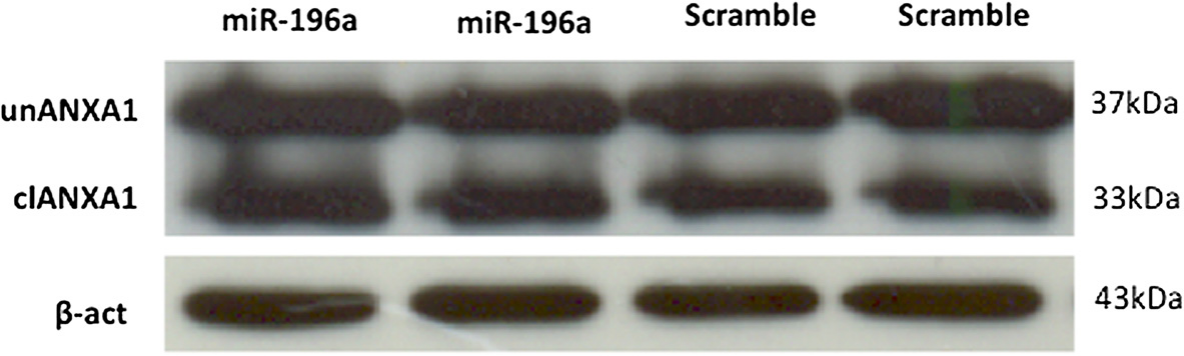
**Western blot analysis of ANXA1 expression in keratinocytes upon overexpression of miR-196a.** Total protein extracts from cells overexpressing miR-196a (miR-196a) and negative controls (scramble) were analyzed by western blot for ANXA1. Results show no differences in protein levels between the experiments.

Among the above mentioned gene targets, only ANXA1 down-regulation has been previously reported in HNSCC [52,53]. For this reason, we checked for alterations of this target also at the protein level. Our results demostrate that ANXA1 is not targeted by miR-196a under the conditions studied here (Figure 6).

### MiR10b and miR196a lead to cell cycle arrest through different mechanisms

**Figure 7:**
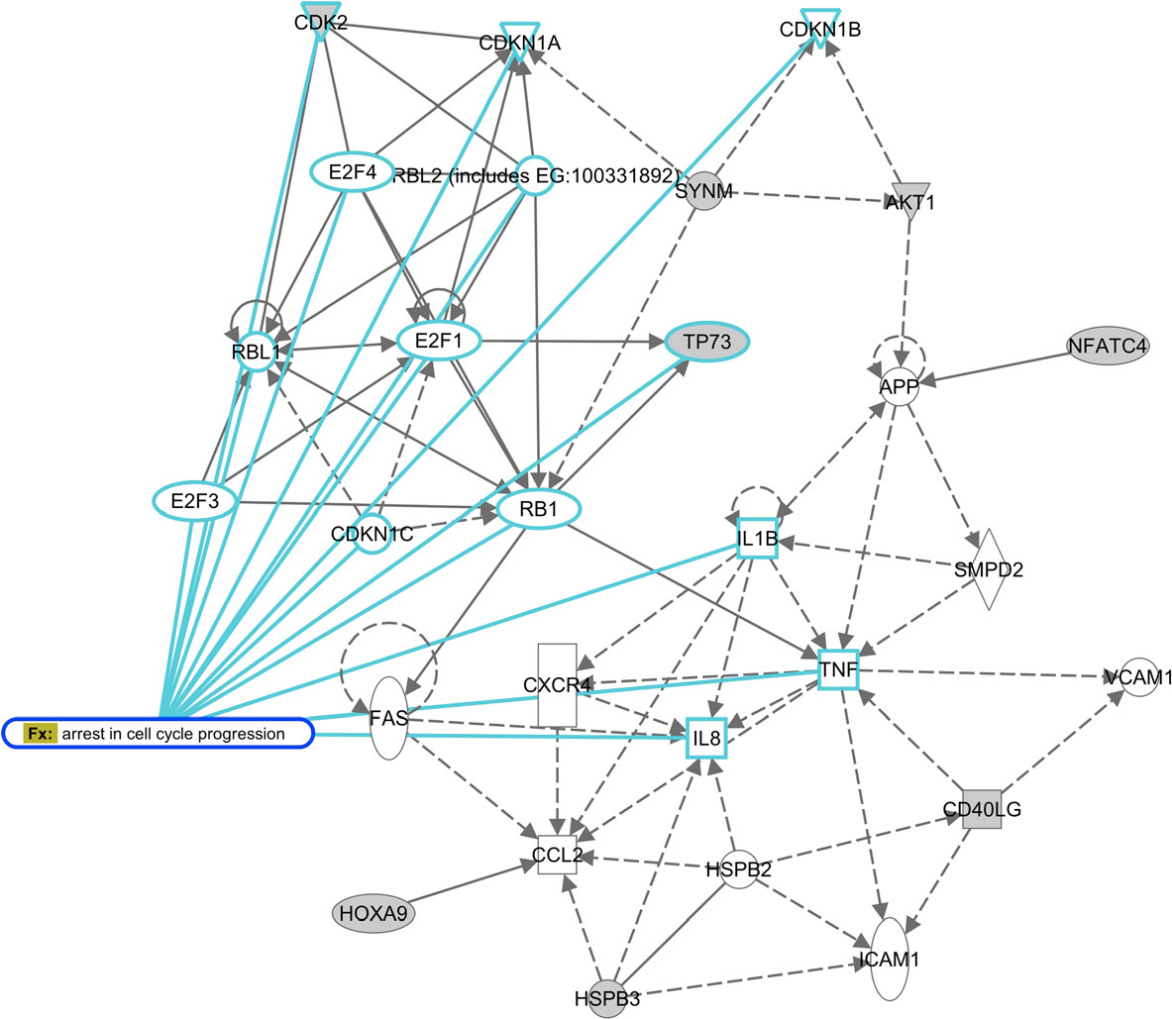
**Pathway analysis of deregulated genes upon miR-196a overexpression in keratinocytes.** Shown is the most significant interaction network based on deregulated genes in miR-196a overexpressing keratinocytes, calculated by IPA. The network is termed “DNA replication, recombination and repair, cell cycle”. In grey, deregulated genes from gene expression profiling.

We performed a functional analysis of deregulated genes aiming to pinpoint alterations that could explain impaired proliferation. A total of 353 annotated genes were downregulated (at 1.5 fold-change) following miR-196a over-expression in keratinocytes (Additional file 5). The relationships among these genes were assessed using Ingenuity Pathway Analysis (IPA), while considering only experimentally proven connections between genes or proteins. The most significant interaction network consisted of genes associated with DNA replication, recombination and repair, cell cycle and, consequently, cancer. Figure 7 depicts this network and genes involved in cell cycle arrest are highlighted. This network contains 8 deregulated genes from our dataset: *CDK2*, *SYNM* (*DMN*), *TP73*, *AKT1*, *NFATC4*, *HOXA9*, *HSPB3* and *CD40LG*. Of particular interest is the downregulation of CDK2 and *AKT1* and the upregulation of *TP73. CDK2* is a subunit of the cyclin-dependent protein kinase complex, expressed in G1-S phase, and essential for cell cycle G1/S phase transition. *TP73*, up-regulated in cells overexpressing miR-196a, transcriptionally activates target genes leading to apoptosis and growth arrest [54]. The activation of PI3K/AKT pathway in HNSCC is well known; the pathway regulates cell proliferation and has been addressed as a therapeutical target. Thus, the expression patterns of these three genes, following over-expression of miR-196a, would be in agreement with the observed arrest of the cell cycle. However, none of them are direct targets of this miRNA and further studies are needed in order to comprehend the observed effect.

Overexpression of miR-10b in SCC25 and in FaDu provided relatively similar results. Two hundred and ten annotated genes were downregulated and 169 were upregulated when SCC25 cells overexpressing miR-10b were compared to controls (Additional file 3) while 161 genes were downregulated and 169 upregulated in FaDu overexpressing miR-10b (Additional file 4), when at least a 2-fold difference was considered. Sixteen common genes were downregulated in both cell lines, but none of these genes were miR-10b predicted targets.

Regulatory networks provided by IPA did not contain a significant number of genes directly implicated in cell proliferation or cell cycle arrest for SCC25 cell line. This analysis, however, highlighted enrichment of terms belonging to the G-protein-coupled-receptor signaling pathway - with 9 molecules regulated in our dataset (ADRB3, AVPR2 and GRM2, upregulated 2-fold in SCC25 overexpressing miR-10b; and CNR1 (16-fold), DRD3 (4-fold), HCAR2 (2-fold) and OPRK1 (14-fold), downregulated in these cells). A recent review addresses mechanisms by which G-protein-coupled-receptors participate in the regulation of cell cycle [55] and, in the context of HNSCC, G-protein-coupled-receptors have been associated with EGFR signaling and cell survival [56].

**Figure 8:**
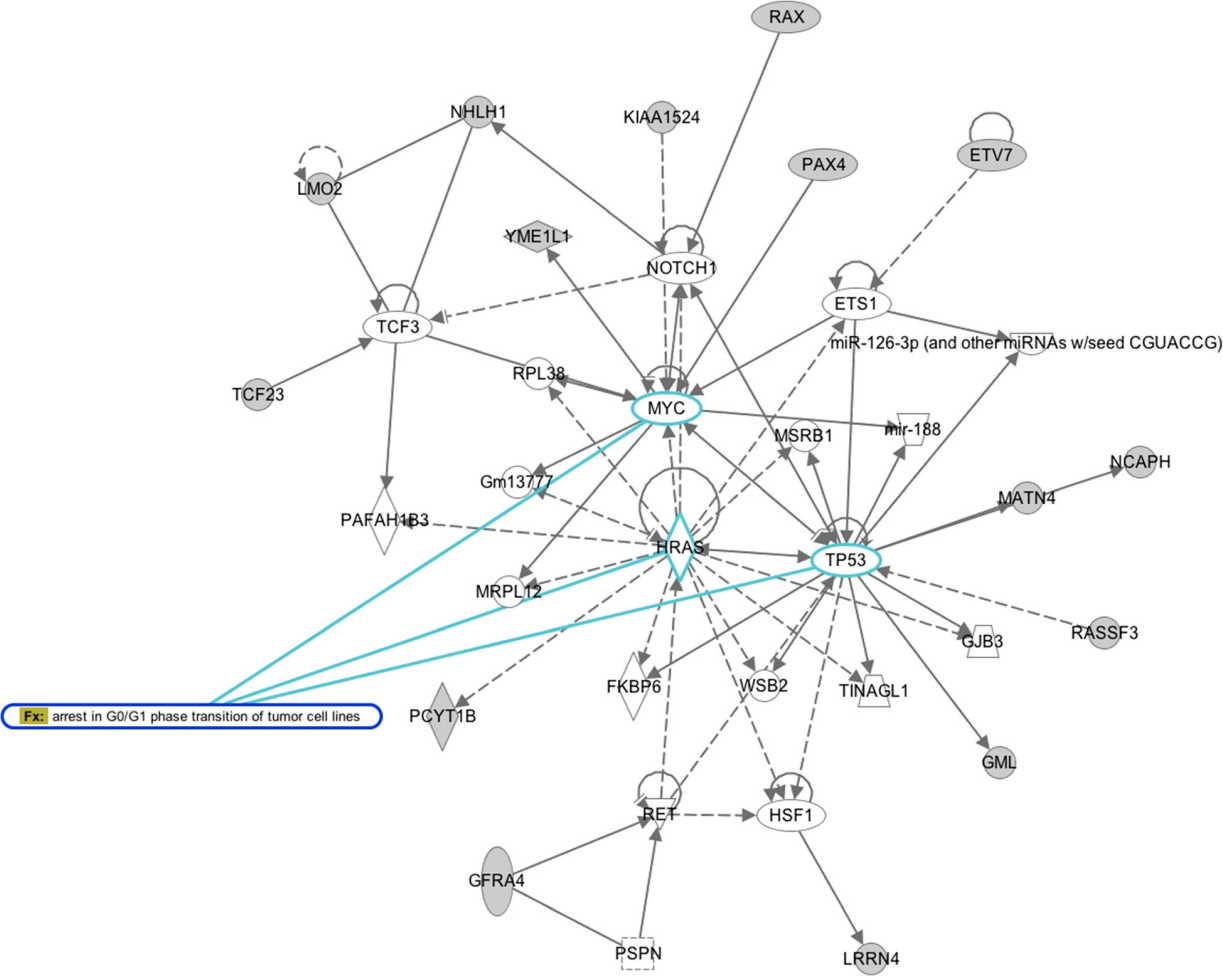
**Pathway analysis of deregulated genes upon miR-10b overexpression in FaDu cell line.** Shown is the most significant interaction network based on deregulated genes in miR-10b overexpressing FaDu cell line, calculated by IPA. The network includes genes involved in the regulation of cell cycle progression and arrest. In grey, deregulated genes from gene expression profiling.

A significant regulatory network built with deregulated genes upon overexpression of miR-10b in FaDu includes genes involved in the regulation of cell cycle progression and arrest (Figure 8). Although none of these genes have been implicated in HNSCC or heavily studied in the context of cancer, it is noteworthy the fact that they relate to cell cycle regulation through key players in HNSCC: TP53, NOTCH1, MYC and HRAS [57].

From this analysis it became clear that the effect of the overexpression of miR-10b in SCC25 and FaDu, and miR-196a in keratinocytes do not act upon a large number of cellular processes but may rather target a small set of genes, some of which directly or indirectly involved in the progression of cell cycle.

## Conclusions

Data on miRNA effects in tumorigenesis and cancer progression is still controversial and should vary with cell and cancer types. While individual miRNAs might possess numerous and distinct targets, they should be able to contribute to the same tumorigenic processes through complex, and still mostly unknown, networks. In HNSCC little is known about the contribution of miRNA to tumor development and progression, with several studies lacking corroboration. Besides presenting data matching to current knowledge, in this study we show that two miRNAs, miR-196a and miR-10b, play distinct roles in the regulation of cell proliferation within a HNSCC background.

## Competing interests

The authors declare there are no competing interests.

## Authors’ contributions

Conceived and designed the experiments: PS JR. Performed the experiments: PS CC FMA WOP RMS MFGK. Interpreted experimental data: PS CC HB. Clinical data analysis and sample selection: RM VWF EHT FDN. Wrote the paper: PS HB. All authors read and approved the final manuscript.

## Pre-publication history

The pre-publication history for this paper can be accessed here:

http://www.biomedcentral.com/1471-2407/13/533/prepub

## Supplementary Material

Additional file 1 **Experimentally validated targets for miRNAs deregulated between cancer and cancer-free samples.** Targets were selected using the tool MicroRNA Target Filter from Ingenuity Pathway Analysis. Click here for file

Additional file 2 **KEGG term enrichment analysis for gene targets of deregulated miRNAs between cancer and cancer-free samples.** KEGG term enrichment analysis were performed using DAVID Bioinformatics Resources (http://david.abcc.ncifcrf.gov/home.jsp). Click here for file

Additional file 3 Differentially expressed genes between SCC25 overexpressing miR-10b and transfection controls. Click here for file

Additional file 4 Differentially expressed genes between FaDu overexpressing miR-10b and transfection controls. Click here for file

Additional file 5 Differentially expressed genes between keratinocytes overexpressing miR-196a and transfection controls. Click here for file
