# Supplementary material for: MicroRNA expression profile in head and neck cancer: HOX-cluster embedded microRNA-196a and microRNA-10b dysregulation implicated in cell proliferation
Source: BMC Cancer. 2013 Nov 9;13:533. doi: 10.1186/1471-2407-13-533 (PMC3826519; doi:10.1186/1471-2407-13-533)
Supplement: Additional file 1 — Experimentally validated targets for miRNAs deregulated between cancer and cancer-free samples. Targets were selected using the tool MicroRNA Target Filter from Ingenuity Pathway Analysis. [file 1471-2407-13-533-S1.pdf]

miRNA	Source	Confidence	Symbol
**hsa-miR-1**	TarBase	Experimentally Observed	ABHD11
**hsa-miR-1**	TarBase TargetScan Human miRecords	Experimentally Observed	ACPL2
**hsa-miR-1**	TarBase TargetScan Human miRecords	Experimentally Observed	ADAR
**hsa-miR-1**	TarBase TargetScan Human	Experimentally Observed	ADPGK
**hsa-miR-1**	TarBase	Experimentally Observed	AGMAT
**hsa-miR-1**	TarBase	Experimentally Observed	AGRN
**hsa-miR-1**	TarBase TargetScan Human miRecords	Experimentally Observed	ANKIB1
**hsa-miR-1**	TarBase TargetScan Human miRecords	Experimentally Observed	ANKRD29
**hsa-miR-1**	TarBase TargetScan Human	Experimentally Observed	ANP32B
**hsa-miR-1**	TarBase	Experimentally Observed	ANPEP
**hsa-miR-1**	TarBase TargetScan Human	Experimentally Observed	ANXA2
**hsa-miR-1**	TarBase	Experimentally Observed	AP3B1
**hsa-miR-1**	TarBase TargetScan Human	Experimentally Observed	AP3D1
**hsa-miR-1**	TarBase TargetScan Human miRecords	Experimentally Observed	ARCN1
**hsa-miR-1**	TarBase TargetScan Human miRecords	Experimentally Observed	ARF3
**hsa-miR-1**	TarBase miRecords	Experimentally Observed	ARF4
**hsa-miR-1**	TarBase miRecords	Experimentally Observed	ARHGAP29
**hsa-miR-1**	TarBase TargetScan Human miRecords	Experimentally Observed	ARHGEF18
**hsa-miR-1**	TarBase	Experimentally Observed	ARID1A
**hsa-miR-1**	TarBase TargetScan Human	Experimentally Observed	ARID2
**hsa-miR-1**	TarBase TargetScan Human	Experimentally Observed	ASH2L
**hsa-miR-1**	TarBase	Experimentally Observed	ATP6V0A1
**hsa-miR-1**	TargetScan Human miRecords	Experimentally Observed	ATP6V1B2
**hsa-miR-1**	TarBase TargetScan Human miRecords	Experimentally Observed Moderate (predicted)	AXL
**hsa-miR-1**	TarBase	Experimentally Observed	BCKDHB
**hsa-miR-1**	miRecords	Experimentally Observed	BCL2
**hsa-miR-1**	Ingenuity Expert Findings TarBase TargetScan Human miRecords	Experimentally Observed	BDNF
**hsa-miR-1**	TarBase TargetScan	Experimentally Observed	BLCAP

	Human miRecords		
**hsa-miR-1**	TarBase	Experimentally Observed	BRI3BP
**hsa-miR-1**	TarBase miRecords	Experimentally Observed	C12orf49
**hsa-miR-1**	TarBase miRecords	Experimentally Observed	C1orf56
**hsa-miR-1**	TarBase TargetScan Human miRecords	Experimentally Observed	C1orf96
**hsa-miR-1**	miRecords	Experimentally Observed	Calm1
**hsa-miR-1**	TarBase miRecords	Experimentally Observed	CAND1
**hsa-miR-1**	TarBase TargetScan Human miRecords	Experimentally Observed	CAP1
**hsa-miR-1**	TarBase	Experimentally Observed	CDCP1
**hsa-miR-1**	TarBase TargetScan Human miRecords	Experimentally Observed	CDK14
**hsa-miR-1**	TargetScan Human miRecords	Experimentally Observed	CDK9
**hsa-miR-1**	TarBase TargetScan Human miRecords	Experimentally Observed	CERS2
**hsa-miR-1**	TarBase TargetScan Human miRecords	Experimentally Observed	CHST11
**hsa-miR-1**	TarBase TargetScan Human miRecords	Experimentally Observed	CHSY1
**hsa-miR-1**	TarBase TargetScan Human miRecords	Experimentally Observed	CLCN3
**hsa-miR-1**	TargetScan Human miRecords	Experimentally Observed	CNN3
**hsa-miR-1**	TarBase miRecords	Experimentally Observed	CNOT6
**hsa-miR-1**	TarBase TargetScan Human	Experimentally Observed	COIL
**hsa-miR-1**	TarBase TargetScan Human	Experimentally Observed	CORO1C
**hsa-miR-1**	TarBase	Experimentally Observed	CPOX
**hsa-miR-1**	TarBase	Experimentally Observed	CSRP1
**hsa-miR-1**	TarBase	Experimentally Observed	CTSC
**hsa-miR-1**	TarBase TargetScan Human miRecords	Experimentally Observed	DDX5
**hsa-miR-1**	TarBase TargetScan Human miRecords	Experimentally Observed	DHX15
**hsa-miR-1**	TarBase	Experimentally Observed	DNAJB1
**hsa-miR-1**	TarBase	Experimentally Observed	EGFR
**hsa-miR-1**	TarBase	Experimentally Observed	EHMT1
**hsa-miR-1**	TarBase TargetScan Human	Experimentally Observed	EHMT2
**hsa-miR-1**	TarBase TargetScan Human miRecords	Experimentally Observed	EML4
**hsa-miR-1**	TarBase TargetScan Human miRecords	Experimentally Observed	EPB41L4B
**hsa-miR-1**	TarBase miRecords	Experimentally Observed	ESR1

**hsa-miR-1**	TarBase	Experimentally Observed	F2
**hsa-miR-1**	TarBase miRecords	Experimentally Observed	FAM57A
**hsa-miR-1**	TarBase miRecords	Experimentally Observed	FAM81A
**hsa-miR-1**	TarBase TargetScan Human miRecords	Experimentally Observed Moderate (predicted)	FBLN2
**hsa-miR-1**	TarBase	Experimentally Observed	FERMT2
**hsa-miR-1**	TargetScan Human miRecords	Experimentally Observed	FOXP1
**hsa-miR-1**	TarBase miRecords	Experimentally Observed	FSTL1
**hsa-miR-1**	TarBase TargetScan Human miRecords	Experimentally Observed	G6PD
**hsa-miR-1**	TarBase TargetScan Human	Experimentally Observed	GAK
**hsa-miR-1**	TarBase miRecords	Experimentally Observed	GCFC2
**hsa-miR-1**	TarBase TargetScan Human miRecords	Experimentally Observed	GCH1
**hsa-miR-1**	TarBase TargetScan Human miRecords	Experimentally Observed	GJA1
**hsa-miR-1**	TarBase TargetScan Human miRecords	Experimentally Observed	GNPDA2
**hsa-miR-1**	TarBase TargetScan Human	Experimentally Observed	GNPNAT1
**hsa-miR-1**	TarBase	Experimentally Observed	GOLGA7
**hsa-miR-1**	Ingenuity Expert Findings TarBase TargetScan Human	Experimentally Observed	GPD2
**hsa-miR-1**	TarBase TargetScan Human miRecords	Experimentally Observed	H3F3A/H3F3 B
**hsa-miR-1**	TarBase TargetScan Human miRecords	Experimentally Observed	HAND2
**hsa-miR-1**	TarBase miRecords	Experimentally Observed	HCN2
**hsa-miR-1**	TarBase miRecords	Experimentally Observed	HCN4
**hsa-miR-1**	TarBase TargetScan Human miRecords	Experimentally Observed	HDAC4
**hsa-miR-1**	TarBase miRecords	Experimentally Observed	HIST1H3A
**hsa-miR-1**	TarBase TargetScan Human miRecords	Experimentally Observed Moderate (predicted)	HPS4
**hsa-miR-1**	TarBase miRecords	Experimentally Observed	HSPA1A/HSP A1B
**hsa-miR-1**	TarBase TargetScan Human miRecords	Experimentally Observed	HSPD1
**hsa-miR-1**	TarBase TargetScan Human miRecords	Experimentally Observed	IFT52
**hsa-miR-1**	TargetScan Human miRecords	Experimentally Observed	IGF1
**hsa-miR-1**	TarBase miRecords	Experimentally Observed	INPP5F
**hsa-miR-1**	TarBase TargetScan	Experimentally Observed	IP6K2

	Human miRecords		
**hsa-miR-1**	TarBase	Experimentally Observed	IQGAP3
**hsa-miR-1**	miRecords	Experimentally Observed	IRX5
**hsa-miR-1**	TarBase miRecords	Experimentally Observed	ISY1
**hsa-miR-1**	TarBase	Experimentally Observed	ITGB4
**hsa-miR-1**	miRecords	Experimentally Observed	KCNE1
**hsa-miR-1**	TarBase TargetScan Human miRecords	Experimentally Observed	KCNJ2
**hsa-miR-1**	TarBase	Experimentally Observed	KCNQ1
**hsa-miR-1**	TarBase miRecords	Experimentally Observed	KIAA1598
**hsa-miR-1**	TarBase TargetScan Human miRecords	Experimentally Observed	KIF2A
**hsa-miR-1**	TarBase TargetScan Human miRecords	Experimentally Observed	KLHDC5
**hsa-miR-1**	TargetScan Human miRecords	Experimentally Observed	LARP4
**hsa-miR-1**	TarBase TargetScan Human miRecords	Experimentally Observed	LASP1
**hsa-miR-1**	TarBase TargetScan Human miRecords	Experimentally Observed	LIN7C
**hsa-miR-1**	TarBase	Experimentally Observed	LRP1
**hsa-miR-1**	TarBase TargetScan Human miRecords	Experimentally Observed	LRRC8A
**hsa-miR-1**	TarBase miRecords	Experimentally Observed	LZTFL1
**hsa-miR-1**	miRecords	Experimentally Observed	MEF2A
**hsa-miR-1**	TarBase TargetScan Human miRecords	Experimentally Observed	MET
**hsa-miR-1**	TarBase miRecords	Experimentally Observed	MGC27345
**hsa-miR-1**	TarBase TargetScan Human miRecords	Experimentally Observed	MMD
**hsa-miR-1**	TarBase	Experimentally Observed	MOV10
**hsa-miR-1**	TarBase	Experimentally Observed	MRC2
**hsa-miR-1**	TarBase	Experimentally Observed	MTHFD2
**hsa-miR-1**	TarBase TargetScan Human miRecords	Experimentally Observed	MTMR12
**hsa-miR-1**	TarBase TargetScan Human miRecords	Experimentally Observed	MTX1
**hsa-miR-1**	TarBase TargetScan Human miRecords	Experimentally Observed	MXD4
**hsa-miR-1**	TarBase TargetScan Human miRecords	Experimentally Observed Moderate (predicted)	NETO2
**hsa-miR-1**	TarBase TargetScan Human	Experimentally Observed Moderate (predicted)	NOTCH2
**hsa-miR-1**	TargetScan Human miRecords	Experimentally Observed	NOTCH3
**hsa-miR-1**	Ingenuity Expert Findings TarBase	Experimentally Observed	NRP1

	TargetScan Human		
**hsa-miR-1**	TarBase TargetScan Human miRecords	Experimentally Observed	OAT
**hsa-miR-1**	TarBase TargetScan Human miRecords	Experimentally Observed	OSBPL7
**hsa-miR-1**	TarBase TargetScan Human miRecords	Experimentally Observed	PDCD4
**hsa-miR-1**	TarBase	Experimentally Observed	PDLIM7
**hsa-miR-1**	TarBase TargetScan Human miRecords	Experimentally Observed	PGM2
**hsa-miR-1**	TarBase TargetScan Human	Experimentally Observed	PICALM
**hsa-miR-1**	TargetScan Human miRecords	Experimentally Observed	PIM1
**hsa-miR-1**	TarBase miRecords	Experimentally Observed	PLEKHB2
**hsa-miR-1**	TarBase miRecords	Experimentally Observed	PLEKHG2
**hsa-miR-1**	TarBase TargetScan Human miRecords	Experimentally Observed	PNP
**hsa-miR-1**	TarBase TargetScan Human miRecords	Experimentally Observed	POGK
**hsa-miR-1**	TarBase TargetScan Human miRecords	Experimentally Observed	POLA1
**hsa-miR-1**	TarBase	Experimentally Observed	POLA2
**hsa-miR-1**	TarBase TargetScan Human miRecords	Experimentally Observed Moderate (predicted)	POLR2K
**hsa-miR-1**	miRecords	Experimentally Observed	POM121
**hsa-miR-1**	TarBase	Experimentally Observed	POM121C
**hsa-miR-1**	TarBase TargetScan Human	Experimentally Observed	PPIB
**hsa-miR-1**	TarBase TargetScan Human miRecords	Experimentally Observed	PREX1
**hsa-miR-1**	TarBase TargetScan Human	Experimentally Observed Moderate (predicted)	PRSS21
**hsa-miR-1**	TarBase TargetScan Human	Experimentally Observed	PTBP1
**hsa-miR-1**	TarBase	Experimentally Observed	PTBP2
**hsa-miR-1**	TarBase TargetScan Human	Experimentally Observed	PTMA
**hsa-miR-1**	TarBase TargetScan Human miRecords	Experimentally Observed	PTPLAD1
**hsa-miR-1**	TarBase	Experimentally Observed	PTPLB
**hsa-miR-1**	TarBase TargetScan Human	Experimentally Observed Moderate (predicted)	PTPRF
**hsa-miR-1**	TarBase	Experimentally Observed	PWP1
**hsa-miR-1**	TarBase miRecords	Experimentally Observed	RAB11FIP2
**hsa-miR-1**	TarBase miRecords	Experimentally Observed	RABGAP1L
**hsa-miR-1**	TarBase miRecords	Experimentally Observed	RABL2A

**hsa-miR-1**	TarBase miRecords	Experimentally Observed	RABL2B
**hsa-miR-1**	TarBase TargetScan Human miRecords	Experimentally Observed Moderate (predicted)	RBM47
**hsa-miR-1**	TarBase	Experimentally Observed	RFT1
**hsa-miR-1**	TarBase TargetScan Human miRecords	Experimentally Observed	RNF138
**hsa-miR-1**	TarBase	Experimentally Observed	SAC3D1
**hsa-miR-1**	TarBase miRecords	Experimentally Observed	SDC4
**hsa-miR-1**	TarBase	Experimentally Observed	SEC23IP
**hsa-miR-1**	Ingenuity Expert Findings TarBase TargetScan Human miRecords	Experimentally Observed	SERP1
**hsa-miR-1**	TarBase miRecords	Experimentally Observed	SERPINB5
**hsa-miR-1**	TarBase	Experimentally Observed	SFXN1
**hsa-miR-1**	TarBase TargetScan Human miRecords	Experimentally Observed Moderate (predicted)	SH2D4A
**hsa-miR-1**	TarBase TargetScan Human	Experimentally Observed	SH3BGRL3
**hsa-miR-1**	TarBase TargetScan Human miRecords	Experimentally Observed	SH3PXD2B
**hsa-miR-1**	TarBase miRecords	Experimentally Observed	SLC16A9
**hsa-miR-1**	TarBase TargetScan Human	Experimentally Observed	SLC25A1
**hsa-miR-1**	TarBase TargetScan Human	Experimentally Observed	SLC25A22
**hsa-miR-1**	TarBase TargetScan Human miRecords	Experimentally Observed	SLC25A30
**hsa-miR-1**	TarBase TargetScan Human miRecords	Experimentally Observed	SLC44A1
**hsa-miR-1**	TarBase	Experimentally Observed	SNX6
**hsa-miR-1**	TarBase TargetScan Human miRecords	Experimentally Observed	SRSF9
**hsa-miR-1**	TarBase TargetScan Human miRecords	Experimentally Observed	SRXN1
**hsa-miR-1**	TarBase	Experimentally Observed	SSNA1
**hsa-miR-1**	TarBase	Experimentally Observed	SYNE1
**hsa-miR-1**	TarBase miRecords	Experimentally Observed	TAC1
**hsa-miR-1**	Ingenuity Expert Findings TarBase TargetScan Human miRecords	Experimentally Observed	TAGLN2
**hsa-miR-1**	TarBase TargetScan Human miRecords	Experimentally Observed Moderate (predicted)	TDP1
**hsa-miR-1**	TarBase TargetScan Human miRecords	Experimentally Observed	TH1L
**hsa-miR-1**	TarBase TargetScan	Experimentally Observed	THBS1

	Human		
**hsa-miR-1**	TarBase TargetScan Human miRecords	Experimentally Observed	TIMP3
**hsa-miR-1**	TarBase TargetScan Human miRecords	Experimentally Observed	TMSB10/TMSB4X
**hsa-miR-1**	TarBase miRecords	Experimentally Observed	TNS4
**hsa-miR-1**	TarBase	Experimentally Observed	TPM1
**hsa-miR-1**	TarBase	Experimentally Observed	TPM2
**hsa-miR-1**	TarBase TargetScan Human	Experimentally Observed	TPM3
**hsa-miR-1**	TarBase TargetScan Human miRecords	Experimentally Observed	TPM4
**hsa-miR-1**	TargetScan Human miRecords	Experimentally Observed	TPPP
**hsa-miR-1**	TarBase TargetScan Human miRecords	Experimentally Observed	TRAPPC3
**hsa-miR-1**	TarBase TargetScan Human miRecords	Experimentally Observed	TRIM2
**hsa-miR-1**	TarBase TargetScan Human miRecords	Experimentally Observed	TSPAN4
**hsa-miR-1**	TarBase TargetScan Human miRecords	Experimentally Observed	TWF1
**hsa-miR-1**	TarBase TargetScan Human miRecords	Experimentally Observed	UHMK1
**hsa-miR-1**	TarBase	Experimentally Observed	UHRF1
**hsa-miR-1**	TarBase	Experimentally Observed	UNC93B1
**hsa-miR-1**	TarBase TargetScan Human miRecords	Experimentally Observed	UST
**hsa-miR-1**	TarBase TargetScan Human miRecords	Experimentally Observed	UTRN
**hsa-miR-1**	TarBase TargetScan Human	Experimentally Observed Moderate (predicted)	WDFY1
**hsa-miR-1**	TarBase	Experimentally Observed	WDR11
**hsa-miR-1**	TarBase TargetScan Human miRecords	Experimentally Observed Moderate (predicted)	XPNPEP3
**hsa-miR-1**	TarBase TargetScan Human miRecords	Experimentally Observed	XPO6
**hsa-miR-1**	TarBase	Experimentally Observed	YWHAQ
**hsa-miR-1**	TarBase TargetScan Human miRecords	Experimentally Observed Moderate (predicted)	ZNF264
**hsa-miR-10b**	TarBase TargetScan Human miRecords	Experimentally Observed	HOXA1
**hsa-miR-10b**	TarBase TargetScan Human miRecords	Experimentally Observed	HOXD10
**hsa-miR-10b**	miRecords	Experimentally Observed	KLF4
**hsa-miR-**	miRecords	Experimentally Observed	USF2

**10b**			
**hsa-miR-128a**	TargetScan Human miRecords	Experimentally Observed	ADORA2B
**hsa-miR-128a**	miRecords	Experimentally Observed	AFF1
**hsa-miR-128a**	TargetScan Human miRecords	Experimentally Observed	BMI1
**hsa-miR-128a**	miRecords	Experimentally Observed	DBI
**hsa-miR-128a**	TargetScan Human miRecords	Experimentally Observed	DCX
**hsa-miR-128a**	TargetScan Human miRecords	Experimentally Observed	E2F3
**hsa-miR-128a**	miRecords	Experimentally Observed	EIF2C1
**hsa-miR-128a**	TargetScan Human miRecords	Experimentally Observed	LDLR
**hsa-miR-128a**	TargetScan Human miRecords	Experimentally Observed	MLL
**hsa-miR-128a**	TargetScan Human miRecords	Experimentally Observed	NTRK3
**hsa-miR-128a**	TargetScan Human miRecords	Experimentally Observed	RELN
**hsa-miR-128a**	TargetScan Human miRecords	Experimentally Observed	SNAP25
**hsa-miR-128a**	Ingenuity Expert Findings TargetScan Human	Experimentally Observed	TGFBR1
**hsa-miR-128a**	TargetScan Human miRecords	Experimentally Observed	TXNIP
**hsa-miR-128a**	Ingenuity Expert Findings TargetScan Human	Experimentally Observed	WEE1
**hsa-miR-133a**	Ingenuity Expert Findings TargetScan Human	Experimentally Observed	BCL2L2
**hsa-miR-133a**	Ingenuity Expert Findings TarBase miRecords	Experimentally Observed	CASP9
**hsa-miR-133a**	miRecords	Experimentally Observed	CDC42
**hsa-miR-133a**	Ingenuity Expert Findings TargetScan Human	Experimentally Observed	CTGF
**hsa-miR-133a**	Ingenuity Expert Findings TargetScan Human miRecords	Experimentally Observed	FSCN1

**hsa-miR-133a**	TarBase miRecords	Experimentally Observed	HCN2
**hsa-miR-133a**	TarBase	Experimentally Observed	HCN4
**hsa-miR-133a**	TargetScan Human miRecords	Experimentally Observed	IGF1R
**hsa-miR-133a**	TarBase	Experimentally Observed	KCNE1
**hsa-miR-133a**	Ingenuity Expert Findings miRecords	Experimentally Observed	KCNH2
**hsa-miR-133a**	Ingenuity Expert Findings miRecords	Experimentally Observed	KCNQ1
**hsa-miR-133a**	Ingenuity Expert Findings miRecords	Experimentally Observed	KLF15
**hsa-miR-133a**	miRecords	Experimentally Observed	KRT7
**hsa-miR-133a**	Ingenuity Expert Findings TargetScan Human	Experimentally Observed	MCL1
**hsa-miR-133a**	miRecords	Experimentally Observed	NFATC4
**hsa-miR-133a**	miRecords	Experimentally Observed	PITX3
**hsa-miR-133a**	TarBase miRecords	Experimentally Observed	PKM
**hsa-miR-133a**	TarBase TargetScan Human miRecords	Experimentally Observed	PTBP2
**hsa-miR-133a**	miRecords	Experimentally Observed	RHOA
**hsa-miR-133a**	Ingenuity Expert Findings miRecords	Experimentally Observed	RUNX2
**hsa-miR-133a**	TarBase miRecords	Experimentally Observed	SRF
**hsa-miR-133a**	Ingenuity Expert Findings TargetScan Human	Experimentally Observed	TAGLN2
**hsa-miR-133a**	TargetScan Human miRecords	Experimentally Observed	WHSC2
**hsa-miR-135b**	miRecords	Experimentally Observed	ALOX5AP
**hsa-miR-135b**	TargetScan Human miRecords	Experimentally Observed	APC
**hsa-miR-135b**	TargetScan Human miRecords	Experimentally Observed	JAK2
**hsa-miR-135b**	Ingenuity Expert Findings TargetScan Human	Experimentally Observed Moderate (predicted)	RUNX2

**hsa-miR-135b**	TargetScan Human miRecords	Experimentally Observed	SMAD5
**hsa-miR-138**	miRecords	Experimentally Observed	ALDH1A2
**hsa-miR-138**	miRecords	Experimentally Observed	KRT19
**hsa-miR-138**	TargetScan Human miRecords	Experimentally Observed	RHOC
**hsa-miR-138**	TargetScan Human miRecords	Experimentally Observed	ROCK2
**hsa-miR-138**	TarBase	Experimentally Observed	SLC45A3
**hsa-miR-138**	TarBase TargetScan Human	Experimentally Observed Moderate (predicted)	TERT
**hsa-miR-138**	miRecords	Experimentally Observed	VCAN
**hsa-miR-139**	TargetScan Human miRecords	Experimentally Observed	FOXO1
**hsa-miR-140**	miRecords	Experimentally Observed	Cxcl12
**hsa-miR-140**	Ingenuity Expert Findings TargetScan Human	Experimentally Observed	EGR2
**hsa-miR-140**	TargetScan Human miRecords	Experimentally Observed	HDAC4
**hsa-miR-140**	TargetScan Human miRecords	Experimentally Observed	IGFBP5
**hsa-miR-140**	miRecords	Experimentally Observed	SMAD3
**hsa-miR-140**	TargetScan Human miRecords	Experimentally Observed	VEGFA
**hsa-miR-144**	miRecords	Experimentally Observed	ENPP6
**hsa-miR-146b**	miRecords	Experimentally Observed	ATOH8
**hsa-miR-146b**	miRecords	Experimentally Observed	BLMH
**hsa-miR-146b**	miRecords	Experimentally Observed	BRCA1
**hsa-miR-146b**	miRecords	Experimentally Observed	CCL8
**hsa-miR-146b**	miRecords	Experimentally Observed	CCNA2
**hsa-miR-146b**	miRecords	Experimentally Observed	CDKN3
**hsa-miR-146b**	Ingenuity Expert Findings TargetScan	Experimentally Observed Moderate (predicted)	CFH

	Human miRecords		
**hsa-miR-146b**	miRecords	Experimentally Observed	COL13A1
**hsa-miR-146b**	miRecords	Experimentally Observed	CXCR4
**hsa-miR-146b**	miRecords	Experimentally Observed	FADD
**hsa-miR-146b**	TargetScan Human miRecords	Experimentally Observed	IRAK1
**hsa-miR-146b**	miRecords	Experimentally Observed	IRAK2
**hsa-miR-146b**	miRecords	Experimentally Observed	IRF5
**hsa-miR-146b**	miRecords	Experimentally Observed	KIF22
**hsa-miR-146b**	miRecords	Experimentally Observed	LTB
**hsa-miR-146b**	miRecords	Experimentally Observed	MCM10
**hsa-miR-146b**	miRecords	Experimentally Observed	MCPH1
**hsa-miR-146b**	miRecords	Experimentally Observed	METTL7A
**hsa-miR-146b**	TargetScan Human miRecords	Experimentally Observed	MMP16
**hsa-miR-146b**	miRecords	Experimentally Observed	MR1
**hsa-miR-146b**	TargetScan Human miRecords	Experimentally Observed	NFIX
**hsa-miR-146b**	miRecords	Experimentally Observed	PA2G4
**hsa-miR-146b**	TargetScan Human miRecords	Experimentally Observed Moderate (predicted)	PBLD
**hsa-miR-146b**	miRecords	Experimentally Observed	PDIK1L
**hsa-miR-146b**	miRecords	Experimentally Observed	PEX11G
**hsa-miR-146b**	miRecords	Experimentally Observed	PLEKHA4
**hsa-miR-146b**	miRecords	Experimentally Observed	POLE2
**hsa-miR-146b**	miRecords	Experimentally Observed	PRR15
**hsa-miR-146b**	miRecords	Experimentally Observed	RAD54L
**hsa-miR-146b**	miRecords	Experimentally Observed	SDCBP2

**hsa-miR-146b**	miRecords	Experimentally Observed	STAT1
**hsa-miR-146b**	TargetScan Human miRecords	Experimentally Observed	TIMELESS
**hsa-miR-146b**	miRecords	Experimentally Observed	TMSB15A
**hsa-miR-146b**	Ingenuity Expert Findings TargetScan Human	Experimentally Observed	TRAF6
**hsa-miR-146b**	miRecords	Experimentally Observed	TRIM14
**hsa-miR-146b**	TargetScan Human miRecords	Experimentally Observed	UHRF1
**hsa-miR-146b**	miRecords	Experimentally Observed	VWCE
**hsa-miR-182**	TargetScan Human miRecords	Experimentally Observed	ADCY6
**hsa-miR-182**	TargetScan Human miRecords	Experimentally Observed	IGF1R
**hsa-miR-182**	TargetScan Human miRecords	Experimentally Observed	MITF
**hsa-miR-182**	TargetScan Human miRecords	Experimentally Observed	RARG
**hsa-miR-183**	miRecords	Experimentally Observed	BTRC
**hsa-miR-183**	TargetScan Human miRecords	Experimentally Observed	FOXO1
**hsa-miR-183**	Ingenuity Expert Findings TargetScan Human	Experimentally Observed	PDCD4
**hsa-miR-183**	TargetScan Human miRecords	Experimentally Observed	SRSF2
**hsa-miR-196a**	Ingenuity Expert Findings miRecords	Experimentally Observed	ANXA1
**hsa-miR-196a**	TarBase TargetScan Human miRecords	Experimentally Observed	HOXA7
**hsa-miR-196a**	Ingenuity Expert Findings TarBase TargetScan Human miRecords	Experimentally Observed	HOXB8
**hsa-miR-196a**	TarBase TargetScan Human miRecords	Experimentally Observed	HOXC8
**hsa-miR-196a**	TarBase miRecords	Experimentally Observed	HOXD8
**hsa-miR-196a**	miRecords	Experimentally Observed	IKBKB
**hsa-miR-**	TargetScan Human	Experimentally Observed	KRT5

**196a**	miRecords	Moderate (predicted)	
**hsa-miR-196a**	miRecords	Experimentally Observed	S100A9
**hsa-miR-196a**	miRecords	Experimentally Observed	SPRR2C
**hsa-miR-19a**	TargetScan Human miRecords	Experimentally Observed	BCL2L11
**hsa-miR-19a**	TargetScan Human miRecords	Experimentally Observed	BMPR2
**hsa-miR-19a**	TargetScan Human miRecords	Experimentally Observed	CCND1
**hsa-miR-19a**	TargetScan Human miRecords	Experimentally Observed	CTGF
**hsa-miR-19a**	TargetScan Human miRecords	Experimentally Observed	ERBB4
**hsa-miR-19a**	TargetScan Human miRecords	Experimentally Observed	ESR1
**hsa-miR-19a**	TargetScan Human miRecords	Experimentally Observed	HIPK3
**hsa-miR-19a**	TargetScan Human miRecords	Experimentally Observed	MYLIP
**hsa-miR-19a**	miRecords	Experimentally Observed	NR4A2
**hsa-miR-19a**	Ingenuity Expert Findings TarBase TargetScan Human miRecords	Experimentally Observed	PTEN
**hsa-miR-19a**	TargetScan Human miRecords	Experimentally Observed	THBS1
**hsa-miR-200a**	TargetScan Human miRecords	Experimentally Observed	BAP1
**hsa-miR-200a**	TarBase TargetScan Human miRecords	Experimentally Observed	CLOCK
**hsa-miR-200a**	Ingenuity Expert Findings TargetScan Human	Experimentally Observed	CTBP2
**hsa-miR-200a**	miRecords	Experimentally Observed	CTNNB1
**hsa-miR-200a**	miRecords	Experimentally Observed	DLX5
**hsa-miR-200a**	miRecords	Experimentally Observed	ELMO2
**hsa-miR-200a**	Ingenuity Expert Findings TargetScan Human	Experimentally Observed	EPHA2
**hsa-miR-200a**	miRecords	Experimentally Observed	ERBB2IP

**hsa-miR-200a**	Ingenuity Expert Findings TargetScan Human	Experimentally Observed	EXOC5
**hsa-miR-200a**	TarBase	Experimentally Observed	GEMIN2
**hsa-miR-200a**	Ingenuity Expert Findings TargetScan Human	Experimentally Observed	KLF12
**hsa-miR-200a**	miRecords	Experimentally Observed	KLHL20
**hsa-miR-200a**	TargetScan Human miRecords	Experimentally Observed	MAP2K4
**hsa-miR-200a**	Ingenuity Expert Findings TargetScan Human	Experimentally Observed	PPM1E
**hsa-miR-200a**	Ingenuity Expert Findings TargetScan Human	Experimentally Observed	PRKACB
**hsa-miR-200a**	TargetScan Human miRecords	Experimentally Observed	PTPRD
**hsa-miR-200a**	Ingenuity Expert Findings TargetScan Human	Experimentally Observed	STAT5B
**hsa-miR-200a**	TargetScan Human miRecords	Experimentally Observed	TGFB2
**hsa-miR-200a**	miRecords	Experimentally Observed	WDR37
**hsa-miR-200a**	TarBase TargetScan Human miRecords	Experimentally Observed	ZEB1
**hsa-miR-200a**	Ingenuity Expert Findings TarBase TargetScan Human miRecords	Experimentally Observed	ZEB2
**hsa-miR-200a**	TargetScan Human miRecords	Experimentally Observed Moderate (predicted)	ZFPM2
**hsa-miR-210**	TargetScan Human miRecords	Experimentally Observed Moderate (predicted)	ACVR1B
**hsa-miR-210**	miRecords	Experimentally Observed	CASP8AP2
**hsa-miR-210**	TarBase TargetScan Human miRecords	Experimentally Observed	EFNA3
**hsa-miR-210**	miRecords	Experimentally Observed	MNT
**hsa-miR-210**	miRecords	Experimentally Observed	NPTX1
**hsa-miR-210**	Ingenuity Expert Findings TargetScan	Experimentally Observed Moderate (predicted)	SDHD

	Human		
**hsa-miR-204**	miRecords	Experimentally Observed	ARPC1B
**hsa-miR-204**	TargetScan Human miRecords	Experimentally Observed	ATP2B1
**hsa-miR-204**	miRecords	Experimentally Observed	AURKB
**hsa-miR-204**	miRecords	Experimentally Observed	BMP1
**hsa-miR-204**	TargetScan Human miRecords	Experimentally Observed	CDC25B
**hsa-miR-204**	miRecords	Experimentally Observed	CDH11
**hsa-miR-204**	miRecords	Experimentally Observed	CTSC
**hsa-miR-204**	miRecords	Experimentally Observed	EFNB1
**hsa-miR-204**	miRecords	Experimentally Observed	ERF
**hsa-miR-204**	TargetScan Human miRecords	Experimentally Observed	FBN2
**hsa-miR-204**	TargetScan Human miRecords	Experimentally Observed	HMGA2
**hsa-miR-204**	miRecords	Experimentally Observed	HOXB7
**hsa-miR-204**	miRecords	Experimentally Observed	ITGB4
**hsa-miR-204**	miRecords	Experimentally Observed	MMP3
**hsa-miR-204**	miRecords	Experimentally Observed	MMP9
**hsa-miR-204**	TargetScan Human miRecords	Experimentally Observed	SHC1
**hsa-miR-204**	TargetScan Human miRecords	Experimentally Observed	SOX4
**hsa-miR-204**	TargetScan Human miRecords	Experimentally Observed	SPARC
**hsa-miR-204**	miRecords	Experimentally Observed	SPDEF
**hsa-miR-204**	Ingenuity Expert Findings TargetScan Human	Experimentally Observed	TRPS1
**hsa-miR-218**	TargetScan Human miRecords	Experimentally Observed	COL1A1
**hsa-miR-218**	miRecords	Experimentally Observed	KCNJ16
**hsa-miR-**	TargetScan Human	Experimentally Observed	LAMB3

**218**	miRecords	Moderate (predicted)	
**hsa-miR-218**	TargetScan Human miRecords	Experimentally Observed	ONECUT2
**hsa-miR-218**	Ingenuity Expert Findings TargetScan Human	Experimentally Observed	RICTOR
**hsa-miR-218**	Ingenuity Expert Findings TargetScan Human	Experimentally Observed	RUNX2
**hsa-miR-218**	TargetScan Human miRecords	Experimentally Observed	SP1
**hsa-miR-218**	TargetScan Human miRecords	Experimentally Observed	VOPP1
**hsa-miR-224**	TargetScan Human miRecords	Experimentally Observed	AP2M1
**hsa-miR-224**	Ingenuity Expert Findings TarBase TargetScan Human miRecords	Experimentally Observed Moderate (predicted)	API5
**hsa-miR-224**	miRecords	Experimentally Observed	KLK1
**hsa-miR-224**	miRecords	Experimentally Observed	KLK10
**hsa-miR-296**	miRecords	Experimentally Observed	ABCB1
**hsa-miR-296**	miRecords	Experimentally Observed	BAX
**hsa-miR-296**	TargetScan Human miRecords	Experimentally Observed Moderate (predicted)	BCL2
**hsa-miR-296**	TargetScan Human miRecords	Experimentally Observed Moderate (predicted)	CCND1
**hsa-miR-296**	miRecords	Experimentally Observed	CDKN1B
**hsa-miR-296**	miRecords	Experimentally Observed	HGS
**hsa-miR-296**	Ingenuity Expert Findings TargetScan Human	Experimentally Observed	LYPLA2
**hsa-miR-301**	Ingenuity Expert Findings TargetScan Human	Experimentally Observed	ATG2B
**hsa-miR-301**	TarBase TargetScan Human miRecords	Experimentally Observed Moderate (predicted)	CSF1
**hsa-miR-301**	Ingenuity Expert Findings TargetScan Human	Experimentally Observed	DICER1
**hsa-miR-**	TarBase TargetScan	Experimentally Observed	HOXA5

**301**	Human miRecords		
**hsa-miR-301**	TarBase TargetScan Human miRecords	Experimentally Observed	MAFB
**hsa-miR-301**	TarBase TargetScan Human miRecords	Experimentally Observed	MEOX2
**hsa-miR-301**	TarBase	Experimentally Observed	TAC1
**hsa-miR-301**	TargetScan Human miRecords	Experimentally Observed	ZFPM2
**hsa-miR-302d**	TarBase TargetScan Human miRecords	Experimentally Observed	ADAM9
**hsa-miR-302d**	TarBase TargetScan Human miRecords	Experimentally Observed	ANKRD13B
**hsa-miR-302d**	TarBase TargetScan Human miRecords	Experimentally Observed	ANKRD52
**hsa-miR-302d**	TargetScan Human miRecords	Experimentally Observed	APP
**hsa-miR-302d**	TarBase TargetScan Human miRecords	Experimentally Observed	ARHGEF3
**hsa-miR-302d**	TarBase miRecords	Experimentally Observed	BAZ1A
**hsa-miR-302d**	TarBase miRecords	Experimentally Observed	C12orf23
**hsa-miR-302d**	TarBase miRecords	Experimentally Observed	C2orf18
**hsa-miR-302d**	TarBase miRecords	Experimentally Observed	C9orf78
**hsa-miR-302d**	TargetScan Human miRecords	Experimentally Observed	CCND1
**hsa-miR-302d**	TargetScan Human miRecords	Experimentally Observed	CCND2
**hsa-miR-302d**	TarBase miRecords	Experimentally Observed	CD24
**hsa-miR-302d**	TargetScan Human miRecords	Experimentally Observed	CD44
**hsa-miR-302d**	TarBase miRecords	Experimentally Observed	CD83
**hsa-miR-302d**	TarBase	Experimentally Observed	CDK11A/CDK11B
**hsa-miR-302d**	TargetScan Human miRecords	Experimentally Observed	CDK19
**hsa-miR-302d**	TarBase	Experimentally Observed	CDKN1A
**hsa-miR-302d**	TarBase miRecords	Experimentally Observed	CENPF
**hsa-miR-302d**	TarBase TargetScan Human miRecords	Experimentally Observed	CFL2

**hsa-miR-302d**	TarBase miRecords	Experimentally Observed	CMTM4
**hsa-miR-302d**	TarBase TargetScan Human miRecords	Experimentally Observed	CNOT6
**hsa-miR-302d**	TarBase TargetScan Human miRecords	Experimentally Observed Moderate (predicted)	CYB5R4
**hsa-miR-302d**	TargetScan Human miRecords	Experimentally Observed	DKK1
**hsa-miR-302d**	TargetScan Human miRecords	Experimentally Observed	ERBB4
**hsa-miR-302d**	TargetScan Human miRecords	Experimentally Observed	ESR1
**hsa-miR-302d**	TarBase TargetScan Human miRecords	Experimentally Observed Moderate (predicted)	FAM13B
**hsa-miR-302d**	TarBase miRecords	Experimentally Observed	FITM2
**hsa-miR-302d**	TarBase TargetScan Human miRecords	Experimentally Observed	FYCO1
**hsa-miR-302d**	TarBase miRecords	Experimentally Observed	GBAS
**hsa-miR-302d**	TarBase miRecords	Experimentally Observed	GBP3
**hsa-miR-302d**	TarBase miRecords	Experimentally Observed	GLTP
**hsa-miR-302d**	TarBase miRecords	Experimentally Observed	GPSM2
**hsa-miR-302d**	TarBase miRecords	Experimentally Observed	HERPUD1
**hsa-miR-302d**	TarBase miRecords	Experimentally Observed	HSPA14
**hsa-miR-302d**	TarBase miRecords	Experimentally Observed	INSIG2
**hsa-miR-302d**	TarBase TargetScan Human miRecords	Experimentally Observed	KDM1B
**hsa-miR-302d**	TarBase miRecords	Experimentally Observed	KIAA1919
**hsa-miR-302d**	TarBase miRecords	Experimentally Observed	KIF23
**hsa-miR-302d**	TargetScan Human miRecords	Experimentally Observed	KLF13
**hsa-miR-302d**	TarBase miRecords	Experimentally Observed	KLHL12
**hsa-miR-302d**	TarBase TargetScan Human miRecords	Experimentally Observed	LATS2
**hsa-miR-302d**	TargetScan Human miRecords	Experimentally Observed	LEFTY1
**hsa-miR-**	TargetScan Human	Experimentally Observed	LEFTY2

**302d**	miRecords		
**hsa-miR-302d**	TarBase miRecords	Experimentally Observed	LMNB1
**hsa-miR-302d**	TarBase TargetScan Human miRecords	Experimentally Observed	LUC7L2
**hsa-miR-302d**	TargetScan Human miRecords	Experimentally Observed	MBNL2
**hsa-miR-302d**	TarBase miRecords	Experimentally Observed	MED28
**hsa-miR-302d**	TarBase TargetScan Human miRecords	Experimentally Observed	MICA
**hsa-miR-302d**	TarBase TargetScan Human miRecords	Experimentally Observed	MKRN1
**hsa-miR-302d**	TarBase TargetScan Human miRecords	Experimentally Observed	MYBL1
**hsa-miR-302d**	TarBase miRecords	Experimentally Observed	NIN
**hsa-miR-302d**	TargetScan Human miRecords	Experimentally Observed	NR4A2
**hsa-miR-302d**	TarBase miRecords	Experimentally Observed	NUPL1
**hsa-miR-302d**	TarBase miRecords	Experimentally Observed	PACRGL
**hsa-miR-302d**	TarBase TargetScan Human miRecords	Experimentally Observed	PBK
**hsa-miR-302d**	TarBase TargetScan Human miRecords	Experimentally Observed	PCGF5
**hsa-miR-302d**	TarBase TargetScan Human miRecords	Experimentally Observed	PDIK1L
**hsa-miR-302d**	TarBase miRecords	Experimentally Observed	PHC2
**hsa-miR-302d**	TarBase miRecords	Experimentally Observed	PRC1
**hsa-miR-302d**	Ingenuity Expert Findings TargetScan Human	Experimentally Observed	PRKACB
**hsa-miR-302d**	TargetScan Human miRecords	Experimentally Observed	RECK
**hsa-miR-302d**	TarBase TargetScan Human	Experimentally Observed	RELA
**hsa-miR-302d**	TarBase TargetScan Human miRecords	Experimentally Observed Moderate (predicted)	RNF149
**hsa-miR-302d**	TarBase miRecords	Experimentally Observed	RPIA
**hsa-miR-302d**	TarBase miRecords	Experimentally Observed	SLC25A23
**hsa-miR-**	TarBase miRecords	Experimentally Observed	STK4

**302d**			
**hsa-miR-302d**	TarBase miRecords	Experimentally Observed	STX11
**hsa-miR-302d**	TarBase TargetScan Human miRecords	Experimentally Observed Moderate (predicted)	TBC1D2
**hsa-miR-302d**	TarBase miRecords	Experimentally Observed	TEX30
**hsa-miR-302d**	TarBase TargetScan Human miRecords	Experimentally Observed	TFAP4
**hsa-miR-302d**	TarBase miRecords	Experimentally Observed	TMEM14A
**hsa-miR-302d**	TarBase miRecords	Experimentally Observed	TMEM9B
**hsa-miR-302d**	TarBase TargetScan Human miRecords	Experimentally Observed	TNFAIP1
**hsa-miR-302d**	TargetScan Human miRecords	Experimentally Observed	TP63
**hsa-miR-302d**	TargetScan Human miRecords	Experimentally Observed	TRPS1
**hsa-miR-302d**	TarBase miRecords	Experimentally Observed	TTC8
**hsa-miR-302d**	TarBase TargetScan Human miRecords	Experimentally Observed	TUSC2
**hsa-miR-302d**	TarBase miRecords	Experimentally Observed	UBXN1
**hsa-miR-302d**	TarBase miRecords	Experimentally Observed	USP12
**hsa-miR-302d**	TargetScan Human miRecords	Experimentally Observed	VEGFA
**hsa-miR-302d**	TarBase miRecords	Experimentally Observed	VPS26A
**hsa-miR-302d**	TarBase miRecords	Experimentally Observed	ZHX1
**hsa-miR-302d**	TarBase miRecords	Experimentally Observed	ZNF226
**hsa-miR-30a-3p**	miRecords	Experimentally Observed	AQP4
**hsa-miR-30a-3p**	TarBase miRecords	Experimentally Observed	CDK6
**hsa-miR-30a-3p**	TarBase miRecords	Experimentally Observed	CYR61
**hsa-miR-30a-3p**	miRecords	Experimentally Observed	FMR1
**hsa-miR-30a-3p**	TarBase miRecords	Experimentally Observed	SLC7A6
**hsa-miR-30a-3p**	TarBase miRecords	Experimentally Observed	THBS1

**hsa-miR-30a-3p**	TarBase miRecords	Experimentally Observed	TMEM2
**hsa-miR-30a-3p**	TarBase miRecords	Experimentally Observed	TUBA1A
**hsa-miR-30a-3p**	TarBase miRecords	Experimentally Observed	VEZT
**hsa-miR-30a-3p**	TarBase miRecords	Experimentally Observed	WDR82
**hsa-miR-30a-5p**	TargetScan Human miRecords	Experimentally Observed	ACVR1
**hsa-miR-30a-5p**	TarBase	Experimentally Observed	ADPGK
**hsa-miR-30a-5p**	TarBase	Experimentally Observed	ANPEP
**hsa-miR-30a-5p**	TarBase TargetScan Human	Experimentally Observed	AP2A1
**hsa-miR-30a-5p**	TarBase TargetScan Human	Experimentally Observed	ATP2A2
**hsa-miR-30a-5p**	TarBase	Experimentally Observed	ATRX
**hsa-miR-30a-5p**	Ingenuity Expert Findings TargetScan Human	Experimentally Observed	BCL6
**hsa-miR-30a-5p**	TargetScan Human miRecords	Experimentally Observed	BECN1
**hsa-miR-30a-5p**	TarBase	Experimentally Observed	C1orf56
**hsa-miR-30a-5p**	TarBase TargetScan Human	Experimentally Observed	CBFB
**hsa-miR-30a-5p**	TarBase	Experimentally Observed	CDCP1
**hsa-miR-30a-5p**	TarBase	Experimentally Observed	CEP72
**hsa-miR-30a-5p**	TarBase TargetScan Human	Experimentally Observed	CHD1
**hsa-miR-30a-5p**	TarBase TargetScan Human	Experimentally Observed	CPNE8
**hsa-miR-30a-5p**	TarBase TargetScan Human	Experimentally Observed	DOCK7
**hsa-miR-30a-5p**	TarBase TargetScan Human	Experimentally Observed	ELMOD2
**hsa-miR-30a-5p**	TarBase	Experimentally Observed	F2
**hsa-miR-30a-5p**	TarBase	Experimentally Observed	FRG1
**hsa-miR-30a-5p**	TarBase	Experimentally Observed	FXR2

**hsa-miR-30a-5p**	TarBase TargetScan Human	Experimentally Observed	GALNT1
**hsa-miR-30a-5p**	TarBase TargetScan Human	Experimentally Observed	GALNT7
**hsa-miR-30a-5p**	TarBase TargetScan Human miRecords	Experimentally Observed	GNAI2
**hsa-miR-30a-5p**	TarBase	Experimentally Observed	GPD2
**hsa-miR-30a-5p**	TarBase	Experimentally Observed	HNRNPM
**hsa-miR-30a-5p**	TarBase TargetScan Human	Experimentally Observed	IDH1
**hsa-miR-30a-5p**	TarBase	Experimentally Observed	IFRD1
**hsa-miR-30a-5p**	TarBase	Experimentally Observed	ITGA2
**hsa-miR-30a-5p**	TarBase	Experimentally Observed	JUN
**hsa-miR-30a-5p**	TarBase	Experimentally Observed	KDELC2
**hsa-miR-30a-5p**	miRecords	Experimentally Observed	KRT7
**hsa-miR-30a-5p**	TarBase	Experimentally Observed	KRT85
**hsa-miR-30a-5p**	TarBase	Experimentally Observed	LMNB2
**hsa-miR-30a-5p**	TarBase TargetScan Human	Experimentally Observed	LRRC8C
**hsa-miR-30a-5p**	TarBase	Experimentally Observed	LTN1
**hsa-miR-30a-5p**	TarBase TargetScan Human	Experimentally Observed	MAT2A
**hsa-miR-30a-5p**	TarBase TargetScan Human	Experimentally Observed	MBNL1
**hsa-miR-30a-5p**	TarBase	Experimentally Observed	MET
**hsa-miR-30a-5p**	TarBase	Experimentally Observed	MLLT1
**hsa-miR-30a-5p**	TarBase	Experimentally Observed	MPDU1
**hsa-miR-30a-5p**	TarBase	Experimentally Observed	MYO10
**hsa-miR-30a-5p**	TarBase TargetScan Human	Experimentally Observed	NAPG
**hsa-miR-30a-5p**	TarBase TargetScan Human	Experimentally Observed Moderate (predicted)	NCEH1
**hsa-miR-**	TarBase	Experimentally Observed	NCL

**30a-5p**			
**hsa-miR-30a-5p**	TargetScan Human miRecords	Experimentally Observed	NEUROD1
**hsa-miR-30a-5p**	TarBase	Experimentally Observed	NPR3
**hsa-miR-30a-5p**	TarBase	Experimentally Observed	NT5C3
**hsa-miR-30a-5p**	TarBase TargetScan Human	Experimentally Observed	NT5E
**hsa-miR-30a-5p**	TarBase	Experimentally Observed	NUCB1
**hsa-miR-30a-5p**	TarBase TargetScan Human	Experimentally Observed	NUFIP2
**hsa-miR-30a-5p**	TarBase TargetScan Human	Experimentally Observed	P4HA2
**hsa-miR-30a-5p**	TarBase TargetScan Human	Experimentally Observed	PAFAH1B2
**hsa-miR-30a-5p**	TarBase	Experimentally Observed	PEX11B
**hsa-miR-30a-5p**	TarBase TargetScan Human	Experimentally Observed	PGM1
**hsa-miR-30a-5p**	TarBase	Experimentally Observed	PNP
**hsa-miR-30a-5p**	TarBase	Experimentally Observed	PPP2R4
**hsa-miR-30a-5p**	TarBase TargetScan Human	Experimentally Observed	PPP3CA
**hsa-miR-30a-5p**	TarBase TargetScan Human	Experimentally Observed	PRPF40A
**hsa-miR-30a-5p**	TarBase TargetScan Human	Experimentally Observed	PTGFRN
**hsa-miR-30a-5p**	TarBase TargetScan Human	Experimentally Observed	PTPRK
**hsa-miR-30a-5p**	TarBase	Experimentally Observed	PTRH1
**hsa-miR-30a-5p**	TarBase TargetScan Human	Experimentally Observed	RAB27B
**hsa-miR-30a-5p**	TarBase TargetScan Human	Experimentally Observed	RAD23B
**hsa-miR-30a-5p**	TarBase	Experimentally Observed	RBMS1
**hsa-miR-30a-5p**	TarBase	Experimentally Observed	RQCD1
**hsa-miR-30a-5p**	Ingenuity Expert Findings TargetScan Human	Experimentally Observed	RUNX2
**hsa-miR-**	TarBase TargetScan	Experimentally Observed	SEC23A

**30a-5p**	Human		
**hsa-miR-30a-5p**	TarBase TargetScan Human	Experimentally Observed	SEC62
**hsa-miR-30a-5p**	TarBase	Experimentally Observed	SLC12A4
**hsa-miR-30a-5p**	TarBase TargetScan Human	Experimentally Observed	SLC38A1
**hsa-miR-30a-5p**	TarBase TargetScan Human	Experimentally Observed	SLC38A2
**hsa-miR-30a-5p**	TarBase	Experimentally Observed	SLC4A10
**hsa-miR-30a-5p**	TarBase TargetScan Human	Experimentally Observed	SLC4A7
**hsa-miR-30a-5p**	TarBase	Experimentally Observed	SLC7A1
**hsa-miR-30a-5p**	TarBase TargetScan Human	Experimentally Observed	SLC7A11
**hsa-miR-30a-5p**	TarBase	Experimentally Observed	SLC9A3R2
**hsa-miR-30a-5p**	TarBase	Experimentally Observed	STRN
**hsa-miR-30a-5p**	miRecords	Experimentally Observed	STX1A
**hsa-miR-30a-5p**	TarBase	Experimentally Observed	STX7
**hsa-miR-30a-5p**	TarBase TargetScan Human	Experimentally Observed	SYPL1
**hsa-miR-30a-5p**	miRecords	Experimentally Observed	SYT4
**hsa-miR-30a-5p**	TarBase	Experimentally Observed	THEM4
**hsa-miR-30a-5p**	TarBase	Experimentally Observed	TMCO1
**hsa-miR-30a-5p**	TarBase	Experimentally Observed	TMED10
**hsa-miR-30a-5p**	TarBase	Experimentally Observed	TMED2
**hsa-miR-30a-5p**	TarBase	Experimentally Observed	TMED3
**hsa-miR-30a-5p**	TarBase	Experimentally Observed	TMED7
**hsa-miR-30a-5p**	TarBase	Experimentally Observed	TMEM41B
**hsa-miR-30a-5p**	TarBase	Experimentally Observed	TMEM59
**hsa-miR-30a-5p**	TarBase TargetScan Human	Experimentally Observed	TMEM87A

**hsa-miR-30a-5p**	TarBase	Experimentally Observed	TNFAIP2
**hsa-miR-30a-5p**	TarBase TargetScan Human	Experimentally Observed Moderate (predicted)	TNFRSF10B
**hsa-miR-30a-5p**	TargetScan Human miRecords	Experimentally Observed	TNRC6A
**hsa-miR-30a-5p**	miRecords	Experimentally Observed	TP53
**hsa-miR-30a-5p**	Ingenuity Expert Findings TargetScan Human	Experimentally Observed	TRPS1
**hsa-miR-30a-5p**	TarBase	Experimentally Observed	UAP1
**hsa-miR-30a-5p**	TargetScan Human miRecords	Experimentally Observed	UBE2I
**hsa-miR-30a-5p**	TarBase	Experimentally Observed	WDR92
**hsa-miR-30a-5p**	TarBase	Experimentally Observed	WNT5A
**hsa-miR-31**	miRecords	Experimentally Observed	CASR
**hsa-miR-31**	miRecords	Experimentally Observed	CDKN2A
**hsa-miR-31**	TargetScan Human miRecords	Experimentally Observed Moderate (predicted)	FOXP3
**hsa-miR-31**	TargetScan Human miRecords	Experimentally Observed	FZD3
**hsa-miR-31**	miRecords	Experimentally Observed	HIF1A
**hsa-miR-31**	miRecords	Experimentally Observed	ITGA5
**hsa-miR-31**	TargetScan Human miRecords	Experimentally Observed	LATS2
**hsa-miR-31**	miRecords	Experimentally Observed	MMP16
**hsa-miR-31**	miRecords	Experimentally Observed	MPRIP
**hsa-miR-31**	miRecords	Experimentally Observed	PDGFB
**hsa-miR-31**	TargetScan Human miRecords	Experimentally Observed	PPP2R2A
**hsa-miR-31**	TargetScan Human miRecords	Experimentally Observed	RDX
**hsa-miR-31**	miRecords	Experimentally Observed	RHOA
**hsa-miR-31**	Ingenuity Expert Findings TargetScan Human	Experimentally Observed	SATB2
**hsa-miR-328**	miRecords	Experimentally Observed	ABCG2
**hsa-miR-328**	miRecords	Experimentally Observed	BACE1
**hsa-miR-328**	miRecords	Experimentally Observed	CD44
**hsa-miR-**	Ingenuity Expert	Experimentally Observed	BCL6

**339**	Findings TargetScan Human		
**hsa-miR-339**	Ingenuity Expert Findings TargetScan Human	Experimentally Observed Moderate (predicted)	GRM3
**hsa-miR-339**	Ingenuity Expert Findings TargetScan Human	Experimentally Observed	HOXA11
**hsa-miR-33**	Ingenuity Expert Findings TargetScan Human miRecords	Experimentally Observed	ABCA1
**hsa-miR-34c**	TargetScan Human miRecords	Experimentally Observed Moderate (predicted)	AXIN2
**hsa-miR-34c**	Ingenuity Expert Findings TargetScan Human miRecords	Experimentally Observed	BCL2
**hsa-miR-34c**	Ingenuity Expert Findings TarBase TargetScan Human miRecords	Experimentally Observed	CCND1
**hsa-miR-34c**	Ingenuity Expert Findings TargetScan Human	Experimentally Observed Moderate (predicted)	CD47
**hsa-miR-34c**	Ingenuity Expert Findings TarBase TargetScan Human miRecords	Experimentally Observed	CDK6
**hsa-miR-34c**	TarBase TargetScan Human miRecords	Experimentally Observed	DLL1
**hsa-miR-34c**	Ingenuity Expert Findings TarBase TargetScan Human miRecords	Experimentally Observed	E2F3
**hsa-miR-34c**	TargetScan Human miRecords	Experimentally Observed	E2F5
**hsa-miR-34c**	Ingenuity Expert Findings TargetScan Human	Experimentally Observed Moderate (predicted)	HDAC1
**hsa-miR-34c**	TargetScan Human miRecords	Experimentally Observed	JAG1
**hsa-miR-34c**	TargetScan Human miRecords	Experimentally Observed	MAP2K1
**hsa-miR-34c**	Ingenuity Expert Findings TargetScan Human miRecords	Experimentally Observed	MET
**hsa-miR-34c**	TargetScan Human miRecords	Experimentally Observed Moderate (predicted)	MYB

**hsa-miR-34c**	miRecords	Experimentally Observed	MYC
**hsa-miR-34c**	TargetScan Human miRecords	Experimentally Observed	MYCN
**hsa-miR-34c**	Ingenuity Expert Findings TarBase TargetScan Human miRecords	Experimentally Observed	NOTCH1
**hsa-miR-34c**	TargetScan Human miRecords	Experimentally Observed	NOTCH2
**hsa-miR-34c**	Ingenuity Expert Findings TargetScan Human miRecords	Experimentally Observed Moderate (predicted)	SIRT1
**hsa-miR-34c**	TargetScan Human miRecords	Experimentally Observed Moderate (predicted)	TAGLN
**hsa-miR-34c**	miRecords	Experimentally Observed	VEGFA
**hsa-miR-34c**	TargetScan Human miRecords	Experimentally Observed Moderate (predicted)	WISP2
**hsa-miR-34c**	TargetScan Human miRecords	Experimentally Observed	WNT1
**hsa-miR-376a***	TarBase miRecords	Experimentally Observed	SLC16A1
**hsa-miR-376a***	TarBase miRecords	Experimentally Observed	SRSF11
**hsa-miR-376a***	TarBase miRecords	Experimentally Observed	TTK
**hsa-miR-487b**	Ingenuity Expert Findings TargetScan Human	Experimentally Observed Moderate (predicted)	CDKN2AIP
**hsa-miR-487b**	Ingenuity Expert Findings TargetScan Human	Experimentally Observed	MAP2K4
**hsa-miR-499**	TargetScan Human miRecords	Experimentally Observed	SOX6
**hsa-miR-503**	TargetScan Human miRecords	Experimentally Observed	CCND1
**hsa-miR-504**	miRecords	Experimentally Observed	VEGFA
**hsa-miR-7**	TargetScan Human miRecords	Experimentally Observed	EGFR
**hsa-miR-7**	TarBase miRecords	Experimentally Observed	FOS
**hsa-miR-7**	TargetScan Human miRecords	Experimentally Observed	IRS1
**hsa-miR-7**	TargetScan Human miRecords	Experimentally Observed	IRS2
**hsa-miR-7**	Ingenuity Expert	Experimentally Observed	PAK1

	Findings TargetScan Human miRecords	Moderate (predicted)	
**hsa-miR-7**	TargetScan Human miRecords	Experimentally Observed	RAF1
**hsa-miR-7**	miRecords	Experimentally Observed	SLC17A7
**hsa-miR-7**	TargetScan Human miRecords	Experimentally Observed Moderate (predicted)	SLC3A2
**hsa-miR-7**	TargetScan Human miRecords	Experimentally Observed	SNCA
**hsa-miR-7**	miRecords	Experimentally Observed	SYNE1
**hsa-miR-9**	TarBase TargetScan Human miRecords	Experimentally Observed	BACE1
**hsa-miR-9**	Ingenuity Expert Findings TargetScan Human miRecords	Experimentally Observed	CDH1
**hsa-miR-9**	miRecords	Experimentally Observed	FGF16
**hsa-miR-9**	TargetScan Human miRecords	Experimentally Observed	FOXG1
**hsa-miR-9**	TargetScan Human miRecords	Experimentally Observed	FOXO1
**hsa-miR-9**	miRecords	Experimentally Observed	NFKB1
**hsa-miR-9**	miRecords	Experimentally Observed	NTRK3
**hsa-miR-9**	TarBase TargetScan Human miRecords	Experimentally Observed	ONECUT2
**hsa-miR-9**	miRecords	Experimentally Observed	PMP22
**hsa-miR-9**	Ingenuity Expert Findings TargetScan Human miRecords	Experimentally Observed	PRDM1
**hsa-miR-9**	TargetScan Human miRecords	Experimentally Observed	REST
**hsa-miR-92b**	Ingenuity Expert Findings TargetScan Human miRecords	Experimentally Observed	BCL2L11
**hsa-miR-92b**	TargetScan Human miRecords	Experimentally Observed	BMPR2
**hsa-miR-92b**	TargetScan Human miRecords	Experimentally Observed	CDKN1C
**hsa-miR-92b**	miRecords	Experimentally Observed	ENPP6
**hsa-miR-92b**	Ingenuity Expert Findings TargetScan Human	Experimentally Observed	FBXW7
**hsa-miR-92b**	TargetScan Human miRecords	Experimentally Observed	HIPK3
**hsa-miR-92b**	Ingenuity Expert Findings TargetScan Human	Experimentally Observed	IKZF1

**hsa-miR-92b**	TargetScan Human miRecords	Experimentally Observed	ITGA5
**hsa-miR-92b**	TargetScan Human miRecords	Experimentally Observed	MAP2K4
**hsa-miR-92b**	TargetScan Human miRecords	Experimentally Observed	MYLIP
**hsa-miR-92b**	Ingenuity Expert Findings TargetScan Human	Experimentally Observed	PTEN
**hsa-miR-92b**	miRecords	Experimentally Observed	VSNL1
**hsa-miR-99a**	miRecords	Experimentally Observed	FGF16
**hsa-miR-99a**	Ingenuity Expert Findings TargetScan Human miRecords	Experimentally Observed	FGFR3
**hsa-miR-99a**	Ingenuity Expert Findings TargetScan Human miRecords	Experimentally Observed	IGF1R
**hsa-miR-99a**	Ingenuity Expert Findings TargetScan Human miRecords	Experimentally Observed	MTOR
**hsa-miR-99a**	miRecords	Experimentally Observed	PLK1
**hsa-miR-99a**	miRecords	Experimentally Observed	RPTOR
