# Supplementary material for: MicroRNA expression profile in head and neck cancer: HOX-cluster embedded microRNA-196a and microRNA-10b dysregulation implicated in cell proliferation
Source: BMC Cancer. 2013 Nov 9;13:533. doi: 10.1186/1471-2407-13-533 (PMC3826519; doi:10.1186/1471-2407-13-533)
Supplement: Additional file 2 — KEGG term enrichment analysis for gene targets of deregulated miRNAs between cancer and cancer-free samples. KEGG term enrichment analysis were performed using DAVID Bioinformatics Resources (http://david.abcc.ncifcrf.gov/home.jsp). [file 1471-2407-13-533-S2.pdf]

Term	Genes	FDR
**hsa05200:Pathways in cancer**	E2F3, PDGFB, MMP9, STAT5B, FGF16, MITF, FOXO1, NFKB1, PTEN, TGFB2, CTNNB1, CDC42, WNT1, ACVR1B, FOS, CDKN2A, CASP9, RHOA, MYC, EGFR, CTBP2, RELA, TP53, FADD, CDK6, STK4, CCND1, HIF1A, JUN, VEGFA, WNT5A, FGFR3, CDH1, TPM3, IGF1R, LAMB3, BCL2, AXIN2, TRAF6, APC, MAP2K1, TGFBR1, MET, SMAD3, ITGA2, IGF1, RAF1, FZD3, STAT1, CDKN1A, CDKN1B, HDAC1, BAX, MTOR, IKBKB	2.53E-15
**hsa05220:Chronic myeloid leukemia**	E2F3, CTBP2, MAP2K1, TGFBR1, RELA, STAT5B, TP53, SMAD3, RAF1, CDK6, NFKB1, TGFB2, ACVR1B, CCND1, CDKN1A, CDKN2A, CDKN1B, HDAC1, SHC1, IKBKB, MYC	4.18E-08
**hsa05210:Colorectal cancer**	EGFR, MAP2K1, TGFBR1, MET, TP53, SMAD3, RAF1, FZD3, CTNNB1, TGFB2, ACVR1B, FOS, IGF1R, CCND1, CASP9, JUN, BAX, BCL2, AXIN2, MYC, APC	3.91E-07
**hsa05212:Pancreatic cancer**	EGFR, E2F3, MAP2K1, RELA, TGFBR1, TP53, SMAD3, RAF1, CDK6, NFKB1, STAT1, TGFB2, CDC42, ACVR1B, CCND1, CDKN2A, CASP9, VEGFA, IKBKB	1.33E-06
**hsa05215:Prostate cancer**	EGFR, E2F3, MAP2K1, PDGFB, RELA, TP53, FOXO1, IGF1, RAF1, NFKB1, PTEN, CTNNB1, IGF1R, CCND1, CDKN1A, CDKN1B, CASP9, BCL2, MTOR, IKBKB	7.92E-06
**hsa05219:Bladder cancer**	[illegible], FGFR3, MAP2K1, MMP9, TP53, RAF1, CDH1, [illegible]KN1A, CDKN2A, VEGFA, THBS1, MYC	1.77E-05
**hsa05218:Melanoma**	EGFR, E2F3, PDGFB, MAP2K1, FGF16, MET, MITF, TP53, IGF1, RAF1, CDK6, CDH1, PTEN, IGF1R, CCND1, CDKN1A, CDKN2A	5.75E-05
**hsa05214:Glioma**	EGFR, E2F3, PDGFB, MAP2K1, TP53, IGF1, RAF1, CDK6, PTEN, IGF1R, CCND1, CDKN1A, CDKN2A, SHC1, MTOR, CALM1	6.97E-05
**hsa04722:Neurotrophin signaling pathway**	IRAK2, IRAK1, IRS2, MAP2K1, RELA, TP53, RAF1, NFKB1, IRS1, NTRK3, CDC42, BDNF, JUN, BAX, BCL2, YWHAQ, RHOA, SHC1, TRAF6, IKBKB, CALM1	4.46E-04
**hsa04110:Cell cycle**	E2F3, E2F5, TP53, SMAD3, TTK, CDK6, WEE1, TGFB2, CDC25B, CDKN1C, CCND1, CDKN1A, CDKN2A, CDKN1B, HDAC1, CCND2, PLK1, YWHAQ, MYC, CCNA2	0.002
**hsa04510:Focal adhesion**	PDGFB, ITGB4, PTEN, CTNNB1, IGF1R, CDC42, LAMB3, BCL2, RHOA, SHC1, PAK1, THBS1, EGFR, MAP2K1, ROCK2, MET, ITGA2, IGF1, RAF1, CCND1, CCND2, ITGA5, JUN, VEGFA, RELN,	0.002

	COL1A1	
**hsa05213:Endometrial cancer**	EGFR, CCND1, CASP9, MAP2K1, TP53, RAF1, CDH1, AXIN2, MYC, PTEN, CTNNB1, APC	0.014
**hsa05222:Small cell lung cancer**	E2F3, RELA, TP53, ITGA2, CDK6, NFKB1, PTEN, CCND1, LAMB3, CDKN1B, CASP9, BCL2, TRAF6, IKBKB, MYC	0.018
**hsa04350:TGF-beta signaling pathway**	E2F5, ROCK2, TGFBR1, SMAD5, BMPR2, SMAD3, LEFTY1, TGFB2, ACVR1B, SP1, LEFTY2, RHOA, THBS1, MYC, ACVR1	0.027
**hsa04310:Wnt signaling pathway**	WNT5A, CTBP2, ROCK2, BTRC, TP53, SMAD3, FZD3, CTNNB1, WNT1, CCND1, DKK1, CCND2, JUN, RHOA, NFATC4, PRKACB, PPP3CA, AXIN2, MYC, APC	0.037
**hsa04115:p53 signaling pathway**	TP53, IGF1, CDK6, PTEN, CCND1, CDKN1A, TNFRSF10B, CDKN2A, CASP9, SERPINB5, CCND2, BAX, THBS1	0.041
**hsa04010:MAPK signaling pathway**	FGFR3, PDGFB, FGF16, HSPA1A, NFKB1, SRF, TGFB2, FOS, ACVR1B, CDC42, BDNF, NFATC4, PRKACB, PAK1, PPP3CA, TRAF6, MYC, EGFR, MAP2K1, RELA, TGFBR1, MAP2K4, TP53, RAF1, STK4, CDC25B, JUN, IKBKB	0.047
