# Supplementary material for: MicroRNA expression profile in head and neck cancer: HOX-cluster embedded microRNA-196a and microRNA-10b dysregulation implicated in cell proliferation
Source: BMC Cancer. 2013 Nov 9;13:533. doi: 10.1186/1471-2407-13-533 (PMC3826519; doi:10.1186/1471-2407-13-533)
Supplement: Additional file 3 — Differentially expressed genes between SCC25 overexpressing miR-10b and transfection controls. [file 1471-2407-13-533-S3.pdf]

Gene Symbol	FoldChange	FoldChange Description	Gene Symbol	FoldChange	FoldChange Description
**ABCA10**	2.34	SCC25 10b down vs Scramble	AAA1	5.19	SCC25 10b up vs Scramble
**ABHD16B**	2.10	SCC25 10b down vs Scramble	AADACL4	2.36	SCC25 10b up vs Scramble
**ADAM9**	1.91	SCC25 10b down vs Scramble	ABCG2	2.10	SCC25 10b up vs Scramble
**ADAMTS12**	4.49	SCC25 10b down vs Scramble	ADRB3	2.11	SCC25 10b up vs Scramble
**ADH1C**	2.62	SCC25 10b down vs Scramble	AFM	4.14	SCC25 10b up vs Scramble
**AHSP**	2.22	SCC25 10b down vs Scramble	AMHR2	2.13	SCC25 10b up vs Scramble
**AOX2P**	2.25	SCC25 10b down vs Scramble	ANKK1	7.45	SCC25 10b up vs Scramble
**APOBEC2**	2.09	SCC25 10b down vs Scramble	AQP9	2.43	SCC25 10b up vs Scramble
**APOBEC3D**	2.36	SCC25 10b down vs Scramble	ASCL1	2.25	SCC25 10b up vs Scramble
**ARID3C**	2.18	SCC25 10b down vs Scramble	ATPBD4	2.79	SCC25 10b up vs Scramble
**ATOH7**	2.53	SCC25 10b down vs Scramble	AVPR2	1.96	SCC25 10b up vs Scramble
**ATP8B2**	2.03	SCC25 10b down vs Scramble	B3GALT2	2.93	SCC25 10b up vs Scramble
**BPIL2**	2.19	SCC25 10b down vs	B3GAT2	3.03	SCC25 10b up vs Scramble

		Scramble			
**BTNL9**	1.96	SCC25 10b down vs Scramble	BBOX1	3.36	SCC25 10b up vs Scramble
**CA7**	2.40	SCC25 10b down vs Scramble	BEND2	2.52	SCC25 10b up vs Scramble
**CACNG8**	4.30	SCC25 10b down vs Scramble	BRSK1	1.94	SCC25 10b up vs Scramble
**CADPS2**	2.00	SCC25 10b down vs Scramble	BTBD17	2.52	SCC25 10b up vs Scramble
**CALCA**	3.87	SCC25 10b down vs Scramble	CCDC36	2.89	SCC25 10b up vs Scramble
**CCDC151**	2.25	SCC25 10b down vs Scramble	CCDC48	2.71	SCC25 10b up vs Scramble
**CCDC155**	1.90	SCC25 10b down vs Scramble	CD300LD	1.99	SCC25 10b up vs Scramble
**CCNB3**	6.76	SCC25 10b down vs Scramble	CD300LG	6.54	SCC25 10b up vs Scramble
**CDH15**	1.96	SCC25 10b down vs Scramble	CDRT7	5.30	SCC25 10b up vs Scramble
**CDHR1**	2.08	SCC25 10b down vs Scramble	CLDN25	2.03	SCC25 10b up vs Scramble
**CHKB**	2.92	SCC25 10b down vs Scramble	CLEC14A	2.64	SCC25 10b up vs Scramble
**CHST9-AS1**	3.34	SCC25 10b down vs Scramble	CPA3	3.13	SCC25 10b up vs Scramble
**CLCNKB**	2.10	SCC25 10b down vs Scramble	CRHR2	2.17	SCC25 10b up vs Scramble

**CLEC1B**	2.44	SCC25 10b down vs Scramble	CTRB1	3.34	SCC25 10b up vs Scramble
**CNGA4**	5.80	SCC25 10b down vs Scramble	CYP4F11	2.85	SCC25 10b up vs Scramble
**CNR1**	16.89	SCC25 10b down vs Scramble	DEFA8P	1.95	SCC25 10b up vs Scramble
**CNTF**	1.90	SCC25 10b down vs Scramble	DEPDC4	2.21	SCC25 10b up vs Scramble
**COL6A5**	2.45	SCC25 10b down vs Scramble	DLX3	2.51	SCC25 10b up vs Scramble
**CPNE9**	2.19	SCC25 10b down vs Scramble	DMRT3	3.94	SCC25 10b up vs Scramble
**CR737729**	2.36	SCC25 10b down vs Scramble	DNM1P35	2.27	SCC25 10b up vs Scramble
**CSMD3**	2.37	SCC25 10b down vs Scramble	FAM49A	1.98	SCC25 10b up vs Scramble
**CST2**	28.44	SCC25 10b down vs Scramble	FAM65B	2.15	SCC25 10b up vs Scramble
**CTXN3**	3.42	SCC25 10b down vs Scramble	FAM90A7	3.15	SCC25 10b up vs Scramble
**CXCL9**	2.01	SCC25 10b down vs Scramble	FBXW10	2.27	SCC25 10b up vs Scramble
**CYLC2**	2.19	SCC25 10b down vs Scramble	FGF17	4.51	SCC25 10b up vs Scramble
**CYP46A1**	1.97	SCC25 10b down vs Scramble	FHDC1	1.91	SCC25 10b up vs Scramble
**DCDC2B**	2.66	SCC25 10b down vs	FLJ42022	2.22	SCC25 10b up vs Scramble

		Scramble			
**DEFA7P**	1.92	SCC25 10b down vs Scramble	FLJ45684	2.73	SCC25 10b up vs Scramble
**DEFB104B**	2.02	SCC25 10b down vs Scramble	FOXJ1	2.25	SCC25 10b up vs Scramble
**DEFB133**	3.52	SCC25 10b down vs Scramble	FP588	2.11	SCC25 10b up vs Scramble
**DKKL1**	2.78	SCC25 10b down vs Scramble	GALR2	3.44	SCC25 10b up vs Scramble
**DNAH1**	2.68	SCC25 10b down vs Scramble	GATA5	2.09	SCC25 10b up vs Scramble
**DNAL1**	2.04	SCC25 10b down vs Scramble	GCET2	2.06	SCC25 10b up vs Scramble
**DRD3**	3.90	SCC25 10b down vs Scramble	GDF10	1.97	SCC25 10b up vs Scramble
**DYDC2**	2.05	SCC25 10b down vs Scramble	GHRLOS2	2.01	SCC25 10b up vs Scramble
**EFNA5**	3.96	SCC25 10b down vs Scramble	GNAT2	2.53	SCC25 10b up vs Scramble
**EGFL6**	3.03	SCC25 10b down vs Scramble	GPR144	2.30	SCC25 10b up vs Scramble
**ESRRG**	2.14	SCC25 10b down vs Scramble	GPR156	2.27	SCC25 10b up vs Scramble
**FAM101A**	2.43	SCC25 10b down vs Scramble	GPR179	5.85	SCC25 10b up vs Scramble
**FAM123A**	2.37	SCC25 10b down vs Scramble	GPR18	4.77	SCC25 10b up vs Scramble

**FAM47E**	2.68	SCC25 10b down vs Scramble	GPR63	1.97	SCC25 10b up vs Scramble
**FCGR2B**	3.08	SCC25 10b down vs Scramble	GRM2	2.01	SCC25 10b up vs Scramble
**FGF14**	1.92	SCC25 10b down vs Scramble	HAL	1.99	SCC25 10b up vs Scramble
**FLJ13744**	2.68	SCC25 10b down vs Scramble	HHIPL2	2.60	SCC25 10b up vs Scramble
**FLJ16124**	4.74	SCC25 10b down vs Scramble	HIST1H2AA	2.45	SCC25 10b up vs Scramble
**FLJ34690**	2.38	SCC25 10b down vs Scramble	HTN1	10.07	SCC25 10b up vs Scramble
**FLJ42969**	3.09	SCC25 10b down vs Scramble	IL12RB1	1.98	SCC25 10b up vs Scramble
**FLJ44511**	2.76	SCC25 10b down vs Scramble	IQCF6	2.18	SCC25 10b up vs Scramble
**FLJ45079**	4.89	SCC25 10b down vs Scramble	JOSD2	2.25	SCC25 10b up vs Scramble
**FLJ45950**	2.55	SCC25 10b down vs Scramble	KCND1	10.06	SCC25 10b up vs Scramble
**FMO1**	2.97	SCC25 10b down vs Scramble	KCNH6	9.19	SCC25 10b up vs Scramble
**FMR1-AS1**	3.79	SCC25 10b down vs Scramble	KIAA0889	1.96	SCC25 10b up vs Scramble
**FN3K**	2.53	SCC25 10b down vs Scramble	KIAA1468	2.07	SCC25 10b up vs Scramble
**FRG2**	6.82	SCC25 10b down vs	KLHL15	2.23	SCC25 10b up vs Scramble

		Scramble			
**FUT10**	3.19	SCC25 10b down vs Scramble	KLHL38	1.95	SCC25 10b up vs Scramble
**FUT5**	1.99	SCC25 10b down vs Scramble	KPNA7	2.01	SCC25 10b up vs Scramble
**GABRA2**	1.94	SCC25 10b down vs Scramble	KRT4	2.09	SCC25 10b up vs Scramble
**GGNBP1**	2.81	SCC25 10b down vs Scramble	KRT78	2.25	SCC25 10b up vs Scramble
**GJB6**	2.90	SCC25 10b down vs Scramble	KY	5.97	SCC25 10b up vs Scramble
**GJC3**	2.19	SCC25 10b down vs Scramble	LDLRAD2	3.48	SCC25 10b up vs Scramble
**GNGT1**	2.11	SCC25 10b down vs Scramble	LILRB2	2.56	SCC25 10b up vs Scramble
**GP1BA**	2.69	SCC25 10b down vs Scramble	LIPG	2.12	SCC25 10b up vs Scramble
**GPR101**	3.34	SCC25 10b down vs Scramble	LRP2	2.29	SCC25 10b up vs Scramble
**GPR62**	58.77	SCC25 10b down vs Scramble	LRRK2	2.12	SCC25 10b up vs Scramble
**HCAR2**	2.50	SCC25 10b down vs Scramble	LYZL1	3.33	SCC25 10b up vs Scramble
**HES5**	3.61	SCC25 10b down vs Scramble	MAGEB17	2.09	SCC25 10b up vs Scramble
**HHATL**	2.17	SCC25 10b down vs Scramble	MRS2P2	9.38	SCC25 10b up vs Scramble

**HIST1H4G**	3.48	SCC25 10b down vs Scramble	MS4A13	2.13	SCC25 10b up vs Scramble
**HNF1B**	2.04	SCC25 10b down vs Scramble	MST152	2.67	SCC25 10b up vs Scramble
**HOTAIR**	1.95	SCC25 10b down vs Scramble	MYH16	3.22	SCC25 10b up vs Scramble
**HOXA2**	2.30	SCC25 10b down vs Scramble	NCAN	2.05	SCC25 10b up vs Scramble
**HPGD**	2.71	SCC25 10b down vs Scramble	NCOA2	2.23	SCC25 10b up vs Scramble
**HTRA4**	2.02	SCC25 10b down vs Scramble	NMNAT2	2.04	SCC25 10b up vs Scramble
**HUNK**	2.29	SCC25 10b down vs Scramble	NPS	8.15	SCC25 10b up vs Scramble
**ICOS**	2.04	SCC25 10b down vs Scramble	NTNG2	4.49	SCC25 10b up vs Scramble
**INE1**	2.07	SCC25 10b down vs Scramble	NXF2	1.93	SCC25 10b up vs Scramble
**IPMK**	2.24	SCC25 10b down vs Scramble	OR14A16	1.99	SCC25 10b up vs Scramble
**IRF2BP2**	2.44	SCC25 10b down vs Scramble	OR1F1	2.78	SCC25 10b up vs Scramble
**KLHDC1**	2.10	SCC25 10b down vs Scramble	OR2A20P	2.19	SCC25 10b up vs Scramble
**KLRD1**	1.99	SCC25 10b down vs Scramble	OR2T33	2.58	SCC25 10b up vs Scramble
**KRT75**	1.96	SCC25 10b down vs	OR51E2	2.27	SCC25 10b up vs Scramble

		Scramble			
**KRTAP23-1**	2.63	SCC25 10b down vs Scramble	OR52E8	2.14	SCC25 10b up vs Scramble
**LCE2B**	2.97	SCC25 10b down vs Scramble	OR56A4	5.04	SCC25 10b up vs Scramble
**LCE6A**	2.07	SCC25 10b down vs Scramble	OR5F1	1.96	SCC25 10b up vs Scramble
**LHX5**	7.82	SCC25 10b down vs Scramble	OR8G1	3.05	SCC25 10b up vs Scramble
**LIMD2**	2.56	SCC25 10b down vs Scramble	PALM2	1.99	SCC25 10b up vs Scramble
**LIPM**	2.39	SCC25 10b down vs Scramble	PARP15	2.90	SCC25 10b up vs Scramble
**LIPN**	2.21	SCC25 10b down vs Scramble	PDE3A	2.45	SCC25 10b up vs Scramble
**LRIT3**	1.96	SCC25 10b down vs Scramble	PEBP4	2.44	SCC25 10b up vs Scramble
**LRRC7**	2.19	SCC25 10b down vs Scramble	PLA2G7	1.98	SCC25 10b up vs Scramble
**MAB21L3**	2.53	SCC25 10b down vs Scramble	PLVAP	2.35	SCC25 10b up vs Scramble
**MARK1**	3.31	SCC25 10b down vs Scramble	PNMA6A	1.94	SCC25 10b up vs Scramble
**MCCD1**	1.94	SCC25 10b down vs Scramble	PP2D1	5.41	SCC25 10b up vs Scramble
**MEGF10**	2.03	SCC25 10b down vs Scramble	PRHOXNB	3.81	SCC25 10b up vs Scramble

**MGAM**	2.68	SCC25 10b down vs Scramble	PRO0611	2.53	SCC25 10b up vs Scramble
**MGC12916**	1.96	SCC25 10b down vs Scramble	PRR5L	1.90	SCC25 10b up vs Scramble
**MS4A6E**	8.72	SCC25 10b down vs Scramble	PYDC2	1.92	SCC25 10b up vs Scramble
**MTCP1**	2.39	SCC25 10b down vs Scramble	RAB14	2.05	SCC25 10b up vs Scramble
**MYL2**	2.12	SCC25 10b down vs Scramble	RABIF	3.56	SCC25 10b up vs Scramble
**MYLK3**	2.21	SCC25 10b down vs Scramble	REG1B	2.13	SCC25 10b up vs Scramble
**MYO7B**	2.31	SCC25 10b down vs Scramble	RESP18	1.92	SCC25 10b up vs Scramble
**NBPF7**	3.91	SCC25 10b down vs Scramble	RIMS1	3.04	SCC25 10b up vs Scramble
**NEU3**	3.56	SCC25 10b down vs Scramble	RNU105B	1.93	SCC25 10b up vs Scramble
**NEUROD2**	2.07	SCC25 10b down vs Scramble	SAMD8	1.95	SCC25 10b up vs Scramble
**NOTO**	2.01	SCC25 10b down vs Scramble	SCN7A	2.89	SCC25 10b up vs Scramble
**NPHS1**	1.96	SCC25 10b down vs Scramble	SEMG2	2.35	SCC25 10b up vs Scramble
**ODF4**	2.08	SCC25 10b down vs Scramble	SGIP1	2.51	SCC25 10b up vs Scramble
**OPRK1**	14.25	SCC25 10b down vs	SGPP2	3.12	SCC25 10b up vs Scramble

		Scramble			
**OR13F1**	4.18	SCC25 10b down vs Scramble	SIPA1L3	2.27	SCC25 10b up vs Scramble
**OR1S1**	2.51	SCC25 10b down vs Scramble	SLC24A2	2.18	SCC25 10b up vs Scramble
**OR2A2**	2.38	SCC25 10b down vs Scramble	SLC29A2	2.43	SCC25 10b up vs Scramble
**OR2J2**	2.19	SCC25 10b down vs Scramble	SLC39A12	3.84	SCC25 10b up vs Scramble
**OR2Z1**	1.92	SCC25 10b down vs Scramble	SLC44A4	2.03	SCC25 10b up vs Scramble
**OR3A1**	9.94	SCC25 10b down vs Scramble	SLC4A8	2.40	SCC25 10b up vs Scramble
**OR4A5**	1.90	SCC25 10b down vs Scramble	SLC5A1	1.93	SCC25 10b up vs Scramble
**OR51D1**	3.31	SCC25 10b down vs Scramble	SLC7A7	2.56	SCC25 10b up vs Scramble
**OR6M1**	2.21	SCC25 10b down vs Scramble	SLC8A1	1.99	SCC25 10b up vs Scramble
**OR6V1**	1.97	SCC25 10b down vs Scramble	SMTNL1	2.46	SCC25 10b up vs Scramble
**OR7A17**	7.83	SCC25 10b down vs Scramble	SPAG8	4.31	SCC25 10b up vs Scramble
**OR8H3**	2.00	SCC25 10b down vs Scramble	SPAM1	5.83	SCC25 10b up vs Scramble
**OR8K5**	7.99	SCC25 10b down vs Scramble	SPINK2	2.82	SCC25 10b up vs Scramble

**OR9Q1**	3.13	SCC25 10b down vs Scramble	SPRR1B	1.95	SCC25 10b up vs Scramble
**P2RX3**	3.19	SCC25 10b down vs Scramble	SPTB	2.46	SCC25 10b up vs Scramble
**P2RY10**	2.16	SCC25 10b down vs Scramble	ST8SIA1	2.34	SCC25 10b up vs Scramble
**PADI1**	239.41	SCC25 10b down vs Scramble	STAB2	2.44	SCC25 10b up vs Scramble
**PAK3**	6.18	SCC25 10b down vs Scramble	SUCNR1	1.93	SCC25 10b up vs Scramble
**PATE4**	5.03	SCC25 10b down vs Scramble	SV2C	2.25	SCC25 10b up vs Scramble
**PBX1**	2.30	SCC25 10b down vs Scramble	TEX13B	4.64	SCC25 10b up vs Scramble
**PCDH9**	2.06	SCC25 10b down vs Scramble	TFCP2L1	2.05	SCC25 10b up vs Scramble
**PCDHGB2**	2.73	SCC25 10b down vs Scramble	THEM5	2.11	SCC25 10b up vs Scramble
**PDILT**	2.36	SCC25 10b down vs Scramble	TMED11P	4.21	SCC25 10b up vs Scramble
**PECAM1**	2.08	SCC25 10b down vs Scramble	TMEM174	2.62	SCC25 10b up vs Scramble
**PIK3C2G**	2.29	SCC25 10b down vs Scramble	TMEM211	2.42	SCC25 10b up vs Scramble
**PLA2G2C**	2.13	SCC25 10b down vs Scramble	TMEM35	2.38	SCC25 10b up vs Scramble
**PLA2G2D**	4.26	SCC25 10b down vs	TMOD2	2.04	SCC25 10b up vs Scramble

		Scramble			
**PLA2G2F**	2.47	SCC25 10b down vs Scramble	TMSB4Y	2.36	SCC25 10b up vs Scramble
**PLCXD3**	2.12	SCC25 10b down vs Scramble	TNFRSF9	24.07	SCC25 10b up vs Scramble
**PLD5**	2.16	SCC25 10b down vs Scramble	TPD52L3	6.25	SCC25 10b up vs Scramble
**PLUNC**	2.19	SCC25 10b down vs Scramble	TRPC2	1.95	SCC25 10b up vs Scramble
**POU5F2**	4.74	SCC25 10b down vs Scramble	TTTY6	2.13	SCC25 10b up vs Scramble
**PROX2**	2.40	SCC25 10b down vs Scramble	TUBA3D	2.24	SCC25 10b up vs Scramble
**PRSS57**	2.05	SCC25 10b down vs Scramble	TULP1	2.46	SCC25 10b up vs Scramble
**PSORS1C2**	2.09	SCC25 10b down vs Scramble	UBE2DNL	1.95	SCC25 10b up vs Scramble
**RAB33B**	7.61	SCC25 10b down vs Scramble	UGT8	21.52	SCC25 10b up vs Scramble
**RGR**	2.42	SCC25 10b down vs Scramble	UPP2	2.21	SCC25 10b up vs Scramble
**RHOF**	7.02	SCC25 10b down vs Scramble	WDR72	10.90	SCC25 10b up vs Scramble
**RNASE8**	2.80	SCC25 10b down vs Scramble	WFDC5	2.15	SCC25 10b up vs Scramble
**RPE65**	6.12	SCC25 10b down vs Scramble	X57723	2.33	SCC25 10b up vs Scramble

**RPS6KL1**	1.97	SCC25 10b down vs Scramble	ZBTB38	2.64	SCC25 10b up vs Scramble
**RRN3P2**	2.63	SCC25 10b down vs Scramble	ZDHHC22	2.01	SCC25 10b up vs Scramble
**RTL1**	3.03	SCC25 10b down vs Scramble	ZNF227	2.23	SCC25 10b up vs Scramble
**SCARNA16**	2.01	SCC25 10b down vs Scramble	ZNF804B	8.76	SCC25 10b up vs Scramble
**SDK2**	2.50	SCC25 10b down vs Scramble	ZP4	2.21	SCC25 10b up vs Scramble
**SEZ6L**	4.09	SCC25 10b down vs Scramble			
**SIGLECP3**	1.93	SCC25 10b down vs Scramble			
**SLC17A2**	2.01	SCC25 10b down vs Scramble			
**SLC17A3**	2.17	SCC25 10b down vs Scramble			
**SLC22A9**	3.04	SCC25 10b down vs Scramble			
**SLC5A7**	2.03	SCC25 10b down vs Scramble			
**SLC5A9**	2.61	SCC25 10b down vs Scramble			
**SLC6A12**	2.63	SCC25 10b down vs Scramble			
**SLC9A3**	11.77	SCC25 10b down vs			

		Scramble
**SLFN12**	7.40	SCC25 10b down vs Scramble
**SNORA26**	2.86	SCC25 10b down vs Scramble
**SNX20**	38.82	SCC25 10b down vs Scramble
**SPATA19**	2.77	SCC25 10b down vs Scramble
**SPINK4**	4.28	SCC25 10b down vs Scramble
**SSPO**	7.86	SCC25 10b down vs Scramble
**SSTR2**	2.72	SCC25 10b down vs Scramble
**STK32A**	1.97	SCC25 10b down vs Scramble
**STON2**	2.17	SCC25 10b down vs Scramble
**SUN3**	2.10	SCC25 10b down vs Scramble
**SUSD5**	2.48	SCC25 10b down vs Scramble
**SYCP3**	1.90	SCC25 10b down vs Scramble
**TACR1**	3.06	SCC25 10b down vs Scramble

**TAGLN3**	2.12	SCC25 10b down vs Scramble
**TCF24**	1.97	SCC25 10b down vs Scramble
**TEX28**	2.01	SCC25 10b down vs Scramble
**TGIF2LY**	4.69	SCC25 10b down vs Scramble
**TM4SF4**	6.43	SCC25 10b down vs Scramble
**TMEM235**	2.37	SCC25 10b down vs Scramble
**TP63**	2.57	SCC25 10b down vs Scramble
**TRIM40**	10.71	SCC25 10b down vs Scramble
**TRPV6**	4.40	SCC25 10b down vs Scramble
**TRUB1**	3.14	SCC25 10b down vs Scramble
**TSSK2**	3.45	SCC25 10b down vs Scramble
**TTLL10**	2.78	SCC25 10b down vs Scramble
**TTLL13**	2.11	SCC25 10b down vs Scramble
**TTTY4C**	5.93	SCC25 10b down vs

		Scramble
**VGLL2**	2.53	SCC25 10b down vs Scramble
**WISP1**	3.09	SCC25 10b down vs Scramble
**WNT2B**	1.97	SCC25 10b down vs Scramble
**XKRX**	2.02	SCC25 10b down vs Scramble
**XPNPEP3**	2.05	SCC25 10b down vs Scramble
**ZAR1L**	2.40	SCC25 10b down vs Scramble
**ZNF215**	7.58	SCC25 10b down vs Scramble
**ZNF833P**	1.99	SCC25 10b down vs Scramble
