# Supplementary material for: MicroRNA expression profile in head and neck cancer: HOX-cluster embedded microRNA-196a and microRNA-10b dysregulation implicated in cell proliferation
Source: BMC Cancer. 2013 Nov 9;13:533. doi: 10.1186/1471-2407-13-533 (PMC3826519; doi:10.1186/1471-2407-13-533)
Supplement: Additional file 4 — Differentially expressed genes between FaDu overexpressing miR-10b and transfection controls. [file 1471-2407-13-533-S4.pdf]

Gene Symbol	FoldChange	FoldChange Description	Gene Symbol	FoldChange	FoldChange Description
**ABCA10**	1.94	FaDu 10b down vs FaDu Control	ACMSD	3.11	FaDu 10b up vs FaDu Control
**ACTN2**	2.15	FaDu 10b down vs FaDu Control	ADCY2	2.44	FaDu 10b up vs FaDu Control
**ADAMTS12**	3.41	FaDu 10b down vs FaDu Control	ADCYAP1	2.67	FaDu 10b up vs FaDu Control
**ADRA2B**	2.07	FaDu 10b down vs FaDu Control	ANKRD6	2.06	FaDu 10b up vs FaDu Control
**AGBL3**	3.37	FaDu 10b down vs FaDu Control	AOX2P	2.21	FaDu 10b up vs FaDu Control
**AGMO**	2.28	FaDu 10b down vs FaDu Control	ARHGEF38	1.93	FaDu 10b up vs FaDu Control
**AK022341**	1.95	FaDu 10b down vs FaDu Control	ASIP	5.80	FaDu 10b up vs FaDu Control
**AK095699**	22.75	FaDu 10b down vs FaDu Control	ATP6V0D2	1.98	FaDu 10b up vs FaDu Control
**AK123491**	2.57	FaDu 10b down vs FaDu Control	CACNA2D3	2.31	FaDu 10b up vs FaDu Control
**AKR1CL1**	2.51	FaDu 10b down vs FaDu Control	CD675678	2.01	FaDu 10b up vs FaDu Control
**AL582488**	2.21	FaDu 10b down vs FaDu Control	CHIC1	2.05	FaDu 10b up vs FaDu Control
**ALOX15B**	2.05	FaDu 10b down vs FaDu Control	CHRNA1	1.95	FaDu 10b up vs FaDu Control
**ANKK1**	2.06	FaDu 10b down vs FaDu Control	CHRNA2	2.97	FaDu 10b up vs FaDu Control
**ANKRD33B**	2.01	FaDu 10b down vs FaDu Control	CKM	2.29	FaDu 10b up vs FaDu Control
**ANO1**	8.65	FaDu 10b down vs FaDu Control	CKMT2	2.06	FaDu 10b up vs FaDu Control
**APOBEC3D**	3.85	FaDu 10b down vs FaDu Control	CLC	2.63	FaDu 10b up vs FaDu Control
**ARMC2**	2.17	FaDu 10b down vs FaDu Control	CLEC4G	1.91	FaDu 10b up vs FaDu Control
**ASB11**	2.50	FaDu 10b down vs FaDu Control	CLPS	2.23	FaDu 10b up vs FaDu Control
**ASB18**	7.98	FaDu 10b down vs FaDu Control	CPA3	1.97	FaDu 10b up vs FaDu Control
**ATP12A**	2.13	FaDu 10b down vs FaDu Control	CPNE9	1.96	FaDu 10b up vs FaDu Control
**ATP1B2**	2.35	FaDu 10b down vs FaDu Control	CPS1-IT1	2.29	FaDu 10b up vs FaDu Control
**ATP2B1**	3.73	FaDu 10b down vs FaDu Control	CR1L	2.27	FaDu 10b up vs FaDu Control
**BE782190**	2.67	FaDu 10b down vs FaDu Control	CSRNP3	1.98	FaDu 10b up vs FaDu Control

**BI056255**	2.42	FaDu 10b down vs FaDu Control	CSTT	3.19	FaDu 10b up vs FaDu Control
**BM981407**	2.41	FaDu 10b down vs FaDu Control	CTSL3	5.88	FaDu 10b up vs FaDu Control
**BPI**	2.30	FaDu 10b down vs FaDu Control	CXCR2P1	4.99	FaDu 10b up vs FaDu Control
**C1QTNF8**	1.98	FaDu 10b down vs FaDu Control	CYP2G1P	2.63	FaDu 10b up vs FaDu Control
**CACNG8**	3.25	FaDu 10b down vs FaDu Control	CYP4F12	3.79	FaDu 10b up vs FaDu Control
**CAMK2B**	3.62	FaDu 10b down vs FaDu Control	CYP4F22	1.94	FaDu 10b up vs FaDu Control
**CCDC158**	2.02	FaDu 10b down vs FaDu Control	DEFA10P	4.33	FaDu 10b up vs FaDu Control
**CCDC19**	2.86	FaDu 10b down vs FaDu Control	DEFB105B	2.43	FaDu 10b up vs FaDu Control
**CCRL1**	2.28	FaDu 10b down vs FaDu Control	DEFB123	4.47	FaDu 10b up vs FaDu Control
**CD180**	3.95	FaDu 10b down vs FaDu Control	DGAT2L6	2.44	FaDu 10b up vs FaDu Control
**CD22**	3.43	FaDu 10b down vs FaDu Control	DGCR5	2.42	FaDu 10b up vs FaDu Control
**CDR1**	6.51	FaDu 10b down vs FaDu Control	DIO3OS	2.09	FaDu 10b up vs FaDu Control
**CHST5**	2.94	FaDu 10b down vs FaDu Control	DPP10	2.16	FaDu 10b up vs FaDu Control
**CLTC**	6.96	FaDu 10b down vs FaDu Control	EPYC	2.08	FaDu 10b up vs FaDu Control
**CNDP1**	2.13	FaDu 10b down vs FaDu Control	ERP27	1.91	FaDu 10b up vs FaDu Control
**CNIH2**	2.01	FaDu 10b down vs FaDu Control	ESAM	2.48	FaDu 10b up vs FaDu Control
**CNTF**	2.61	FaDu 10b down vs FaDu Control	ETG05_66023	125.46	FaDu 10b up vs FaDu Control
**CNTN6**	2.12	FaDu 10b down vs FaDu Control	FABP4	2.19	FaDu 10b up vs FaDu Control
**CRYGA**	2.01	FaDu 10b down vs FaDu Control	FAM19A4	2.06	FaDu 10b up vs FaDu Control
**CYS1**	2.42	FaDu 10b down vs FaDu Control	FAM99B	2.59	FaDu 10b up vs FaDu Control
**DA717721**	2.35	FaDu 10b down vs FaDu Control	FIGF	2.69	FaDu 10b up vs FaDu Control
**DNAH1**	3.34	FaDu 10b down vs FaDu Control	FLJ20712	2.00	FaDu 10b up vs FaDu Control
**DNASE1L3**	2.10	FaDu 10b down vs FaDu Control	FLJ39639	4.57	FaDu 10b up vs FaDu Control
**DUSP27**	5.87	FaDu 10b down vs FaDu Control	FPR2	3.08	FaDu 10b up vs FaDu Control

**EFCAB1**	8.93	FaDu 10b down vs FaDu Control	FRG2B	3.25	FaDu 10b up vs FaDu Control
**EFCAB9**	2.27	FaDu 10b down vs FaDu Control	FSD2	2.03	FaDu 10b up vs FaDu Control
**EFNA2**	1.98	FaDu 10b down vs FaDu Control	GABRA3	2.35	FaDu 10b up vs FaDu Control
**EFNA5**	2.45	FaDu 10b down vs FaDu Control	GHRH	2.00	FaDu 10b up vs FaDu Control
**EGFLAM**	2.20	FaDu 10b down vs FaDu Control	GHRLOS2	2.19	FaDu 10b up vs FaDu Control
**ESRRG**	1.97	FaDu 10b down vs FaDu Control	GJD3	5.03	FaDu 10b up vs FaDu Control
**ETV5**	2.05	FaDu 10b down vs FaDu Control	GPLD1	2.05	FaDu 10b up vs FaDu Control
**ETV7**	2.61	FaDu 10b down vs FaDu Control	GPR153	1.94	FaDu 10b up vs FaDu Control
**EXOC3L1**	2.79	FaDu 10b down vs FaDu Control	GPR22	3.17	FaDu 10b up vs FaDu Control
**FAM101A**	1.96	FaDu 10b down vs FaDu Control	GPR50	2.99	FaDu 10b up vs FaDu Control
**FAM13AOS**	2.00	FaDu 10b down vs FaDu Control	GPR63	2.14	FaDu 10b up vs FaDu Control
**FAM3D**	2.88	FaDu 10b down vs FaDu Control	GPRIN3	3.63	FaDu 10b up vs FaDu Control
**FAM48B1**	2.37	FaDu 10b down vs FaDu Control	GRIN3A	5.08	FaDu 10b up vs FaDu Control
**FAM48B2**	2.16	FaDu 10b down vs FaDu Control	GRM8	1.97	FaDu 10b up vs FaDu Control
**FCER1A**	1.95	FaDu 10b down vs FaDu Control	GSTTP1	3.30	FaDu 10b up vs FaDu Control
**FLJ16124**	2.21	FaDu 10b down vs FaDu Control	HAR1B	2.05	FaDu 10b up vs FaDu Control
**FLJ35409**	3.05	FaDu 10b down vs FaDu Control	HDX	2.87	FaDu 10b up vs FaDu Control
**FLJ41278**	2.46	FaDu 10b down vs FaDu Control	HLA-DRB6	4.49	FaDu 10b up vs FaDu Control
**FLJ41649**	2.16	FaDu 10b down vs FaDu Control	HOXC12	4.15	FaDu 10b up vs FaDu Control
**FLJ44511**	1.91	FaDu 10b down vs FaDu Control	HPVC1	2.30	FaDu 10b up vs FaDu Control
**FLJ45513**	1.92	FaDu 10b down vs FaDu Control	HSD3B2	6.85	FaDu 10b up vs FaDu Control
**FOXP4**	2.83	FaDu 10b down vs FaDu Control	HTR3E	2.08	FaDu 10b up vs FaDu Control
**FSIP1**	2.07	FaDu 10b down vs FaDu Control	IFNA21	2.25	FaDu 10b up vs FaDu Control
**GALNT4**	2.53	FaDu 10b down vs FaDu Control	IFNA4	2.08	FaDu 10b up vs FaDu Control
**GFRA4**	3.65	FaDu 10b down vs FaDu Control	IL1A	2.20	FaDu 10b up vs FaDu Control

**GIMAP8**	2.61	FaDu 10b down vs FaDu Control	IPMK	3.12	FaDu 10b up vs FaDu Control
**GJC3**	2.54	FaDu 10b down vs FaDu Control	IRGQ	2.09	FaDu 10b up vs FaDu Control
**GK2**	13.03	FaDu 10b down vs FaDu Control	KCNQ4	1.94	FaDu 10b up vs FaDu Control
**GLYATL2**	1.95	FaDu 10b down vs FaDu Control	KCNQ5	2.90	FaDu 10b up vs FaDu Control
**GML**	1.97	FaDu 10b down vs FaDu Control	KCNT2	3.07	FaDu 10b up vs FaDu Control
**GOLGA7B**	2.00	FaDu 10b down vs FaDu Control	KIAA1524	2.21	FaDu 10b up vs FaDu Control
**GPR142**	1.99	FaDu 10b down vs FaDu Control	KLHL1	4.23	FaDu 10b up vs FaDu Control
**GPR31**	2.19	FaDu 10b down vs FaDu Control	KLK7	8.75	FaDu 10b up vs FaDu Control
**GSTA2**	2.12	FaDu 10b down vs FaDu Control	KRT25	3.91	FaDu 10b up vs FaDu Control
**GUCA1C**	2.16	FaDu 10b down vs FaDu Control	KRTAP12-1	2.60	FaDu 10b up vs FaDu Control
**HEPN1**	2.08	FaDu 10b down vs FaDu Control	KRTAP27-1	13.96	FaDu 10b up vs FaDu Control
**HFM1**	2.10	FaDu 10b down vs FaDu Control	KRTAP5-3	2.97	FaDu 10b up vs FaDu Control
**HTN1**	2.58	FaDu 10b down vs FaDu Control	KRTAP7-1	11.51	FaDu 10b up vs FaDu Control
**IFNA2**	3.44	FaDu 10b down vs FaDu Control	KRTAP9-8	7.57	FaDu 10b up vs FaDu Control
**IL17RE**	1.94	FaDu 10b down vs FaDu Control	KSR1	3.62	FaDu 10b up vs FaDu Control
**IPW**	1.96	FaDu 10b down vs FaDu Control	KSR2	2.40	FaDu 10b up vs FaDu Control
**IVL**	2.76	FaDu 10b down vs FaDu Control	LCE3B	1.91	FaDu 10b up vs FaDu Control
**KCNIP2**	5.82	FaDu 10b down vs FaDu Control	LCE6A	2.55	FaDu 10b up vs FaDu Control
**KCNK10**	2.03	FaDu 10b down vs FaDu Control	LHFPL3	1.94	FaDu 10b up vs FaDu Control
**KCTD13**	2.15	FaDu 10b down vs FaDu Control	LMO2	4.33	FaDu 10b up vs FaDu Control
**KIR3DL3**	2.00	FaDu 10b down vs FaDu Control	LPAR5	1.98	FaDu 10b up vs FaDu Control
**KLK4**	2.15	FaDu 10b down vs FaDu Control	LRP1B	2.89	FaDu 10b up vs FaDu Control
**KRTAP13-4**	2.04	FaDu 10b down vs FaDu Control	MAP2	2.25	FaDu 10b up vs FaDu Control
**LAIR2**	2.03	FaDu 10b down vs FaDu Control	MATN4	2.46	FaDu 10b up vs FaDu Control
**LANCL2**	1.92	FaDu 10b down vs FaDu Control	MGC57346	1.92	FaDu 10b up vs FaDu Control

**LCE3D**	2.94	FaDu 10b down vs FaDu Control	NAALAD2	1.93	FaDu 10b up vs FaDu Control
**LRP11**	2.62	FaDu 10b down vs FaDu Control	NALCN	2.23	FaDu 10b up vs FaDu Control
**LRRN4**	2.47	FaDu 10b down vs FaDu Control	NAV2-AS4	3.88	FaDu 10b up vs FaDu Control
**MAMDC2**	3.77	FaDu 10b down vs FaDu Control	NHLH1	4.12	FaDu 10b up vs FaDu Control
**MAPRE3**	2.55	FaDu 10b down vs FaDu Control	NHLRC1	2.53	FaDu 10b up vs FaDu Control
**MEFV**	2.12	FaDu 10b down vs FaDu Control	NLGN4Y	3.47	FaDu 10b up vs FaDu Control
**METTL11B**	1.92	FaDu 10b down vs FaDu Control	NP511102	2.18	FaDu 10b up vs FaDu Control
**MGAT5**	2.11	FaDu 10b down vs FaDu Control	NR3C2	4.85	FaDu 10b up vs FaDu Control
**MIR155HG**	1.97	FaDu 10b down vs FaDu Control	NRAP	2.20	FaDu 10b up vs FaDu Control
**MTMR8**	2.09	FaDu 10b down vs FaDu Control	ODF3L1	4.49	FaDu 10b up vs FaDu Control
**MYH7**	2.26	FaDu 10b down vs FaDu Control	OR1B1	2.44	FaDu 10b up vs FaDu Control
**NCAPH**	2.50	FaDu 10b down vs FaDu Control	OR2K2	3.24	FaDu 10b up vs FaDu Control
**NFATC2**	1.98	FaDu 10b down vs FaDu Control	OR2L3	2.09	FaDu 10b up vs FaDu Control
**NP1245771**	10.09	FaDu 10b down vs FaDu Control	OR3A1	1.95	FaDu 10b up vs FaDu Control
**O3FAR1**	2.27	FaDu 10b down vs FaDu Control	OR51I2	2.10	FaDu 10b up vs FaDu Control
**OLFML2B**	2.00	FaDu 10b down vs FaDu Control	OR52E4	6.98	FaDu 10b up vs FaDu Control
**OPN1SW**	2.44	FaDu 10b down vs FaDu Control	OR52L1	3.40	FaDu 10b up vs FaDu Control
**OR10T2**	7.71	FaDu 10b down vs FaDu Control	OR5A2	2.90	FaDu 10b up vs FaDu Control
**OR4C15**	2.06	FaDu 10b down vs FaDu Control	OR5K4	5.22	FaDu 10b up vs FaDu Control
**OR51E1**	1.92	FaDu 10b down vs FaDu Control	OR6B2	12.26	FaDu 10b up vs FaDu Control
**OR51M1**	4.68	FaDu 10b down vs FaDu Control	OR8U1	2.61	FaDu 10b up vs FaDu Control
**OR52E6**	2.85	FaDu 10b down vs FaDu Control	PALM2	1.93	FaDu 10b up vs FaDu Control
**OR52N4**	8.17	FaDu 10b down vs FaDu Control	PAX4	1.94	FaDu 10b up vs FaDu Control
**OR6T1**	2.82	FaDu 10b down vs FaDu Control	PCYT1B	2.31	FaDu 10b up vs FaDu Control
**OR8K5**	5.27	FaDu 10b down vs FaDu Control	PDC	2.27	FaDu 10b up vs FaDu Control

**P2RX3**	2.08	FaDu 10b down vs FaDu Control	PHGR1	4.16	FaDu 10b up vs FaDu Control
**PCDHGA5**	2.80	FaDu 10b down vs FaDu Control	PRAMEF13	2.37	FaDu 10b up vs FaDu Control
**PEAR1**	5.73	FaDu 10b down vs FaDu Control	PRG4	12.71	FaDu 10b up vs FaDu Control
**PENK**	2.16	FaDu 10b down vs FaDu Control	PRR9	2.22	FaDu 10b up vs FaDu Control
**PIK3C2G**	1.92	FaDu 10b down vs FaDu Control	RAB43P1	2.01	FaDu 10b up vs FaDu Control
**PIP5K1P1**	3.44	FaDu 10b down vs FaDu Control	RAD21L1	2.11	FaDu 10b up vs FaDu Control
**PNLIP**	3.02	FaDu 10b down vs FaDu Control	RASSF3	2.35	FaDu 10b up vs FaDu Control
**RAP1GAP**	2.17	FaDu 10b down vs FaDu Control	RBMXL2	4.94	FaDu 10b up vs FaDu Control
**RAX**	2.92	FaDu 10b down vs FaDu Control	RBP3	5.75	FaDu 10b up vs FaDu Control
**RLN2**	2.08	FaDu 10b down vs FaDu Control	RNF152	2.15	FaDu 10b up vs FaDu Control
**RPGRIP1L**	1.92	FaDu 10b down vs FaDu Control	RNF186	3.97	FaDu 10b up vs FaDu Control
**SCN1A**	2.79	FaDu 10b down vs FaDu Control	RXFP1	2.41	FaDu 10b up vs FaDu Control
**SDR9C7**	6.30	FaDu 10b down vs FaDu Control	RYR1	2.73	FaDu 10b up vs FaDu Control
**SIRPG**	1.97	FaDu 10b down vs FaDu Control	SCGB1D2	2.24	FaDu 10b up vs FaDu Control
**SLC34A2**	5.04	FaDu 10b down vs FaDu Control	SGCA	5.69	FaDu 10b up vs FaDu Control
**SLIT2**	1.91	FaDu 10b down vs FaDu Control	SGK196	3.46	FaDu 10b up vs FaDu Control
**SMPX**	2.16	FaDu 10b down vs FaDu Control	SIM1	2.00	FaDu 10b up vs FaDu Control
**SMR3A**	1.99	FaDu 10b down vs FaDu Control	SLAIN2	1.95	FaDu 10b up vs FaDu Control
**SPATA4**	2.75	FaDu 10b down vs FaDu Control	SLC8A2	2.51	FaDu 10b up vs FaDu Control
**SPIN3**	1.94	FaDu 10b down vs FaDu Control	SPANXN5	3.59	FaDu 10b up vs FaDu Control
**SSTR5**	2.29	FaDu 10b down vs FaDu Control	SPDYE5	2.02	FaDu 10b up vs FaDu Control
**SSX5**	2.94	FaDu 10b down vs FaDu Control	STRA8	47.84	FaDu 10b up vs FaDu Control
**SYCN**	2.03	FaDu 10b down vs FaDu Control	SWT1	2.80	FaDu 10b up vs FaDu Control
**TECRL**	2.67	FaDu 10b down vs FaDu Control	TAS2R46	3.92	FaDu 10b up vs FaDu Control
**TMEM233**	6.13	FaDu 10b down vs FaDu Control	TCF23	771.04	FaDu 10b up vs FaDu Control

**TNNT3**	1.92	FaDu 10b down vs FaDu Control	TDRD6	2.63	FaDu 10b up vs FaDu Control
**TRIM78P**	2.06	FaDu 10b down vs FaDu Control	TDRG1	6.33	FaDu 10b up vs FaDu Control
**TSSK2**	2.91	FaDu 10b down vs FaDu Control	THEM5	2.13	FaDu 10b up vs FaDu Control
**TYRO3P**	11.01	FaDu 10b down vs FaDu Control	TM4SF4	3.25	FaDu 10b up vs FaDu Control
**UAP1L1**	2.82	FaDu 10b down vs FaDu Control	TMEM114	2.03	FaDu 10b up vs FaDu Control
**UPK3A**	2.29	FaDu 10b down vs FaDu Control	TMEM74	2.35	FaDu 10b up vs FaDu Control
**VSIG2**	2.08	FaDu 10b down vs FaDu Control	TMIE	5.00	FaDu 10b up vs FaDu Control
**ZBTB12**	1.94	FaDu 10b down vs FaDu Control	TREH	2.04	FaDu 10b up vs FaDu Control
**ZC3H8**	2.10	FaDu 10b down vs FaDu Control	TTC9	3.21	FaDu 10b up vs FaDu Control
**ZCCHC12**	7.63	FaDu 10b down vs FaDu Control	TTC9B	2.00	FaDu 10b up vs FaDu Control
**ZNF132**	3.49	FaDu 10b down vs FaDu Control	USP29	2.52	FaDu 10b up vs FaDu Control
**ZNF560**	2.25	FaDu 10b down vs FaDu Control	WNT7A	3.10	FaDu 10b up vs FaDu Control
**ZNF630**	3.05	FaDu 10b down vs FaDu Control	XKR5	2.92	FaDu 10b up vs FaDu Control
**ZNF699**	8.18	FaDu 10b down vs FaDu Control	XPNPEP3	2.99	FaDu 10b up vs FaDu Control
			YIPF7	5.34	FaDu 10b up vs FaDu Control
			YME1L1	2.10	FaDu 10b up vs FaDu Control
			Z39353	2.37	FaDu 10b up vs FaDu Control
			ZNF41	2.09	FaDu 10b up vs FaDu Control
			ZNF554	3.09	FaDu 10b up vs FaDu Control
			ZNF702P	2.19	FaDu 10b up vs FaDu Control
			ZNF785	2.59	FaDu 10b up vs FaDu Control
			ZNF879	1.98	FaDu 10b up vs FaDu Control
