# Supplementary material for: MicroRNA expression profile in head and neck cancer: HOX-cluster embedded microRNA-196a and microRNA-10b dysregulation implicated in cell proliferation
Source: BMC Cancer. 2013 Nov 9;13:533. doi: 10.1186/1471-2407-13-533 (PMC3826519; doi:10.1186/1471-2407-13-533)
Supplement: Additional file 5 — Differentially expressed genes between keratinocytes overexpressing miR-196a and transfection controls. [file 1471-2407-13-533-S5.pdf]

Gene Symbol	FoldChange	FoldChange Description	Gene Symbol	FoldChange	FoldChange Description
**AARS**	1.53	Keratinocyte miR-196a down vs Control	**ACVR1B**	1.53	Keratinocyte miR-196a up vs Control
**ABCA11**	1.56	Keratinocyte miR-196a down vs Control	**ADAM29**	1.56	Keratinocyte miR-196a up vs Control
**ABCA7**	1.53	Keratinocyte miR-196a down vs Control	**ADAMTS8**	1.56	Keratinocyte miR-196a up vs Control
**ACTC**	1.53	Keratinocyte miR-196a down vs Control	**ADCY1**	1.56	Keratinocyte miR-196a up vs Control
**ACTR5**	1.53	Keratinocyte miR-196a down vs Control	**ADRA2A**	1.65	Keratinocyte miR-196a up vs Control
**ADAMTS12**	1.53	Keratinocyte miR-196a down vs Control	**AGXT2L1**	1.65	Keratinocyte miR-196a up vs Control
**AGTRAP**	1.65	Keratinocyte miR-196a down vs Control	**AMZ1**	1.56	Keratinocyte miR-196a up vs Control
**AKT1**	1.53	Keratinocyte miR-196a down vs Control	**ANAPC5**	1.53	Keratinocyte miR-196a up vs Control
**ALDOAP2**	1.53	Keratinocyte miR-196a down vs Control	**ANKRD43**	1.65	Keratinocyte miR-196a up vs Control
**AOF1**	1.53	Keratinocyte miR-196a down vs Control	**ANKRD55**	1.56	Keratinocyte miR-196a up vs Control
**APOB48R**	1.56	Keratinocyte miR-196a down vs Control	**ANKS1B**	1.56	Keratinocyte miR-196a up vs Control
**ARAF**	1.53	Keratinocyte miR-196a down vs Control	**ANXA2**	1.53	Keratinocyte miR-196a up vs Control
**ARF5**	1.53	Keratinocyte miR-196a	**AQP6**	1.56	Keratinocyte miR-196a up vs

		down vs Control			Control
**ARL5B**	1.56	Keratinocyte miR-196a down vs Control	**ARL17P1**	1.53	Keratinocyte miR-196a up vs Control
**ARPC1B**	1.53	Keratinocyte miR-196a down vs Control	**ARSE**	1.56	Keratinocyte miR-196a up vs Control
**ASB8**	1.53	Keratinocyte miR-196a down vs Control	**AS3MT**	1.65	Keratinocyte miR-196a up vs Control
**ASS**	1.53	Keratinocyte miR-196a down vs Control	**ASAH3**	1.56	Keratinocyte miR-196a up vs Control
**ASTE1**	1.56	Keratinocyte miR-196a down vs Control	**AT_W_5**	1.53	Keratinocyte miR-196a up vs Control
**ATAD3B**	1.53	Keratinocyte miR-196a down vs Control	**ATXN7L2**	1.56	Keratinocyte miR-196a up vs Control
**B4GALT7**	1.56	Keratinocyte miR-196a down vs Control	**B3GNT7**	1.65	Keratinocyte miR-196a up vs Control
**BAP1**	1.65	Keratinocyte miR-196a down vs Control	**BATF**	1.65	Keratinocyte miR-196a up vs Control
**BARX1**	1.53	Keratinocyte miR-196a down vs Control	**BHLHB5**	1.56	Keratinocyte miR-196a up vs Control
**BAT5**	1.53	Keratinocyte miR-196a down vs Control	**BOC**	1.53	Keratinocyte miR-196a up vs Control
**BCL2L1**	1.53	Keratinocyte miR-196a down vs Control	**BTK**	1.65	Keratinocyte miR-196a up vs Control
**BCORL2**	1.65	Keratinocyte miR-196a down vs Control	**BYSL**	1.65	Keratinocyte miR-196a up vs Control
**BCR**	1.53	Keratinocyte miR-196a down vs Control	**BZRAP1**	1.56	Keratinocyte miR-196a up vs Control

**BIRC7**	1.53	Keratinocyte miR-196a down vs Control	**C1D**	1.55	Keratinocyte miR-196a up vs Control
**BLCAP**	1.53	Keratinocyte miR-196a down vs Control	**C1QA**	1.53	Keratinocyte miR-196a up vs Control
**BUD31**	1.53	Keratinocyte miR-196a down vs Control	**CACNA2D1**	1.53	Keratinocyte miR-196a up vs Control
**CA5B**	1.65	Keratinocyte miR-196a down vs Control	**CALML6**	1.56	Keratinocyte miR-196a up vs Control
**CACNG4**	1.65	Keratinocyte miR-196a down vs Control	**CCDC67**	1.56	Keratinocyte miR-196a up vs Control
**CAMK1**	1.65	Keratinocyte miR-196a down vs Control	**CD160**	1.56	Keratinocyte miR-196a up vs Control
**CARS**	1.53	Keratinocyte miR-196a down vs Control	**CD8A**	1.53	Keratinocyte miR-196a up vs Control
**CASP6**	1.56	Keratinocyte miR-196a down vs Control	**CDH20**	1.53	Keratinocyte miR-196a up vs Control
**CCDC32**	1.53	Keratinocyte miR-196a down vs Control	**CFC1**	1.56	Keratinocyte miR-196a up vs Control
**CCDC49**	1.53	Keratinocyte miR-196a down vs Control	**CH25H**	1.53	Keratinocyte miR-196a up vs Control
**CCDC66**	1.53	Keratinocyte miR-196a down vs Control	**CHRNA4**	1.56	Keratinocyte miR-196a up vs Control
**CCR6**	1.56	Keratinocyte miR-196a down vs Control	**CHRNB4**	1.53	Keratinocyte miR-196a up vs Control
**CCS**	1.65	Keratinocyte miR-196a down vs Control	**CIB3**	1.53	Keratinocyte miR-196a up vs Control
**CD14**	1.56	Keratinocyte miR-196a	**CLDN11**	1.56	Keratinocyte miR-196a up vs

		down vs Control			Control
**CD2BP2**	1.56	Keratinocyte miR-196a down vs Control	**CLEC4C**	1.53	Keratinocyte miR-196a up vs Control
**CD68**	1.53	Keratinocyte miR-196a down vs Control	**CLEC4G**	1.53	Keratinocyte miR-196a up vs Control
**CDC23**	1.53	Keratinocyte miR-196a down vs Control	**CML2**	1.56	Keratinocyte miR-196a up vs Control
**CDC42SE1**	1.56	Keratinocyte miR-196a down vs Control	**COL4A3**	1.56	Keratinocyte miR-196a up vs Control
**CDH7**	1.53	Keratinocyte miR-196a down vs Control	**COX4I2**	1.56	Keratinocyte miR-196a up vs Control
**CDK10**	1.53	Keratinocyte miR-196a down vs Control	**COX6A1**	1.65	Keratinocyte miR-196a up vs Control
**CDK5RAP2**	1.53	Keratinocyte miR-196a down vs Control	**CPN2**	1.56	Keratinocyte miR-196a up vs Control
**CDY2A**	1.53	Keratinocyte miR-196a down vs Control	**CRHR1**	1.56	Keratinocyte miR-196a up vs Control
**CEECAM1**	1.53	Keratinocyte miR-196a down vs Control	**CYP2A7**	1.56	Keratinocyte miR-196a up vs Control
**CENTB1**	1.53	Keratinocyte miR-196a down vs Control	**CYP2F1**	1.56	Keratinocyte miR-196a up vs Control
**CHFR**	1.53	Keratinocyte miR-196a down vs Control	**DCAL1**	1.53	Keratinocyte miR-196a up vs Control
**CHRNA6**	1.53	Keratinocyte miR-196a down vs Control	**DES**	1.53	Keratinocyte miR-196a up vs Control
**CLCN7**	1.56	Keratinocyte miR-196a down vs Control	**DGCR8**	1.56	Keratinocyte miR-196a up vs Control

**CLDN4**	1.53	Keratinocyte miR-196a down vs Control	**DLSTP**	1.56	Keratinocyte miR-196a up vs Control
**COL4A5**	1.56	Keratinocyte miR-196a down vs Control	**DPM3**	1.65	Keratinocyte miR-196a up vs Control
**COQ4**	1.53	Keratinocyte miR-196a down vs Control	**DRD3**	1.56	Keratinocyte miR-196a up vs Control
**CORO1B**	1.53	Keratinocyte miR-196a down vs Control	**DSPG3**	1.56	Keratinocyte miR-196a up vs Control
**CPSF1**	1.53	Keratinocyte miR-196a down vs Control	**ECM2**	1.65	Keratinocyte miR-196a up vs Control
**CPSF3L**	1.53	Keratinocyte miR-196a down vs Control	**EDNRB**	1.53	Keratinocyte miR-196a up vs Control
**CSPP1**	1.53	Keratinocyte miR-196a down vs Control	**EFCAB3**	1.65	Keratinocyte miR-196a up vs Control
**CTNNA3**	1.56	Keratinocyte miR-196a down vs Control	**EFNA5**	1.56	Keratinocyte miR-196a up vs Control
**DCC**	1.53	Keratinocyte miR-196a down vs Control	**EGR2**	1.53	Keratinocyte miR-196a up vs Control
**DDX56**	1.53	Keratinocyte miR-196a down vs Control	**EHD2**	1.65	Keratinocyte miR-196a up vs Control
**DEFA1**	1.65	Keratinocyte miR-196a down vs Control	**ELK4**	1.53	Keratinocyte miR-196a up vs Control
**DEFB127**	1.68	Keratinocyte miR-196a down vs Control	**EPHA6**	1.65	Keratinocyte miR-196a up vs Control
**DHPS**	1.53	Keratinocyte miR-196a down vs Control	**ERAS**	1.53	Keratinocyte miR-196a up vs Control
**DNAJB14**	1.53	Keratinocyte miR-196a	**ERVWE1**	1.56	Keratinocyte miR-196a up vs

		down vs Control			Control
**DPF2**	1.53	Keratinocyte miR-196a down vs Control	**FABP4**	1.56	Keratinocyte miR-196a up vs Control
**DPP9**	1.56	Keratinocyte miR-196a down vs Control	**FAM113A**	1.56	Keratinocyte miR-196a up vs Control
**DRG1**	1.65	Keratinocyte miR-196a down vs Control	**FAM77D**	1.56	Keratinocyte miR-196a up vs Control
**DSP**	1.56	Keratinocyte miR-196a down vs Control	**FAM81B**	1.56	Keratinocyte miR-196a up vs Control
**DTX2**	1.53	Keratinocyte miR-196a down vs Control	**FAM92B**	1.65	Keratinocyte miR-196a up vs Control
**DUSP11**	1.53	Keratinocyte miR-196a down vs Control	**FANCB**	1.65	Keratinocyte miR-196a up vs Control
**ECH1**	1.53	Keratinocyte miR-196a down vs Control	**FGA**	1.65	Keratinocyte miR-196a up vs Control
**EDC4**	1.56	Keratinocyte miR-196a down vs Control	**FKBP2**	1.56	Keratinocyte miR-196a up vs Control
**EDG4**	1.53	Keratinocyte miR-196a down vs Control	**FLCN**	1.56	Keratinocyte miR-196a up vs Control
**EFHB**	1.53	Keratinocyte miR-196a down vs Control	**FLRT1**	1.56	Keratinocyte miR-196a up vs Control
**EIF1**	1.56	Keratinocyte miR-196a down vs Control	**G36726**	1.65	Keratinocyte miR-196a up vs Control
**EIF2B5**	1.53	Keratinocyte miR-196a down vs Control	**GABRR2**	1.56	Keratinocyte miR-196a up vs Control
**ELF3**	1.53	Keratinocyte miR-196a down vs Control	**GAD1**	1.65	Keratinocyte miR-196a up vs Control

**ELOF1**	1.53	Keratinocyte miR-196a down vs Control	**GAGE3**	1.53	Keratinocyte miR-196a up vs Control
**ELOVL5**	1.53	Keratinocyte miR-196a down vs Control	**GALNTL1**	1.56	Keratinocyte miR-196a up vs Control
**EML3**	1.53	Keratinocyte miR-196a down vs Control	**GALNTL5**	1.56	Keratinocyte miR-196a up vs Control
**EPB41**	1.53	Keratinocyte miR-196a down vs Control	**GCET2**	1.65	Keratinocyte miR-196a up vs Control
**EPHA7**	1.65	Keratinocyte miR-196a down vs Control	**GCM1**	1.56	Keratinocyte miR-196a up vs Control
**ESX1**	1.53	Keratinocyte miR-196a down vs Control	**GMIP**	1.53	Keratinocyte miR-196a up vs Control
**EVC2**	1.53	Keratinocyte miR-196a down vs Control	**GNLY**	1.53	Keratinocyte miR-196a up vs Control
**EXOSC5**	1.65	Keratinocyte miR-196a down vs Control	**GPBAR1**	1.56	Keratinocyte miR-196a up vs Control
**EXPH5**	1.53	Keratinocyte miR-196a down vs Control	**GPHA2**	1.56	Keratinocyte miR-196a up vs Control
**FAH**	1.53	Keratinocyte miR-196a down vs Control	**GPHB5**	1.56	Keratinocyte miR-196a up vs Control
**FAM105B**	1.65	Keratinocyte miR-196a down vs Control	**GPR144**	1.56	Keratinocyte miR-196a up vs Control
**FAM107A**	1.56	Keratinocyte miR-196a down vs Control	**GPR31**	1.56	Keratinocyte miR-196a up vs Control
**FAM12A**	1.53	Keratinocyte miR-196a down vs Control	**GPR75**	1.65	Keratinocyte miR-196a up vs Control
**FAM73A**	1.53	Keratinocyte miR-196a	**GPR85**	1.56	Keratinocyte miR-196a up vs

		down vs Control			Control
**FBP2**	1.56	Keratinocyte miR-196a down vs Control	**GRIK2**	1.65	Keratinocyte miR-196a up vs Control
**FBXO6**	1.53	Keratinocyte miR-196a down vs Control	**GRIP2**	1.56	Keratinocyte miR-196a up vs Control
**FCGR3A**	1.65	Keratinocyte miR-196a down vs Control	**GUCA2B**	1.53	Keratinocyte miR-196a up vs Control
**FGF22**	1.65	Keratinocyte miR-196a down vs Control	**GZMM**	1.56	Keratinocyte miR-196a up vs Control
**FKBP8**	1.53	Keratinocyte miR-196a down vs Control	**HAMP**	1.65	Keratinocyte miR-196a up vs Control
**FLNA**	1.53	Keratinocyte miR-196a down vs Control	**HAVCR1**	1.65	Keratinocyte miR-196a up vs Control
**FMNL3**	1.56	Keratinocyte miR-196a down vs Control	**HIST1H2AK**	1.65	Keratinocyte miR-196a up vs Control
**FSCN1**	1.53	Keratinocyte miR-196a down vs Control	**HIST1H4K**	1.56	Keratinocyte miR-196a up vs Control
**FTSJ2**	1.53	Keratinocyte miR-196a down vs Control	**HIVEP3**	1.56	Keratinocyte miR-196a up vs Control
**FUT4**	1.56	Keratinocyte miR-196a down vs Control	**HLA-DRB3**	1.53	Keratinocyte miR-196a up vs Control
**FXR2**	1.53	Keratinocyte miR-196a down vs Control	**HMCN2**	1.53	Keratinocyte miR-196a up vs Control
**G6PD**	1.53	Keratinocyte miR-196a down vs Control	**HMP19**	1.53	Keratinocyte miR-196a up vs Control
**GBA2**	1.53	Keratinocyte miR-196a down vs Control	**HOXA7**	1.65	Keratinocyte miR-196a up vs Control

**GBL**	1.65	Keratinocyte miR-196a down vs Control	**IGSF2**	1.56	Keratinocyte miR-196a up vs Control
**GC**	1.53	Keratinocyte miR-196a down vs Control	**IGSF4D**	1.53	Keratinocyte miR-196a up vs Control
**GDDR**	1.56	Keratinocyte miR-196a down vs Control	**IL17A**	1.53	Keratinocyte miR-196a up vs Control
**GIPC1**	1.53	Keratinocyte miR-196a down vs Control	**IL28A**	1.56	Keratinocyte miR-196a up vs Control
**GJB1**	1.56	Keratinocyte miR-196a down vs Control	**INSRR**	1.53	Keratinocyte miR-196a up vs Control
**GJB5**	1.53	Keratinocyte miR-196a down vs Control	**ITGB1BP2**	1.56	Keratinocyte miR-196a up vs Control
**GLT1D1**	1.56	Keratinocyte miR-196a down vs Control	**J03651**	1.53	Keratinocyte miR-196a up vs Control
**GNA15**	1.53	Keratinocyte miR-196a down vs Control	**KCNC3**	1.56	Keratinocyte miR-196a up vs Control
**GNAI2**	1.53	Keratinocyte miR-196a down vs Control	**KCNJ1**	1.53	Keratinocyte miR-196a up vs Control
**GNB2**	1.53	Keratinocyte miR-196a down vs Control	**KCNK7**	1.56	Keratinocyte miR-196a up vs Control
**GNB2L1**	1.53	Keratinocyte miR-196a down vs Control	**KEL**	1.56	Keratinocyte miR-196a up vs Control
**GORASP1**	1.53	Keratinocyte miR-196a down vs Control	**KHSRP**	1.53	Keratinocyte miR-196a up vs Control
**GOT1L1**	1.53	Keratinocyte miR-196a down vs Control	**KIAA1456**	1.56	Keratinocyte miR-196a up vs Control
**GPAA1**	1.53	Keratinocyte miR-196a	**KIAA1661**	1.56	Keratinocyte miR-196a up vs

		down vs Control			Control
**GRAMD1A**	1.56	Keratinocyte miR-196a down vs Control	**KIR2DS2**	1.53	Keratinocyte miR-196a up vs Control
**GRIN2B**	1.56	Keratinocyte miR-196a down vs Control	**KIR3DL2**	1.65	Keratinocyte miR-196a up vs Control
**GRPEL1**	1.53	Keratinocyte miR-196a down vs Control	**KIRREL2**	1.56	Keratinocyte miR-196a up vs Control
**GTF2E2**	1.65	Keratinocyte miR-196a down vs Control	**KLHL1**	1.53	Keratinocyte miR-196a up vs Control
**H3F3B**	1.56	Keratinocyte miR-196a down vs Control	**KLHL11**	1.53	Keratinocyte miR-196a up vs Control
**HAL**	1.56	Keratinocyte miR-196a down vs Control	**KLK2**	1.65	Keratinocyte miR-196a up vs Control
**HAPLN2**	1.56	Keratinocyte miR-196a down vs Control	**KNDC1**	1.56	Keratinocyte miR-196a up vs Control
**HBLD2**	1.56	Keratinocyte miR-196a down vs Control	**LCN6**	1.56	Keratinocyte miR-196a up vs Control
**HDAC11**	1.53	Keratinocyte miR-196a down vs Control	**M69296**	1.53	Keratinocyte miR-196a up vs Control
**HEXA**	1.53	Keratinocyte miR-196a down vs Control	**MAB21L2**	1.53	Keratinocyte miR-196a up vs Control
**HEXDC**	1.65	Keratinocyte miR-196a down vs Control	**MAP3K3**	1.56	Keratinocyte miR-196a up vs Control
**HIST1H3G**	1.53	Keratinocyte miR-196a down vs Control	**MAP4K1**	1.65	Keratinocyte miR-196a up vs Control
**HKR3**	1.53	Keratinocyte miR-196a down vs Control	**MAS1L**	1.53	Keratinocyte miR-196a up vs Control

**HNRPCL1**	1.53	Keratinocyte miR-196a down vs Control	**MATK**	1.65	Keratinocyte miR-196a up vs Control
**HNRPDL**	1.53	Keratinocyte miR-196a down vs Control	**MEIS3P1**	1.65	Keratinocyte miR-196a up vs Control
**HSPB9**	1.56	Keratinocyte miR-196a down vs Control	**MGAT4C**	1.53	Keratinocyte miR-196a up vs Control
**HSPC023**	1.56	Keratinocyte miR-196a down vs Control	**MGC26647**	1.56	Keratinocyte miR-196a up vs Control
**HTN1**	1.65	Keratinocyte miR-196a down vs Control	**MGC5566**	1.53	Keratinocyte miR-196a up vs Control
**HTR2B**	1.56	Keratinocyte miR-196a down vs Control	**MIA2**	1.56	Keratinocyte miR-196a up vs Control
**HVCN1**	1.53	Keratinocyte miR-196a down vs Control	**MIDN**	1.56	Keratinocyte miR-196a up vs Control
**IDI1**	1.53	Keratinocyte miR-196a down vs Control	**MPFL**	1.56	Keratinocyte miR-196a up vs Control
**IDI2**	1.65	Keratinocyte miR-196a down vs Control	**MSR1**	1.56	Keratinocyte miR-196a up vs Control
**IDS**	1.65	Keratinocyte miR-196a down vs Control	**MTF1**	1.56	Keratinocyte miR-196a up vs Control
**IGFBPL1**	1.56	Keratinocyte miR-196a down vs Control	**MYBPC3**	1.56	Keratinocyte miR-196a up vs Control
**IL19**	1.65	Keratinocyte miR-196a down vs Control	**MYH4**	1.56	Keratinocyte miR-196a up vs Control
**INPP1**	1.65	Keratinocyte miR-196a down vs Control	**MYOZ3**	1.53	Keratinocyte miR-196a up vs Control
**IQCH**	1.56	Keratinocyte miR-196a	**N24703**	1.56	Keratinocyte miR-196a up vs

		down vs Control			Control
**IQGAP2**	1.65	Keratinocyte miR-196a down vs Control	**N75321**	1.65	Keratinocyte miR-196a up vs Control
**KATNB1**	1.53	Keratinocyte miR-196a down vs Control	**N75427**	1.65	Keratinocyte miR-196a up vs Control
**KCND1**	1.65	Keratinocyte miR-196a down vs Control	**N91552**	1.65	Keratinocyte miR-196a up vs Control
**KCNH5**	1.56	Keratinocyte miR-196a down vs Control	**NCR1**	1.56	Keratinocyte miR-196a up vs Control
**KCNK10**	1.53	Keratinocyte miR-196a down vs Control	**NHLH1**	1.53	Keratinocyte miR-196a up vs Control
**KCNMB3**	1.53	Keratinocyte miR-196a down vs Control	**NPHS2**	1.53	Keratinocyte miR-196a up vs Control
**KIAA0195**	1.53	Keratinocyte miR-196a down vs Control	**NUDT14**	1.54	Keratinocyte miR-196a up vs Control
**KIAA0404**	1.53	Keratinocyte miR-196a down vs Control	**OBFC2B**	1.65	Keratinocyte miR-196a up vs Control
**KIAA0999**	1.56	Keratinocyte miR-196a down vs Control	**OPCML**	1.65	Keratinocyte miR-196a up vs Control
**KIAA1107**	1.53	Keratinocyte miR-196a down vs Control	**OR2J2**	1.56	Keratinocyte miR-196a up vs Control
**KLC4**	1.56	Keratinocyte miR-196a down vs Control	**OR2L13**	1.56	Keratinocyte miR-196a up vs Control
**KLHDC1**	1.53	Keratinocyte miR-196a down vs Control	**OR2T8**	1.56	Keratinocyte miR-196a up vs Control
**KLHL22**	1.53	Keratinocyte miR-196a down vs Control	**OR4A15**	1.53	Keratinocyte miR-196a up vs Control

**KRTAP15-1**	1.56	Keratinocyte miR-196a down vs Control	**OR51E2**	1.56	Keratinocyte miR-196a up vs Control
**LDLRAP1**	1.53	Keratinocyte miR-196a down vs Control	**OR7E47P**	1.53	Keratinocyte miR-196a up vs Control
**LEMD2**	1.56	Keratinocyte miR-196a down vs Control	**OR8H1**	1.56	Keratinocyte miR-196a up vs Control
**LGI2**	1.56	Keratinocyte miR-196a down vs Control	**P2RX3**	1.56	Keratinocyte miR-196a up vs Control
**LINS1**	1.53	Keratinocyte miR-196a down vs Control	**PAK6**	1.53	Keratinocyte miR-196a up vs Control
**LRP6**	1.53	Keratinocyte miR-196a down vs Control	**PCDHA5**	1.53	Keratinocyte miR-196a up vs Control
**LRRC25**	1.65	Keratinocyte miR-196a down vs Control	**PCDHB7**	1.53	Keratinocyte miR-196a up vs Control
**LYPLA2**	1.53	Keratinocyte miR-196a down vs Control	**PCDHGC3**	1.56	Keratinocyte miR-196a up vs Control
**MAB21L1**	1.65	Keratinocyte miR-196a down vs Control	**PCGF1**	1.56	Keratinocyte miR-196a up vs Control
**MAP2**	1.65	Keratinocyte miR-196a down vs Control	**PGLYRP2**	1.53	Keratinocyte miR-196a up vs Control
**MAP2K1IP1**	1.53	Keratinocyte miR-196a down vs Control	**PILRB**	1.56	Keratinocyte miR-196a up vs Control
**MAPK12**	1.56	Keratinocyte miR-196a down vs Control	**PLCL2**	1.53	Keratinocyte miR-196a up vs Control
**MAPK3**	1.53	Keratinocyte miR-196a down vs Control	**PLIN**	1.65	Keratinocyte miR-196a up vs Control
**MAPKAP1**	1.53	Keratinocyte miR-196a	**PNLIP**	1.56	Keratinocyte miR-196a up vs

		down vs Control			Control
**MBNL1**	1.56	Keratinocyte miR-196a down vs Control	**PPY2**	1.56	Keratinocyte miR-196a up vs Control
**MED6**	1.56	Keratinocyte miR-196a down vs Control	**PRAMEF2**	1.65	Keratinocyte miR-196a up vs Control
**METRN**	1.56	Keratinocyte miR-196a down vs Control	**PROX1**	1.53	Keratinocyte miR-196a up vs Control
**MFN1**	1.53	Keratinocyte miR-196a down vs Control	**PTPRCAP**	1.65	Keratinocyte miR-196a up vs Control
**MGC10911**	1.65	Keratinocyte miR-196a down vs Control	**PTPRO**	1.56	Keratinocyte miR-196a up vs Control
**MGC16037**	1.53	Keratinocyte miR-196a down vs Control	**RASGEF1B**	1.65	Keratinocyte miR-196a up vs Control
**MGC17403**	1.56	Keratinocyte miR-196a down vs Control	**RASL10B**	1.53	Keratinocyte miR-196a up vs Control
**MLL**	1.56	Keratinocyte miR-196a down vs Control	**RCV1**	1.56	Keratinocyte miR-196a up vs Control
**MLL4**	1.53	Keratinocyte miR-196a down vs Control	**RNF151**	1.56	Keratinocyte miR-196a up vs Control
**MLZE**	1.53	Keratinocyte miR-196a down vs Control	**RPL23**	1.56	Keratinocyte miR-196a up vs Control
**MRPS5**	1.53	Keratinocyte miR-196a down vs Control	**RPL24**	1.53	Keratinocyte miR-196a up vs Control
**MTFMT**	1.53	Keratinocyte miR-196a down vs Control	**RPL39**	1.53	Keratinocyte miR-196a up vs Control
**MYH6**	1.65	Keratinocyte miR-196a down vs Control	**S75896**	1.56	Keratinocyte miR-196a up vs Control

**NAG8**	1.53	Keratinocyte miR-196a down vs Control	**SBEM**	1.56	Keratinocyte miR-196a up vs Control
**NMNAT1**	1.53	Keratinocyte miR-196a down vs Control	**SCAMP5**	1.56	Keratinocyte miR-196a up vs Control
**NOL10**	1.65	Keratinocyte miR-196a down vs Control	**SCGN**	1.53	Keratinocyte miR-196a up vs Control
**NOMO1**	1.53	Keratinocyte miR-196a down vs Control	**SCNN1G**	1.56	Keratinocyte miR-196a up vs Control
**NPLOC4**	1.65	Keratinocyte miR-196a down vs Control	**SEC14L3**	1.56	Keratinocyte miR-196a up vs Control
**NSDHL**	1.65	Keratinocyte miR-196a down vs Control	**SEPN1**	1.53	Keratinocyte miR-196a up vs Control
**NSFL1C**	1.53	Keratinocyte miR-196a down vs Control	**SERPINA12**	1.56	Keratinocyte miR-196a up vs Control
**NT5C2**	1.53	Keratinocyte miR-196a down vs Control	**SERPINA4**	1.53	Keratinocyte miR-196a up vs Control
**NT5C3L**	1.53	Keratinocyte miR-196a down vs Control	**SERPINI2**	1.65	Keratinocyte miR-196a up vs Control
**NT5DC2**	1.53	Keratinocyte miR-196a down vs Control	**SGEF**	1.53	Keratinocyte miR-196a up vs Control
**NUBP2**	1.56	Keratinocyte miR-196a down vs Control	**SLC11A1**	1.56	Keratinocyte miR-196a up vs Control
**NUPL2**	1.53	Keratinocyte miR-196a down vs Control	**SLC25A28**	1.53	Keratinocyte miR-196a up vs Control
**NXN**	1.53	Keratinocyte miR-196a down vs Control	**SLC2A2**	1.56	Keratinocyte miR-196a up vs Control
**OKL38**	1.53	Keratinocyte miR-196a	**SLC38A4**	1.56	Keratinocyte miR-196a up vs

		down vs Control			Control
**OSAP**	1.53	Keratinocyte miR-196a down vs Control	**SLFN13**	1.53	Keratinocyte miR-196a up vs Control
**OSGEPL1**	1.56	Keratinocyte miR-196a down vs Control	**SOLH**	1.65	Keratinocyte miR-196a up vs Control
**PACS2**	1.53	Keratinocyte miR-196a down vs Control	**SOX11**	1.53	Keratinocyte miR-196a up vs Control
**PCDHA9**	1.65	Keratinocyte miR-196a down vs Control	**SOX2OT**	1.56	Keratinocyte miR-196a up vs Control
**PDLIM2**	1.65	Keratinocyte miR-196a down vs Control	**SPDYA**	1.65	Keratinocyte miR-196a up vs Control
**PEX12**	1.53	Keratinocyte miR-196a down vs Control	**SPOCK2**	1.53	Keratinocyte miR-196a up vs Control
**PEX16**	1.53	Keratinocyte miR-196a down vs Control	**SPRR2C**	1.53	Keratinocyte miR-196a up vs Control
**PGEA1**	1.53	Keratinocyte miR-196a down vs Control	**ST6GALNAC3**	1.65	Keratinocyte miR-196a up vs Control
**PGF**	1.56	Keratinocyte miR-196a down vs Control	**STYX**	1.53	Keratinocyte miR-196a up vs Control
**PIGS**	1.53	Keratinocyte miR-196a down vs Control	**SULT4A1**	1.56	Keratinocyte miR-196a up vs Control
**PITPNM1**	1.65	Keratinocyte miR-196a down vs Control	**SYCE1**	1.53	Keratinocyte miR-196a up vs Control
**PLEKHA8**	1.53	Keratinocyte miR-196a down vs Control	**TAAR2**	1.56	Keratinocyte miR-196a up vs Control
**PLXNB1**	1.65	Keratinocyte miR-196a down vs Control	**TARP**	1.53	Keratinocyte miR-196a up vs Control

**PLXNB2**	1.56	Keratinocyte miR-196a down vs Control	**TAS1R1**	1.65	Keratinocyte miR-196a up vs Control
**PLXNB3**	1.53	Keratinocyte miR-196a down vs Control	**TBC1D21**	1.56	Keratinocyte miR-196a up vs Control
**POLDIP3**	1.53	Keratinocyte miR-196a down vs Control	**TKTL1**	1.53	Keratinocyte miR-196a up vs Control
**POLR1E**	1.53	Keratinocyte miR-196a down vs Control	**TMEM9B**	1.56	Keratinocyte miR-196a up vs Control
**POLR3K**	1.53	Keratinocyte miR-196a down vs Control	**TMPRSS9**	1.65	Keratinocyte miR-196a up vs Control
**POR**	1.56	Keratinocyte miR-196a down vs Control	**TNNT2**	1.56	Keratinocyte miR-196a up vs Control
**PPFIBP2**	1.53	Keratinocyte miR-196a down vs Control	**TNRC6C**	1.56	Keratinocyte miR-196a up vs Control
**PRKRIP1**	1.56	Keratinocyte miR-196a down vs Control	**TPH2**	1.56	Keratinocyte miR-196a up vs Control
**PROK1**	1.53	Keratinocyte miR-196a down vs Control	**TRAPPC5**	1.65	Keratinocyte miR-196a up vs Control
**PRP2**	1.53	Keratinocyte miR-196a down vs Control	**TRIM40**	1.65	Keratinocyte miR-196a up vs Control
**PRUNE**	1.53	Keratinocyte miR-196a down vs Control	**TRIM42**	1.65	Keratinocyte miR-196a up vs Control
**PSMA7**	1.53	Keratinocyte miR-196a down vs Control	**TTPA**	1.56	Keratinocyte miR-196a up vs Control
**PSMC5**	1.56	Keratinocyte miR-196a down vs Control	**TYRP1**	1.53	Keratinocyte miR-196a up vs Control
**PSMD2**	1.53	Keratinocyte miR-196a	**UBE2O**	1.65	Keratinocyte miR-196a up vs

		down vs Control			Control
**PSMF1**	1.53	Keratinocyte miR-196a down vs Control	**UGT2B10**	1.56	Keratinocyte miR-196a up vs Control
**PTPMT1**	1.53	Keratinocyte miR-196a down vs Control	**ULBP3**	1.65	Keratinocyte miR-196a up vs Control
**PVRL2**	1.53	Keratinocyte miR-196a down vs Control	**UNQ755**	1.56	Keratinocyte miR-196a up vs Control
**QARS**	1.53	Keratinocyte miR-196a down vs Control	**UPB1**	1.56	Keratinocyte miR-196a up vs Control
**RAB26**	1.56	Keratinocyte miR-196a down vs Control	**USP29**	1.56	Keratinocyte miR-196a up vs Control
**RAB5B**	1.53	Keratinocyte miR-196a down vs Control	**USP51**	1.65	Keratinocyte miR-196a up vs Control
**RABGEF1**	1.56	Keratinocyte miR-196a down vs Control	**USP9Y**	1.56	Keratinocyte miR-196a up vs Control
**RABGGTA**	1.53	Keratinocyte miR-196a down vs Control	**VAX2**	1.53	Keratinocyte miR-196a up vs Control
**RAG1AP1**	1.53	Keratinocyte miR-196a down vs Control	**VN1R1**	1.56	Keratinocyte miR-196a up vs Control
**RANGAP1**	1.53	Keratinocyte miR-196a down vs Control	**W45382**	1.56	Keratinocyte miR-196a up vs Control
**RAP1A**	1.56	Keratinocyte miR-196a down vs Control	**W95609**	1.53	Keratinocyte miR-196a up vs Control
**RGS19**	1.53	Keratinocyte miR-196a down vs Control	**WDR25**	1.65	Keratinocyte miR-196a up vs Control
**RHOT2**	1.65	Keratinocyte miR-196a down vs Control	**WDR63**	1.56	Keratinocyte miR-196a up vs Control

**RND1**	1.53	Keratinocyte miR-196a down vs Control	**WNT3**	1.56	Keratinocyte miR-196a up vs Control
**RNF150**	1.65	Keratinocyte miR-196a down vs Control	**WWC3**	1.56	Keratinocyte miR-196a up vs Control
**RPL18**	1.65	Keratinocyte miR-196a down vs Control	**ZIC2**	1.65	Keratinocyte miR-196a up vs Control
**RPL7L1**	1.65	Keratinocyte miR-196a down vs Control	**ZNF287**	1.65	Keratinocyte miR-196a up vs Control
**RPS16**	1.53	Keratinocyte miR-196a down vs Control	**ZNF420**	1.53	Keratinocyte miR-196a up vs Control
**RPS6KB1**	1.56	Keratinocyte miR-196a down vs Control	**ZNF445**	1.65	Keratinocyte miR-196a up vs Control
**RPS6KB2**	1.53	Keratinocyte miR-196a down vs Control	**ZNF473**	1.53	Keratinocyte miR-196a up vs Control
**RTP1**	1.65	Keratinocyte miR-196a down vs Control	**ZNF625**	1.56	Keratinocyte miR-196a up vs Control
**RUTBC1**	1.53	Keratinocyte miR-196a down vs Control	**ZNF676**	1.56	Keratinocyte miR-196a up vs Control
**S80491**	1.56	Keratinocyte miR-196a down vs Control	**ZNF694**	1.56	Keratinocyte miR-196a up vs Control
**SAMM50**	1.53	Keratinocyte miR-196a down vs Control	**ZP3**	1.53	Keratinocyte miR-196a up vs Control
**SBF1**	1.56	Keratinocyte miR-196a down vs Control	**ZPBP2**	1.56	Keratinocyte miR-196a up vs Control
**SC4MOL**	1.53	Keratinocyte miR-196a down vs Control			
**SEC22B**	1.53	Keratinocyte miR-196a			

		down vs Control
**SEC24C**	1.53	Keratinocyte miR-196a down vs Control
**SGPP2**	1.53	Keratinocyte miR-196a down vs Control
**SHKBP1**	1.53	Keratinocyte miR-196a down vs Control
**SIRT6**	1.56	Keratinocyte miR-196a down vs Control
**SIRT7**	1.65	Keratinocyte miR-196a down vs Control
**SLC22A6**	1.53	Keratinocyte miR-196a down vs Control
**SLC27A4**	1.53	Keratinocyte miR-196a down vs Control
**SLC2A4**	1.65	Keratinocyte miR-196a down vs Control
**SLC30A4**	1.56	Keratinocyte miR-196a down vs Control
**SLC44A5**	1.53	Keratinocyte miR-196a down vs Control
**SLC4A7**	1.53	Keratinocyte miR-196a down vs Control
**SLC5A7**	1.56	Keratinocyte miR-196a down vs Control
**SLC7A14**	1.56	Keratinocyte miR-196a down vs Control

**SLC7A7**	1.56	Keratinocyte miR-196a down vs Control
**SMPD1**	1.53	Keratinocyte miR-196a down vs Control
**SNRPA1**	1.53	Keratinocyte miR-196a down vs Control
**SNX17**	1.53	Keratinocyte miR-196a down vs Control
**SPAG16**	1.53	Keratinocyte miR-196a down vs Control
**SPAG4L**	1.56	Keratinocyte miR-196a down vs Control
**SPAG7**	1.65	Keratinocyte miR-196a down vs Control
**SPAST**	1.53	Keratinocyte miR-196a down vs Control
**SRD5A1**	1.53	Keratinocyte miR-196a down vs Control
**SRP68**	1.53	Keratinocyte miR-196a down vs Control
**SSBP4**	1.56	Keratinocyte miR-196a down vs Control
**SSX8**	1.65	Keratinocyte miR-196a down vs Control
**ST3GAL4**	1.53	Keratinocyte miR-196a down vs Control
**STAB2**	1.65	Keratinocyte miR-196a

		down vs Control
**STARD7**	1.53	Keratinocyte miR-196a down vs Control
**STK17B**	1.53	Keratinocyte miR-196a down vs Control
**STK19**	1.56	Keratinocyte miR-196a down vs Control
**STK25**	1.53	Keratinocyte miR-196a down vs Control
**STK32C**	1.65	Keratinocyte miR-196a down vs Control
**STOML3**	1.56	Keratinocyte miR-196a down vs Control
**STXBP2**	1.53	Keratinocyte miR-196a down vs Control
**SUMO1**	1.65	Keratinocyte miR-196a down vs Control
**SUV420H2**	1.56	Keratinocyte miR-196a down vs Control
**TAPBP**	1.53	Keratinocyte miR-196a down vs Control
**TAX1BP3**	1.56	Keratinocyte miR-196a down vs Control
**TBC1D5**	1.53	Keratinocyte miR-196a down vs Control
**TBL3**	1.53	Keratinocyte miR-196a down vs Control

**TEC**	1.65	Keratinocyte miR-196a down vs Control
**THAP7**	1.56	Keratinocyte miR-196a down vs Control
**THOC5**	1.53	Keratinocyte miR-196a down vs Control
**THSD3**	1.65	Keratinocyte miR-196a down vs Control
**THUMPD3**	1.53	Keratinocyte miR-196a down vs Control
**TJP3**	1.53	Keratinocyte miR-196a down vs Control
**TM4SF1**	1.53	Keratinocyte miR-196a down vs Control
**TMEM16J**	1.53	Keratinocyte miR-196a down vs Control
**TMEM68**	1.56	Keratinocyte miR-196a down vs Control
**TMEM85**	1.56	Keratinocyte miR-196a down vs Control
**TMPRSS11E**	1.53	Keratinocyte miR-196a down vs Control
**TNIP2**	1.53	Keratinocyte miR-196a down vs Control
**TNRC5**	1.53	Keratinocyte miR-196a down vs Control
**TNT**	1.56	Keratinocyte miR-196a

		down vs Control
**TOM1**	1.53	Keratinocyte miR-196a down vs Control
**TPCN2**	1.53	Keratinocyte miR-196a down vs Control
**TSR2**	1.53	Keratinocyte miR-196a down vs Control
**TTC4**	1.53	Keratinocyte miR-196a down vs Control
**TTC5**	1.53	Keratinocyte miR-196a down vs Control
**TTF1**	1.53	Keratinocyte miR-196a down vs Control
**TTTY21**	1.65	Keratinocyte miR-196a down vs Control
**TUBGCP2**	1.53	Keratinocyte miR-196a down vs Control
**TUBGCP6**	1.65	Keratinocyte miR-196a down vs Control
**TXK**	1.56	Keratinocyte miR-196a down vs Control
**TXNDC12**	1.53	Keratinocyte miR-196a down vs Control
**TYSND1**	1.53	Keratinocyte miR-196a down vs Control
**UCKL1**	1.56	Keratinocyte miR-196a down vs Control

**ULK3**	1.53	Keratinocyte miR-196a down vs Control
**USP19**	1.56	Keratinocyte miR-196a down vs Control
**USP5**	1.53	Keratinocyte miR-196a down vs Control
**VPS13B**	1.53	Keratinocyte miR-196a down vs Control
**VRK3**	1.53	Keratinocyte miR-196a down vs Control
**WDR27**	1.53	Keratinocyte miR-196a down vs Control
**WDR42A**	1.53	Keratinocyte miR-196a down vs Control
**WFIKKN1**	1.53	Keratinocyte miR-196a down vs Control
**YARS**	1.53	Keratinocyte miR-196a down vs Control
**YRDC**	1.56	Keratinocyte miR-196a down vs Control
**ZBTB25**	1.65	Keratinocyte miR-196a down vs Control
**ZBTB38**	1.56	Keratinocyte miR-196a down vs Control
**ZC3H3**	1.65	Keratinocyte miR-196a down vs Control
**ZC3H7A**	1.53	Keratinocyte miR-196a

		down vs Control
**ZFAND1**	1.65	Keratinocyte miR-196a down vs Control
**ZMYM6**	1.53	Keratinocyte miR-196a down vs Control
**ZNF175**	1.56	Keratinocyte miR-196a down vs Control
**ZNF263**	1.56	Keratinocyte miR-196a down vs Control
**ZNF343**	1.53	Keratinocyte miR-196a down vs Control
**ZNF461**	1.56	Keratinocyte miR-196a down vs Control
**ZNF514**	1.53	Keratinocyte miR-196a down vs Control
**ZNF547**	1.65	Keratinocyte miR-196a down vs Control
**ZNF552**	1.53	Keratinocyte miR-196a down vs Control
**ZNF563**	1.56	Keratinocyte miR-196a down vs Control
**ZNF57**	1.53	Keratinocyte miR-196a down vs Control
**ZNF570**	1.65	Keratinocyte miR-196a down vs Control
**ZNF597**	1.53	Keratinocyte miR-196a down vs Control

**ZNF70**	1.53	Keratinocyte miR-196a down vs Control
**ZNF702**	1.65	Keratinocyte miR-196a down vs Control
**ZNF718**	1.53	Keratinocyte miR-196a down vs Control
